# In-vitro antiproliferative evaluation of newly synthesized titanium(IV) metallacyclic complexes on HeLa and MCF7 cell lines

**DOI:** 10.1038/s41598-025-13995-0

**Published:** 2025-08-08

**Authors:** Shivabasayya V. Salimath, Kavita B. Hiremath, Sathish Thanigachalam, Arjita Ghosh, Selva Kumar Ramasamy, Anbalagan Moorthy, S. K. Ashok Kumar, Murugesh Shivashankar, Madhvesh Pathak

**Affiliations:** 1 Department of Chemistry, School of Advanced Sciences (SAS), Vellore Institute of Technology (VIT), Vellore, Tamil Nadu India; 2 Department of Integrative Biology, School of Bioscience and Technology (SBST), Vellore Institute of Technology (VIT), Vellore, Tamil Nadu India; 3 Department of Chemistry, M.M. Engineering College, Maharishi Markandeshwar (Deemed to be University), Mullana, Ambala, Haryana India

**Keywords:** Chemical biology, Drug discovery

## Abstract

A series of eight metalacyclic derivatives of titanium i.e. [**(acac)Ti(ph)L**] (**TiC1–TiC7**) and [**LTiL**] (**TiC8**) (where acacH = acetylacetone, ph = phenol derivatives and L = ligand L1) were synthesized in anhydrous media and were characterized employing NMR (^1^H and^13^C), UV-Vis, FT-IR and ESI-mass analyses. Viscosity assays, absorption and fluorescence spectroscopy and molecular docking studies have been extrapolated to investigate the complexes’ interaction with CT-DNA and bovine serum albumin (BSA). Interaction of the complexes with CT-DNA was validated as groove binding mode using UV-Vis absorption spectra as evidenced by their intrinsic binding constants of all wherein **TiC1** and **TiC2** exhibited higher values (19.4 × 10^5^ and 5.50 × 10^5^ M^− 1^) of K_b_. Gel electrophoresis assay displayed that CT-DNA had a limited cleavage ability when metal complexes with different activators were present. Fluorescence technique also verified the interaction of the complexes with CT-DNA and BSA to demonstrate greater K_b_ values (2.84 × 10^4^ and 6.42 × 10^4^ M^− 1^) of the derivatives **TiC1** and **TiC2**. DPPH assay was carried out to determine the radical scavenging activity of the complexes, so in-vitro cytotoxic effect on malignant cell lines was investigated by MTT Assay with MCF7 and HeLa cell lines, where complexes were observed as potent towards the HeLa cell line only. The potent **TiC2** and **TiC8** were screened with MTT assay against a normal HEK-293 cell line and were found to be inactive as expected. Subsequently, dual staining with acridine orange (AO) and ethidium bromide (EB) was performed to determine the cell death. Eventually, intracellular ROS was determined by DCFDA staining followed by cell cycle analysis was carried out using propidium iodide (PI) by flow cytometry to determine the phases.

**Supplementary Information:**

The online version contains supplementary material available at 10.1038/s41598-025-13995-0.

## Introduction

Schiff bases (SB) are a dominant class of chemical compounds possessing various medicinal properties and are also well-known for their powerful chelating characteristics while making complexes with metals to uphold a top position in coordination chemistry^1–3^. Such complexes play a vital role in the domains of chemistry, biology and industry^4–7^. Complexes associated with Schiff base are considered among the most significant bioactive agents^8,9^. The chelation between the ligand and metal contributes considerably to the elevation of biopotential manifolds compared to their free states. Applications of SB in medicinal chemistry^10,11^ catalysis^12–14^ and synthesis of transition metal complexes^15^ have encompassed diverse interests in biochemical, clinical and pharmacological areas^16^. It has also been established that an imine group is necessary for these complexes to function biologically. Furthermore, they are essential in comprehending the biological processes of transformation and racemization reactions and can be found in a wide range of natural and non-natural materials^7,17–19^.

Platinum derivatives i.e. cisplatin^20^ carboplatin (paraplatin)^21^ and oxaliplatin (eloxatin)^22^ are used to treat 40 − 80% of cancer patients alone or in combination with chemotherapy^23–25^. Platinum complexes have been utilized in metallodrug-based chemotherapy for decades because of their potent cytotoxic effects^26^.

On the other hand, interest in the utilization of different metal complexes with comparable antineoplastic characteristics and greater potential for human application has grown due to the comparatively high number of side effects linked with platinum therapy^27,28^. Thus, in recent years, the use of alternative metal complexes in preclinical trials during in-vitro studies has produced excellent results^29,30^.

Worldwide, researchers are deeply involved in investigating other metals e.g. gold^31^ ruthenium^32^ iron^33^ copper^34^ osmium^35^ rhenium^36^ and iridium^37^ complexes of high significance regarding metal-based anticancer drugs. Hence the mission to develop Ti(IV) complexes as effective anticancer drugs has gone expedited presently. Further, their broader range of activity and lack of cross-resistance with platinum-based anticancer treatments have aligned them also in the fraternity of the main stream of commercial drugs floated in the market with exceptional potential^38–40^.

On the other hand, in spite of remarkable cytotoxic characteristic, titanocene dichloride the first metallocene exhibiting carcinogenic potential and budotitane also reported by Prof. Dr. Hartmut Köpf and Dr. Petra Köpf-Maier could not stay on the required standards during their clinical trials because of their low potency. In fact, the main drawbacks of the titanocene dichloride include instability at prolonged hydrolysis and poor water solubility. Researchers concerned have made prominent changes in the molecules while substituting varied ligands that enable them less labile with greater cellular specificity and biocompatibility^41,42^.

Subsequently, McGowan and Lord created and annotated a library of second-generation budotitane complexes by altering electron-withdrawing substituents to the *β*-diketonate ligands^43^ as they demonstrated enhanced cytotoxicity with better aqueous stability. In search of better Ti(IV) drugs, Tshuva and Huhn came up with a new class of ligands such as N, N′-ethylenebis(salicylimine) (salen)^41^ and tetradentate diamino bis(phenolato) (salan)^44^ and their Ti(IV) complexes have emerged as noticeably more cytotoxic, soluble and stabler in solution^45–47^.

Even though the design of Ti(IV) alkoxide anticancer complexes has advanced considerably, these complexes still have certain flaws. For example, in-vivo experiments demonstrated that some of these derivatives have a little tumor accumulation even with greater stability, but lack of knowledge regarding their mode of action makes it complicated to design ligands for increased activity. On the other hand, the ligands have greater capacity to contribute actively towards the cytotoxicity of the complexes. Using enhanced comprehension of Ti(IV) behaviour under physiological circumstances and its complex biochemistry, ligands could be chosen and designed to support the cytotoxic characteristics of titanium^48–50^.

Targeting DNA is crucial in the treatment of hereditary illnesses, most notably cancer. The significance of transition metal complexes in clinical therapy has led to a surge in the biological study of these compounds as potential anticancer medications^51^. An initial indication of a potential drug for usage as an anticancer agent appears from its mode of DNA interaction. Therefore, transition metal complexes of diverse spectral and electrochemical characteristics are made to improve their DNA binding and cleavage capabilities in both eukaryotes and prokaryotes. Topoisomerases are crucial enzymes for DNA replication, transcription, recombination, amplification and chromosome separation^52^ where topoisomerase II is capable of breaking double DNA strands while topoisomerase, I hold its ability to break single DNA strand^53,54^.

Several therapeutically utilized anticancer medications have been identified to inhibit topoisomerases such as daunomycin and bleomycin. The primary cause of oxidative damage of DNA, lipids and proteins is the excess of activated oxygen species produced during regular metabolic processes. Consequently, other ailments are also triggered including cancer, aging, inflammation, cardiovascular disease and neurological disorders. Metal complexes could function as antioxidants according to their specific structures and induce oxidative stress^55,56^.

Serum albumin - human serum albumin (HSA)^57^ and bovine serum albumin (BSA) are the main plasma proteins in the bloodstream that significantly increase the reversible binding and delivery of a wide range of endogenous and exogenous drug entities. Drug–BSA interaction studies are therefore regarded as an essential step in evaluating the therapeutic efficacy and pharmacological response of the drug in the body because they offer a deeper understanding of the absorption, transportation, distribution, metabolism and excretion properties of the drug^58^.

BSA has been used as a carrier conjugate of several anticancer metal complexes because it was considered to be a potential method to improve the specific target of the drug. BSA as a potential medication can have a significant effect on its bioavailability, lengthen its half-life during in-vivo studies and suppresses it from penetrating passively the target tissues^59,60^.

Thus, in interest of above said reports, the present article elaborates on the eight new hexacoordinated Ti(IV) complexes soluble fairly in DMSO. After proper synthesis and well characterization of these complexes, the main goal was to assess their possible anticancer efficacy at different doses during in-vitro experiments such as BSA - DNA interactions, site marker and synchronous studies followed by molecular docking and density functionality theorem (DFT) to determine the binding modes of molecules with BSA-DNA and molecular insights. Eventually, cytotoxicity tests were also performed to evaluate the most likely apoptotic mechanistic pathways ascertaining the chemotherapeutic potential through dual staining, cell cycle analysis and ROS generation of these Ti(IV) derivatives.

## Experimental section

### Physical measurements

ATR mode infrared (4000 –400 cm^− 1^) spectrophotometer thermoscientific Nicolet iS50 was used to identify the stretching frequencies of characteristic functional groups of the new derivatives of titanium(IV) ^1^H, ^13^C, ^19^F and DEPT-135 NMR spectra were recorded at Bruker Avance 400 MHz spectrometer taking Si(CH_3_)_4_ as an internal standard and DMSO-d_6_ solvent for the complexes. Electron impact mass spectra have been run on a Waters technologies Xevo G2-XS mass spectrometer. UV titrations were performed at Jasco V-670 in the range 200–600 nm while fluorescence titrations were carried out with the help of Hitachi F-7000 under 200–800 nm. Cyclic voltammograms were obtained using cyclic voltammetry of C-H Instruments, Elico CM180 in conductivity analysis, *AXYGEN* gel documentation system to capture gel electrophoresis images, Bio-Rad xMARKTM Microplate Spectrophotometer to record UV measurements for DPPH and MTT assay, Olympus Confocal Laser Scanning Microscope- Fluoview Fv3000 to capture images of cell lines and CytoFlex S Beckman Coulter flow cytometer was used to determine cell cycle analysis.

### Materials and methods

In view of synthesis and binding investigations, the analytical and spectroscopic grades chemicals and solvents were utilized. Titanium tetraisopropoxide, 5-bromo-salicylaldehyde, 2-amino-5-methylphenol and acetylacetone were procured from Sigma Aldrich and phenol derivatives were outsourced from AVRA synthesis and SRL chemicals and were used without any further purification. Calf thymus DNA (CT-DNA), 2,2-diphenyl-1-picrylhydrazyl (DPPH) were purchased from Sigma Aldrich, Bovine serum albumin (BSA), (3-(4,5-dimethylthiazol-2-yl)-2,5-diphenyltetrazolium bromide) MTT, acridine orange (AO), ethidium bromide (EtBr), propidium iodide (PI) and 2′,7′-dichlorofluorescein diacetate (DCFHDA) were got from Himedia and SRL Chemicals. Solvents were purchased from SRL chemicals and were subjected for standard purification and drying before using in the experiments because synthesis of all the complexes was carried out under anhydrous media only.

### Preparation of 4-bromo-2-{[(2-hydroxy-4-methylphenyl)iminio]methyl}phenol ligand [L1]

4-Bromo-2-{[(2-hydroxy-4-methylphenyl)iminio]methyl}phenol ligand (L1) was prepared by treating methanolic solutions of 5-bromosalicylaldehyde (15.0 mmol, 3.0 gram) and 2-amino-5-methyl phenol (15.0 mmol, 1.837 gram) in equimolar ratios. The desired ligand appeared in good yield as orange solid after stirring the reactants for 8 h at room temperature. The crude product was filtered and washed twice with distilled water followed by cold methanol too. The orange ligand was recrystallized using ethanol-water medium and was characterized† by FTIR, NMR^1^H and^13^C), ESI-MS and single crystal X-ray diffraction (SCXRD) [Electronic Supporting Information (**ESI†**)].

Yield: 95%; Orange solid; m.p 204–205 °C^1^H NMR (DMSO-d_6_, 400 MHz, δ-ppm): 2.257 ( S 3 H, CH_3_), 6.67 (s, 1 H, CHAr), 6.787 (s, 1 H, CHAr), 6.890–6.909 ( d,1 H, j = 3.8hz, CHAr), 7.265–7.279 (d, 1 H, j = 2.8hz, CHAr), 7.478–7.497 (d, 1 H, j = 3.8hz, CHAr), 7.820 (d, 1 H, CHAr), 8.953 (s, 1 H, N = CH), 9.742 (s, 1 H, C─OH), 14.001(s, 1 H, C─OH), ESI Fig. S1†^13^C NMR (DMSO‐d_6_, 100 MHz, δ, ppm): 21.36 (*β*‐CH_3_), 109.79, 117.53, 119.47, 119.62, 120.84, 121.85, 132.15, 134.15, 135.28, 138.85, 151.73, 159.09, 160.45(C═N), ESI Fig. S2†. Anal. Calcd for C_14_H_12_BrNO_2_ (%): C, 54.92; H, 3.95; N, 4.58. Found (%): C, 54.68; H, 4.12; N, 4.32. FTIR (ATR, cm^− 1^): 3039.90 (OH), 2821.11(C-H _Aromatic_) 1600.57 (C═N amide), 1285.17 (C-CH_3 aliphatic_), 799.39 (C-H), 618.35 (C-Br), ESI Fig. S3†. UV-Vis: 490 nm ESI Fig. S4†. SCXRD: 305.13 (M^+^) ESI Fig. S5-S6 and Table S4-S10†.

### Synthesis of complexes (1–8)

Titanium complexes (1–7) were synthesized by treating titanium isopropoxide Ti(O^*i*^Pr)_4_ (1.0 mmol, 285 mg) in stoichiometric amounts of synthesized ligand **(L1)** (1.0 mmol, 306 mg), acetylacetone **(L2)** (1.0 mmol, 100 mg) and phenol **(L3)** (1.0 mmol, 94 mg) in anhydrous THF and the reaction mixture was refluxed for 8 h at 80 °C. Similarly, complex-8 was synthesized using Ti(O^*i*^Pr)_4_ and a couple of moles of **L1** (1.0 mmol, 306 mg) in dried benzene. All the new derivatives of titanium(IV) were isolated under reduced pressure and subsequently washed thrice with n-hexane to afford brown solid after vacuum drying properly. Purified products were characterized using FTIR, NMR ^1^H, ^13^C, ^19^F and DEPT-135) and ESI-MS†.

### **[(acac)Ti(ph-4-OMe)L] (TiC1)**

Yield 95%; Brown; m.*p* > 300 °C^1^H NMR (DMSO-d_6_, 400 MHz, δ, ppm): 2.068 ( s 3 H, CH_3_), 2.184 ( s 3 H, CH_3_), 2.301 ( s 3 H, CH_3_), 3.734 ( s 3 H, O-CH_3_), 5.721 ( s 1 H), 6.719–6.801 (q, 4 H, j = 4.0 Hz, CHAr), 6.879 (S, 1 H, CHAr), 7.038–7.061 ( 1 H, j = 3.8hz, CHAr), 7.195–7.214 (d, 1 H, j = 3.8hz, CHAr), 7.411 (s, 1 H, CHAr), 7.681–7.688 (d, 1 H, J = 1.4 Hz, CHAr), 7.702–7.783 (d, 1 H, J = 2.6 Hz, CHAr), 10.271 (s, 1 H, N = CH), ESI Fig. S7†^13^C NMR (DMSO‐d_6_, 100 MHz, δ, ppm): 20.65 (β‐CH_3_),24.82, 31.00, 55.65, 58.16, 100.87, 111.27, 114.14, 114.99, 116.13, 117.01, 119.88, 130.94, 139.73, 151.56, 152.56(C═N), 159.07, 159.45, 191.58 and 203.80, ESI Fig. S8†. DEPT-135 NMR (DMSO‐d_6_, 100 MHz, δ, ppm): 55.65 C-H, ESI Fig. S9†. **Anal. Calcd** for C_26_H_24_BrNO_6_Ti (%): C, 54.38; H, 4.21; N, 2.44. Found (%): C, 54.14; H, 4.35; N, 2.56. FTIR (ATR, cm^− 1^): 2919.01 (CH), 1577.96 (C═N amide), 1527.25 (C═O), 1495.62 (C-C), 1361.98 (C-CH_3 aliphatic_), 1217.34, 1027.23, 817.18 (C-H), 672.55, 606.53 (C-Br), 533.72 (Ti-N), 458.49 (Ti-O), ESI Fig. S10†. Electrospray ionization ESI‐MS (m/z): Expected value 575.0246; Obtained value 575.1337(M +), ESI Fig. S11†.

### **[(acac)Ti(ph-4-Cl)L] (TiC2)**

Yield 82%; Brown; m.*p* > 300 °C^1^H NMR (DMSO-d_6_, 400 MHz, δ, ppm): 1.940 ( s 3 H, CH_3_), 2.071 ( s 3 H, CH_3_), 2.203 ( s 3 H, CH_3_), 5.579 ( s 1 H), 6.622–6.645 (d, 2 H, j = 4.6 Hz, CHAr), 6.712–6.736 (d, 1 H, j = 4.8 Hz, CHAr), 6.813 ( 1 H, CHAr), 6.925–6.944 (d, 1 H, j = 3.8hz, CHAr),7.089–7.136 (d, 2 H, J = 9.4 Hz, CHAr), 7.535–7.565 (d, 2 H, J = 6 Hz, CHAr), 7.702–7.709 (d, 1 H, J = 1.4 Hz, CHAr),10.165 (s, 1 H, N = CH), ESI Fig. S12†^13^C NMR (DMSO‐d_6_, 100 MHz, δ, ppm): 20.65 (β‐CH_3_),24.33, 100.57, 110.97, 111.04, 113.90, 166.35,116.76,116.95,117.16, 119.61, 120.04, 120.30, 124.04, 129.28, 131.35, 138.64, 139.78, 150.86, 158.66(C═N),159.05,159.44,160.28, and 180.13, ESI Fig. S13†. DEPT-135 NMR (DMSO‐d_6_, 100 MHz, δ, ppm): 57.88 C-H, ESI Fig. S14†. **Anal. Calcd** for C_25_H_21_BrClNO_5_Ti (%): C, 51.89; H, 3.66; N, 2.42. Found (%): C, 52.12; H, 3.72; N, 2.39. FTIR (ATR, cm^− 1^): 2920.33 (CH), 1580.21 (C═N amide), 1530.12 (C═O), 1480.12 (C-C), 1340.12 (C-CH_3 aliphatic_),1260.35, 857.26 (C-H), 674.33, 606.12 (C-Br), 533.36 (Ti-N), 452.96 (Ti-O), ESI Fig. S15†. Electrospray ionization ESI‐MS (m/z): Electrospray ionization ESI‐MS (m/z): Expected value 576.9771; Obtained value 577.8603 (M + H), ESI Fig. S16†.

### **[(acac)Ti(ph-4-Br)L] (TiC3)**

Yield 89%; Brown; m.*p* > 300 °C^1^H NMR (DMSO-d_6_, 400 MHz, δ, ppm): 1.940 ( s 3 H, CH_3_), 2.071 ( s 3 H, CH_3_), 2.203 ( s 3 H, CH_3_), 5.579 ( s 1 H), 6.622–6.633 (d, 1 H, j = 2.2 Hz, CHAr), 6.676-6.700 (d, 1 H, j = 4.8 Hz, CHAr), 6.801 ( 1 H, CHAr), 6.926–6.951 (d, 2 H, j = 5 Hz, CHAr),7.104–7.112 (d, 2 H, J = 2 Hz, CHAr), 7.224–7.246 (d, 1 H, J = 4.4 Hz, CHAr), 7.538–7.567 (d, 1 H, J = 5.8hz, CHAr),10.165 (s, 1 H, N = CH), ESI Fig. S17†^13^C NMR (DMSO‐d_6_, 100 MHz, δ, ppm): 20.67 (β‐CH_3_),24.57, 111.04, 113.90, 116.75, 116.95, 117.79, 119.61, 120.05, 120.31, 124.21, 131.34, 132.22, 138.64, 139.78, 150.86, 158.27(C═N), 159.05, 159.43, 160.28, 170.99, 185.70, and 190.67, ESI Fig. S18†. DEPT-135 NMR (DMSO‐d_6_, 100 MHz, δ, ppm): 57.99 C-H, ESI Fig. S19†. **Anal. Calcd** for C_25_H_21_Br_2_NO_5_Ti (%): C, 48.19; H, 3.40; N, 2.25. Found (%): C, 49.02; H, 3.45; N, 2.65. FTIR (ATR, cm^− 1^): 2921.68 (OH), 1577.96 (C═N amide), 1529.75 (C═O), 1529.31 (C-C), 1462.73 (C-CH_3 aliphatic_), 1291.90, 1177.81, 817.18 (C-H), 672.59, 606.52 (C-Br), 532.73 (Ti-N), 459.52 (Ti-O), ESI Fig. S20†.Electrospray ionization ESI‐MS (m/z): Electrospray ionization ESI‐MS (m/z): Expected value 620.9266; Obtained value 620.0165, 620.9182 (M +), ESI Fig. S21†.

### **[(acac)Ti(ph-4-F)L] (TiC4)**

Yield 86%; Brown; m.*p* > 300 °C^1^H NMR (DMSO-d_6_, 400 MHz, δ, ppm): 2.014 ( s 3 H, CH_3_), 2.116 ( s 3 H, CH_3_), 2.251 ( s 3 H, CH_3_), 5.639 ( s 1 H), 6.671–6.679 (d, 1 H, j = 1.6 Hz, CHAr), 6.691–6.751 (d, 2 H, j = 12 Hz, CHAr), 6.826 ( 1 H, CHAr), 6.908-6. 995 (d, 3 H, j = 17.4 Hz, CHAr),7.132–7.164 (d, 1 H, J = 6.4 Hz, CHAr), 7.609–7.636 (d, 1 H, J = 5.4 Hz, CHAr), 7.725–7.732 (d, 1 H, J = 1.4 Hz, CHAr),10.211 (s, 1 H, N = CH), ESI Fig. S22†^13^C NMR (DMSO‐d_6_, 100 MHz, δ, ppm): 21.11(β‐CH_3_),24.83, 31.00, 58.17, 100.88, 111.25, 114.11, 115.84, 115.84, 116.07, 116.43, 116.51, 116.98, 117.93, 119.84, 124.20, 124.20, 130.94, 136.78, 138.87, 139.73, 151.03, 154.08, 154.79, 157.10(C═N), 159.45, and 190.18, ESI Fig. S23†. DEPT-135 NMR (DMSO‐d_6_, 100 MHz, δ, ppm): 58.17 C-H, ESI Fig. S24†^19^. F NMR (DMSO‐d_6_, 100 MHz, δ, ppm): -77.62, ESI Fig. S25†. **Anal. Calcd** for C_25_H_21_BrFNO_5_Ti (%): C, 53.41; H, 3.77; N, 2.49. Found (%): C, 53.26; H, 3.85; N, 2.44. FTIR (ATR, cm^− 1^): 2914.66 (CH), 1577.04 s (C═N amide), 1529.75 (C═O), 1492.66 (C-C), 1364.92 (C-CH_3 aliphatic_), 1290.14, 1177.81, 815.25 (C-H), 672.07, 606.02 (C-Br), 533.22 (Ti-N), 460.90 (Ti-O), ESI Fig. S26†. Electrospray ionization ESI‐MS (m/z): Expected value 561.0067; Obtained value 561.1266 (M +), ESI Fig. S27†.

### **[(acac)Ti(ph-4-tBu)L] (TiC5)**

Yield 91%; Brown; m.*p* > 300 °C^1^H NMR (DMSO-d_6_, 400 MHz, δ, ppm): 2.037 ( s 9 H, CH_3_), 2.171 ( s 6 H, CH_3_), 2.275 ( s 3 H, CH_3_), 5.672 ( s 1 H), 6.713–6.729 (d, 1 H, j = 3.2 Hz, CHAr), 6.791–6.817 (d, 2 H, j = 5.2 Hz, CHAr), 6.883 ( 1 H, CHAr), 7.0165–7.042 (d, 1 H, j = 5.2 Hz, CHAr),7.165-7.200 (t, 3 H, J = 7 Hz, CHAr), 7.638–7.646 (d, 1 H, J = 1.6 Hz, CHAr), 7.661–7.668 (d, 1 H, J = 1.4 Hz, CHAr), 7.782–7.790 (d, 1 H, J = 1.6 Hz, CHAr),10.250 (s, 1 H, N = CH), ESI Fig. S28†^13^C NMR (DMSO‐d_6_, 100 MHz, δ, ppm): 20.76(β‐CH_3_), 31.41,33.75, 79.08, 110.98, 113.84, 114.95, 115.84, 115.84, 116.69, 116.95, 119.91,120.28, 123.93,126.09,131.76, 138.63, 139.79,141.46,150.79, 155.11, 158.31(C═N), 158.69, 159.08, 159.45, 160.26 and 191.17, ESI Fig. S29†. DEPT-135 NMR (DMSO‐d_6_, 100 MHz, δ, ppm): 39.30 C-H, ESI Fig. S30†. **Anal. Calcd** for C_29_H_30_BrNO_5_Ti (%): C, 58.02; H, 5.02; N, 2.33. Found (%): C, 57.85; H, 5.14; N, 2.43. FTIR (ATR, cm^− 1^): 2910.53 (CH), 1578.45 (C═N amide), 1529.74 (C═O), 1496.48 (C-C), 1362.94 (C-CH_3 aliphatic_), 1263.14, 816.51 (C-H), 672.55, 606.02 (C-Br), 534.18 (Ti-N), 460.90 (Ti-O), ESI Fig. S31†. Electrospray ionization ESI‐MS (m/z): Expected value 600.3320; Obtained value 600.4762 (M+), ESI Fig. S32†.

### **[(acac)Ti(ph-4-Me)L] (TiC6)**

Yield 95%; Brown; m.*p* > 300 °C^1^H NMR (DMSO-d_6_, 400 MHz, δ, ppm): 1.906 ( s 3 H, CH_3_), 2.048 ( s 3 H, CH_3_), 2.095 ( s 3 H, CH_3_), 2.176( s 3 H, CH_3_), 5.530 ( s 1 H), 7.087–7.105 (d, 1 H, j = 3.6 Hz, CHAr), 7.263 (S, 1 H, CHAr), 7.506–7.534 (dd 1 H, j = 1.8hz, CHAr), 7.687–7.696. (d, 1 H, J = 1.8hz, CHAr), 10.105 (s, 1 H, N = CH), ESI Fig. S33†^13^C NMR (DMSO‐d_6_, 100 MHz, δ, ppm): 20.65 (β‐CH_3_),19.85, 20.56, 24.22, 30.57, 100.42, 110.94, 111.03, 113.88, 115.33, 116.92, 119.96, 120.27, 123.98, 124.08, 128.43, 129.86, 131.54, 138.62, 139.82, 150.83, 153.28(C═N), 155.29, 158.30, 158.69, 159.08, 159.47, 160.25, 190.95, ESI Fig. S34†. DEPT-135 NMR (DMSO‐d_6_, 100 MHz, δ, ppm): 57.51 C-H, ESI Fig. S35†. **Anal. Calcd** for C_26_H_24_BrNO_5_Ti (%): C, 55.94; H, 4.33; N, 2.51. Found (%): C, 56.22; H, 4.42; N, 2.34. FTIR (ATR, cm^− 1^): 2965.32 (CH), 1606.32 (C═N amide), 1530.23 (C═O), 1454.26 (C-C), 1363.25 (C-CH_3 aliphatic_), 1178.293, 813.56 (C-H), 672.07, 606.50 (C-Br), 534.18 (Ti-N), 461.86 (Ti-O), ESI Fig. S36†. Electrospray ionization ESI‐MS (m/z): Expected value 557.0317; Obtained value 557.4012 (M +), ESI Fig. S37†.

### [(acac)Ti(ph-4-H)L] (TiC7)

Yield 95%; Brown; m.*p* > 300 °C^1^H - NMR (DMSO-d_6_, 400 MHz, δ, ppm): 1.984 ( s 3 H, CH_3_), 2.030 ( s 3 H, CH_3_), 2.498 ( s 3 H, CH_3_), 5.796 ( s 1 H), 6.774–6.782 (q, 5 H, j = 9.8 Hz, CHAr), 7.142–7.181 (dd 4 H, j = 7.8hz, CHAr), 9.322 (s, 1 H, N = CH), ESI Fig. S38†^13^C NMR (DMSO‐d_6_, 100 MHz, δ, ppm): 20.88 (β‐CH_3_),24.58, 30.75, 58.02, 100.67, 111.03, 111.16, 114.02, 116.42, 116.88, 116.97, 117.93, 120.19, 120.73, 122.83, 124.12, 128.59, 129.42, 131.09, 124.20, 128.59, 129.42, 131.02, 138.72, 139.74, 150.33(C═N), 158.32,159.47,160.33, 190.38, 191.50, 203.61, ESI Fig. S39†. DEPT-135 NMR (DMSO‐d_6_, 100 MHz, δ, ppm): 58.02 C-H, ESI Fig. S40†. **Anal. Calcd** for C_25_H_22_BrNO_5_Ti (%): C, 55.17; H, 4.07; N, 2.57. Found (%): C, 54.89; H, 4.02; N, 2.62. FTIR (ATR, cm^− 1^): 2910.05 (CH), 1578.45 (C═N amide), 1529.27 (C═O), 1492.64 s (C-C), 1362.46 (C-CH_3 aliphatic_), 1289.18, 794.12 (C-H), 672.07, 606.50 (C-Br), 534.18 (Ti-N), 450.29 (Ti-O), ESI Fig. S41†. Electrospray ionization ESI‐MS (m/z): Expected value 545.0140; Obtained value 545.0219(M +), ESI Fig. S42†.

### Ti-2 L (TiC8)

Yield 95%; Brown; m.*p* > 300 °C^1^H - NMR (DMSO-d_6_, 400 MHz, δ, ppm): 1.929 ( s 3 H, CH_3_), 2.092 ( s 1 H, CH_3_), 2.274 ( s 2 H, CH_3_), 6.704–6.722 (d, 2 H, j = 3.6 Hz, CHAr), 6.876 (s 1 H, CHAr), 7.066–7.028 (d, 2 H, j = 4.4 Hz, CHAr), 7.183–7.203 (d, 2 H, j = 4.0 Hz, CHAr), 7.616–7.645 (dd, 2 H, j = 5.8 Hz, CHAr), 7.784–7.790 (d, 2 H, j = 1.2 Hz, CHAr), 10.234 (s, 1 H, N = CH), ESI Fig. S43†^13^C NMR (DMSO‐d_6_, 100 MHz, δ, ppm): 20.65 (β‐CH_3_),20.88, 110.95, 111.07, 113.92, 116.35, 116.78, 116.93, 119.64, 120.29, 120.29, 124.18, 131.35, 138.65, 138.65, 139.78, 150.86, 158.31, 158.69, 159.09, 159.46, 160.28, 172.43, 190.70, ESI Fig. S44†. **Anal. Calcd** for C_26_H_24_BrNO_5_Ti (%): C, 51.25; H, 3.07; N, 4.27. Found (%): C, 52.06; H, 4.33; N, 2.96. FTIR (ATR, cm^− 1^): 2911.07 (CH), 1574.32 (C═N amide), 1530.25 (C═O), 1435.01 (C-C), 1288.35 (C-CH_3 aliphatic_), 814.77 (C-H), 605.78 (C-Br), 532.25 (Ti-N), 458.97 (Ti-O), ESI Fig. S45†. Electrospray ionization ESI‐MS (m/z): Expected value 652.9350; Obtained value 653.4337(M +), ESI Fig. S46†.

### SCXRD of ligand

The crystal structure of **L1** (crystal code ASVS01) was determined using the Bruker D8 QUEST diffractometer taking molybdenum as the source of X-rays. Mo-Kα (0.71073 Å) radiation was used to determine the crystal properties and APEX4 software was used to collect the intensity data. The direct method (SHELXT program) was used to solve the structure. Full-matrix least-squares on F^2^ utilizing SHELXL-2019/1 optimized the SCXRD data (Sheldrick, 2019). Except for hydrogen, every atom was polished using anisotropic displacement parameters. ORTEP diagram for **L1** is displayed in ESI Fig. S5, S6† and analysis parameters are displayed in ESI Table S4-S10†. The crystal structure of L1 was identified as monoclinic with space group P1 and Z value 4. Unit cell parameters were found as: a = 11.6080 (4) Å, b = 11.8289 (5) Å, c = 9.8518(4) Å, α = 90^◦^, β = 114.7710(10)^◦^, γ = 90^◦^ and unit cell volume V = 1228.28(8) Å3. The ligand L1 has been given the CCDC deposition number 2,170,513.

### Liphophilicity

The traditional shake-flask method was used to calculate the log P values of Ti(IV) complexes as per the reported procedures^61–63^. An orbital shaker was used to shake a set quantity of TiC1-TiC8 suspended in water that had been pre-saturated with n-octanol for 48 h. In order to enable phase separation, the solution was centrifuged for ten minutes at 3000 rpm. Eventually, a UV-visible spectrophotometer was used to determine optical density and quantify the concentration of Ti(IV) complexes in the octanol solution and the aqueous solution independently.

### DNA binding studies

Binding relationships between newly synthesized Ti(IV) complexes and CT-DNA were ascertained using UV-Vis titration in a 5 mM phosphate buffer solution (PBS) of pH 7.4. The ratio of UV absorbance at each site was verified in the protein-free form of CT-DNA at 260 and 280 nm. The molar absorption coefficient (6600 m^− 1^) of CT-DNA observed at 260 nm was used to calculate the concentration of each nucleotide.

Metal complex stock solutions were prepared in DMSO and additional dilutions were made in 5 mM PBS buffer. In order to determine the CT-DNA reverse absorbance, binding experiments were performed using a fixed concentration (20 µM) of the complex and rising concentrations of CT-DNA (0–80 µM) in reference and sample solutions.

Using fluorescence spectroscopy, the competitive binding interaction of metal complexes with ethidium bromide-bound CT-DNA was investigated. The solutions of EtBr and CT-DNA of concentration 5 mM were made in PBS buffer for fluorescence titrations. The reference solutions were allowed to settle at room temperature for an hour and the reference solution of CT-DNA (200 µM) was coupled with ethidium bromide (50 µM) and kept at 4 °C and was allowed to attain room temperature for 10–20 min before the interaction studies were performed. The excitation and emission wavelengths were set at 460 nm and the reference solution of EtBr-DNA was titrated against Ti(IV) complexes with concentrations range 0–80 µM.

### Electrochemical studies

Utilizing a cyclic voltammeter (CV), the interaction between CT-DNA and the newly generated Ti(IV) complexes was studied. AgCl and carbon black served as the reference and counter electrodes, respectively and the platinum electrode served as the working electrode. A 0.1 M electrolytic solution of tetrabutylammonium hexafluorophosphate electrolyte solution was prepared in DMSO prior to the CV investigations. Ti(IV) complexes (1 × 10^− 3^ M) were then dissolved in the electrolyte to afford 1 mM concentration solutions and later their Cyclic voltammograms were recorded. To create a 5 mM DNA solution, CT-DNA was dissolved in 0.1 M PBS buffer with a pH adjustment of 7.4. Furthermore, the complexes were kept at constant concentrations and titrated by distinct, incremental additions of CT-DNA (0–50 µM) at a scan rate of 50 mV/s within the potential range of − 1.6 to 1.6 V versus Ag/AgCl.

### Gel electrophoresis

An agarose gel electrophoresis technique is an excellent technique for examining the degree of DNA binding with metallo-organic complexes. CT-DNA was dissolved in 0.1 M PBS buffer with a pH adjustment of 7.4 to prepare 5 mM DNA solution and in DMSO a stock solution containing 6 mM of each complex was kept ready before the start of the experiment. Regarding the gel electrophoresis, 20 µL of the Ti(IV) complexes were added to 20 µL of CT-DNA separately and the mixture was incubated for three h at 37 °C. PBS buffer with 1 mM EDTA, the prepared samples were electrophoresed on 1% agarose gel made ready in advance. Later, 20 µL incubated mixture of the Ti(IV) complexes and DNA was loaded separately in a 1:1 ratio onto the gel using 0.25% bromophenol blue dye. Until the bromophenol dye covered 50% of the gel, the electrophoresis was conducted in PBS buffer for an hour at 100 V. Following the whole movement of DNA, the electric current was cut off and *AXYGEN* digital camera was used to take pictures and visualize the gel under UV light using a transilluminator.

### BSA binding studies

After being made in 5 mM phosphate buffer (pH 7.4), the stock solution of BSA (5 mM) was kept at 2–6 ^◦^C. Later, employing UV–Vis absorption (200–500 nm) titration, the binding mechanism of Ti(IV) complexes with BSA was ascertained. The variations in the fluorescence intensity at a given excitation wavelength of 285 nm and emission value of 345 nm were used to track the quenching interactions of metal complexes with BSA. Throughout the studies, the same slit widths, emission and excitation scan speeds were used. A constant protein concentration was used to titrate against altering (0–80 µM) metal complexes’ concentrations. Similarly, the synchronous fluorescence spectra were obtained at the given excitation wavelengths 15 nm and 60 nm by titrating with different (0–80 µM) concentrations of metal complexes while keeping the concentration of BSA as constant.

The site marker study was carried out with the help of Fluorescence emission spectroscopy at an excitation wavelength 280 nm. In this experiment, two different site marker drugs Warfarin (WAR) and Ibuprofen (IBU) were used as specific site markers (probes) as they bind to the two most common drug-binding sites – Sudlow sites I and II. The site marker probes WAR and IBU weighing 10 mg individually were dissolved in PBS buffer separately. Afterwards, both the probe stock solutions were added with a known amount of BSA separately to make it 5 mM and kept at 2–6 ^◦^C. Later, the binary solution of the BSA along with the site marker was kept constant and was titrated against varied concentrations (0–80 µM) of metal complexes.

### **Theoretical calculations**

Density functional theory (DFT) calculations of newly synthesized Ti(IV) complexes were carried out using Gaussian 09 software. Optimized structures were determined employing Lee-Yang-Parr non-local correlation functional (B3LYP) and Becke’s three-parameter hybrid exchange method. In order to execute DFT calculations, the 6-31G** (d, p) basis set was used for all the lighter elements (C, H, N, O, and Cl) and the LanL2DZ effective core potential was incorporated for titanium metal centre. Molecular orbitals energies (E_HOMO_ and E_LUMO_), band gap (ΔE), essential charge transfer interface within the molecule, chemical potential (µ), global hardness (ɳ), global softness (S), global electrophilicity index (ω) and electronegativity (χ) were calculated using Eqs. (1–6) mentioned as follows:

1 $$\Delta \mathbf{E} = E_{\text{LUMO}} - E_{\text{HOMO}}$$

2 $$\:\varvec{\upmu\:}=\frac{\text{IP+EA}}{2}\:$$

3 $$\vec{\eta} = -\frac{E_{\text{LUMO}} - E_{\text{HOMO}}}{2}$$

4 $$\:\mathbf{S}=\frac{1}{\eta}\:$$

5 $$\omega = \frac{{\mu ^{2} }}{{2\eta ~}}$$

6 $$\varvec \chi = \varvec - \varvec \mu$$

### Molecular docking studies

Molecular Docking calculations were carried out employing default parameters on AutoDock Vina software and visualizations were executed using Discovery Studio. Molecular docking assesses the structural integrity of the conformational changes that occur from the ligand’s interaction with the receptor. Intermolecular energy difference between the bound and unbound states of the protein was explained by scoring functions. The intermolecular energy resulting from interactions between receptors and complexes was also computed. These studies are essential for predicting possible binding mechanisms of Ti(IV) derivatives with DNA (PDB ID: 1D28) and BSA (PDB ID: 4F5S) receptors. In order to perform Molecular Docking, it requires the input of the receptors and the Ti(IV) complexes in PDB format. Gaussian 16 (G 16) was used to draw the optimized structures of Ti(IV) derivatives in PDB file format and the protein data bank (http://www.rcsb.org/pdb ) was taken into account to download the crystal structures of DNA (PDB ID: 1BNA) and BSA (PDB ID: 4F5S). The Lamarckian genetic algorithm (LGA) was involved to investigate the energies of the flexible complexes and the likely bound conformations.

### DPPH assay

The DPPH assay is one characteristic method that is frequently used to investigate the antioxidant properties of the complexes. Stock solutions of synthesized Ti(IV) complexes were prepared in DMSO. Similarly, stock solution (0.05 mM) of DPPH was prepared in methanol. Different concentrations (9.5–150µM) of Ti(IV) complexes were prepared in DPPH stock solution followed by got loaded into 96 well plates and allowed to rest for 30 min in the closed condition at 298 K. Subsequently, using methanol as a blank and a methanolic solution of DPPH serving as a control, the absorbance at 517 nm was measured for each solution loaded in 96 well plates using Bio-Rad xMARKTM Microplate Spectrophotometer. A lower absorbance of the reaction mixture signifies an increase in free radical scavenging activity. The percentage of DPPH free radical scavenging activity was estimated using Eq. (7).

7 $$\:\mathbf{\%}\:\mathbf{I}\mathbf{n}\mathbf{h}\mathbf{i}\mathbf{b}\mathbf{i}\mathbf{t}\mathbf{i}\mathbf{o}\mathbf{n}=\frac{\mathbf{A}\mathbf{c}-\mathbf{A}\mathbf{s}}{\mathbf{A}\mathbf{c}}\times\:100$$

where Ac and As are the absorbance of the control and the mixture. IC_50_ value, which represents the sample concentration that causes 50% inhibition, was also determined. The tests were done in triplicate with ascorbic acid as a standard.

### Cytotoxic studies

Standard (4,5-dimethylthiazol-2-yl)-2,5-diphenyltetrazolium bromide (MTT) test procedure was followed to complete the in-vitro cytotoxic investigation. Before being diluted with DMEM medium, the newly synthesized Ti(IV) complexes were first dissolved in 0.1% DMSO. In this instance, two cancer cell lines human epithelioid cervix carcinoma (HeLa) and human breast cancer cell line (MCF-7) cells were obtained from the National Centre for Cell Science (NCCS) Pune and were used in the experiments. In 96-well plates, 1 × 10^4^ cells were cultivated in 100 µL of growth media i.e. Dulbecco’s modified Eagle’s medium (DMEM) with 10% fetal bovine serum (FBS) and the plates were then incubated at 37 °C with a 5% CO_2_ atmosphere overnight. After incubation, the cells were treated with varying concentrations of the complexes in a volume of 100 µL per well (9.5–300 µM) for the HeLa cell line after 48 h of seeding and incubation of cells.

Cisplatin was used as the control in this experiment. Ti(IV) complexes were added to the media containing 0.1% DMSO also occupied by the cells in the control wells. After 48 h of incubation at 37 °C for HeLa cells, the media was deemed and the cell cultures were once more incubated for 5 h at 37 °C with the addition of 100 µL of the MTT reagent (1 mg mL^− 1^). Subsequently, the suspension was left on a micro vibrator for ten minutes, absorbance at λ = 570 nm was measured using Bio-Rad xMARKTM Microplate Spectrophotometer plate reader.

Approximately, five thousand MCF7 cells were cultured in each well of 96-well plates. After allowing the cells to grow for a full day, Ti(IV) complexes were added to the cells at varied concentrations (9.5–300 µM). The plate was incubated at 37 °C for 4 h, later, 10 µL of 3-(4,4-dimethylthiazol-2-yl)-2,5-diphenyltetrazolium bromide (MTT) solution (5 mg mL^− 1^ in PBS, final concentration 0.5 mg mL^− 1^) was added to each well after 48 h.

At 570 nm wavelength, absorbance was measured using Bio-Rad xMARKTM Microplate Spectrophotometer plate scanner. Additionally, the experiment was run in triplicate for both the cell lines. The information was expressed as a cell viability percentage that can be calculated using following Eq. (8):

8 $$\:\mathbf{C}\mathbf{e}\mathbf{l}\mathbf{l}\:\mathbf{V}\mathbf{i}\mathbf{a}\mathbf{b}\mathbf{i}\mathbf{l}\mathbf{i}\mathbf{t}\mathbf{y}\:\mathbf{\%}=\frac{({\varvec{A}}_{\varvec{s}\varvec{a}\varvec{m}\varvec{p}\varvec{l}\varvec{e}}-{\varvec{A}}_{\varvec{m}\varvec{e}\varvec{d}\varvec{i}\varvec{u}\varvec{m}})}{({\varvec{A}}_{\varvec{c}\varvec{e}\varvec{l}\varvec{l}\:\varvec{c}\varvec{o}\varvec{n}\varvec{t}\varvec{r}\varvec{o}\varvec{l}}-{\varvec{A}}_{\varvec{m}\varvec{e}\varvec{d}\varvec{i}\varvec{u}\varvec{m}})}\times\:100$$

Where A_sample_, A_medium_ and A_blank_ are the absorbance values of the sample, medium and control.

### AO/EB staining

The changes in the organization in Hela cells after treatment with IC_**50**_ concentrations of the complexes **TiC2** and **TiC8** were investigated using acridine orange (AO) and ethidium bromide (EB) assay. HeLa cells were seeded in 6-well plates at a density of 5 × 10^5^. They were grown in a humidified CO_2_ incubator at 37 °C and kept for 24 h until they reached 70–80% confluency. Later the cells were treated with IC_50_ concentration of complexes and incubated for 48 h. Finally, the cells were allowed to recover for 1 h, each well culture media was removed and the cells were carefully washed twice at room temperature using PBS and stained with an AO and EB stain (1:1, 10 µM) for 15 min separately and observed under Olympus Confocal Laser Scanning Microscope- Fluoview Fv3000 to capture their respective images.

### Cell cycle analysis with flow cytometry and Propidium iodide

Flow cytometry is one of the most extensively used and widely accepted laser-based biophysical techniques that are employed in cell counting, cell sorting, apoptotic phase analysis, cell cycle phase distribution and biomarker detection. Cell cycle analysis through flow cytometry (FACS) with propidium iodide (PI) staining was performed to ensure cell cycle arrest or alteration in cell cycle phases in HeLa-treated cells. Briefly, 4 × 10^5^ cells were seeded in 6-well culture plates with complete DMEM and left for 24 h in a CO_2_ incubator to adhere properly. After treatment with IC_50_ concentrations of the complexes in the medium, the cells were incubated for 24 h at 37 °C in a CO_2_ incubator supplemented with 5% CO_2_. Thus, after incubation, the cells were collected using cold PBS containing 1 mM EDTA and a gentle vortex was used to prepare a single-cell suspension. Cells were fixed with 70% ethanol and left to stand at -20 °C for the entire night. Moreover, ice-cold PBS was used to wash the cells before they were stained with a PI solution (1 mg mL^− 1^) and allowed to incubate for 30 min at 37 °C. Additionally, the labelled cells were subjected for analysis of cell cycle distribution using a FACS scan and the Becton Dickinson cell search program.

### Intracellular reactive oxygen species (ROS) detection

Using an oxidation-sensitive fluorescent probe, 2′,7′-dichlorofluorescein diacetate (DCFHDA), 2 × 10^5^ cells per well were seeded and treated with IC_**50**_ concentrations of the complexes. The cells were then incubated at 37 °C with 5% CO_2_ for 48 h to measure intracellular ROS. The cells’ internal esterase degraded DCFH-DA, converting it into a non-fluorescent DCF-H product. Furthermore, when intracellular ROS are present, this DCF-H is oxidized and transforms into an incredibly luminous DCF product that signals the presence of ROS. Olympus Confocal Laser Scanning Microscope Fluoview Fv3000 captured phase-contrast pictures and green fluorescence for the qualitative determination of ROS.

## Results and discussion

### Synthesis and formation of complexes

**Fig. 1:**
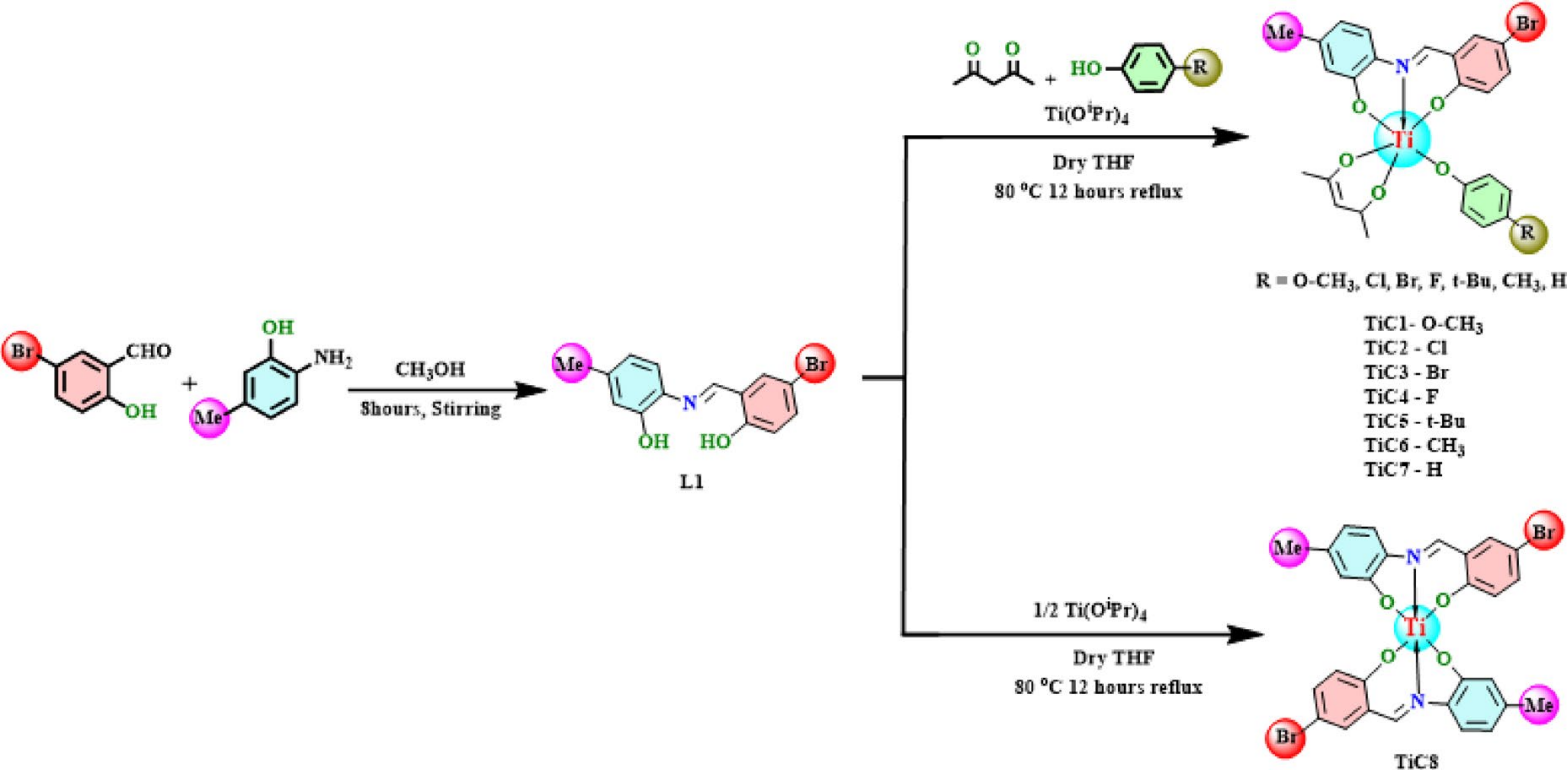
Synthesis of ligand L1 and complexes TiC1-TiC8.

The ligand (L1) 4-bromo-2-{[(2-hydroxy-4-methylphenyl)iminio]methyl}phenol was synthesized by reacting methanolic solutions of 5-bromosalicylaldehyde and 2-Amino-5-methyl phenol in equimolar ratios (Fig. 1). The desired ligand was obtained in good yields and was characterized by different spectroscopic techniques (data in ESI Fig. S1-S6†) and the molecular structure of the ligand was confirmed by single-crystal X-ray diffraction ESI Fig. S4-S6 and Table S4-S10†.

Titanium isopropoxide was used as the starting material in synthesizing new Ti(IV) complexes **(TiC1-TiC8)** along with the stoichiometric quantity of ligand [**L1**], acetyl acetone and phenol derivatives were refluxed for 8 h at 80 °C (Fig. 1). Later, all these new complexes were characterized using spectral tools.

**TiC1-TiC8** were ascertained while comparing the NMR spectra of ligand **(L1)** as well as of the synthesized complexes. The ^1^H spectrum of **(L1)** ESI (Fig. S1†) displayed peaks at 14.001 ppm and 9.742 ppm assigned to the hydroxyl –OH groups which were found to be absent in the ^1^H NMR spectra of the complexes (ESI Fig. S7, S12, S17, S22, S28, S33, S38 and S43†), incriminating the coordination of the hydroxyl group with the titanium centre *via* deprotonation as providing evidence of the complexation. Further, the imine protons of **TiC1–TiC8** were observed as singlet peaks in the range of 10.01–10.30 ppm and the rest of the aromatic protons were found as expected (6.50–7.80 ppm) to authenticate the formation of new complexes and the de-shielding was observed in methyl protons that appeared at 2.068ppm. Depending on the substituents of the phenol ligand derivatives, the de-shielding of the azomethine proton (CH = N) was found to be higher in case of TiC1, TiC4, TiC5 and TiC8, whereas only slight de-shielding was observed in the case of TiC2, TiC3, TiC6 and TiC7 complexes. Another important feature observed in the IR spectra of complexes **(TiC1-TiC8)** was at an appreciable frequency of 1598 cm^− 1^ which depicted the acetylacetonate peak for C-H absorption. Later on complexation, it was observed in complexes that the acetylacetonate peak shifted to 1604 cm^− 1^ exhibiting a keto-enol form of tautomerism involved in the complexation with Ti(IV) metal ion and the disappearance of OH peak at 3030.17 cm^− 1^ exhibited a very strong evidence of the complex formation and the emergence of imine C = N peak at 2911.28 cm^− 1^ (ESI Fig. S3, S10, S15, S20, S26, S31, S36, S41 and S45†). In addition, the generation of corresponding new strong peaks ranging from 430 to 495 cm^− 1^ in all the complexes revealed Ti–O bond formation in respective derivatives. Significant stretching frequencies of C-N and phenolic C–O peaks from the ligand moieties were visible at around 1605 cm^− 1^ and 1263 to 1290 cm^− 1^, respectively. Subsequently, Ti–N bond and C-halogen stretching frequencies were observed at 532–540 cm^− 1^ and 735–743 cm^− 1^, respectively.

### UV-visible and fluorescence study

UV-visible and fluorescence spectrophotometers were employed for the absorption and emission assessments using DMSO and water (1:9) medium at 298 K to study the photophysical and luminescence properties of the newly synthesized titanium(IV) complexes. UV-Vis spectra of **TiC1-TiC8** exhibited unlike possible transitions between 250 and 500 nm, the absorption spectra and photophysical data of the complexes are displayed in ESI Fig. S47† and (Table 1). The appearance of bands at 260 nm and 300 nm corresponds to the charge transfer that occurs either from the highest occupied molecular orbital (HOMO) to the lowest unoccupied molecular orbital (LUMO) or from the ligands’ π bonding molecular orbitals to the π* antibonding molecular orbitals assigned as n- p* and p - p* transitions.

The bands at 330 nm to 400 nm correspond to ligand-metal charge transfer (LMCT). The highest absorption peak was observed in **TiC1** and **TiC2** at 290 nm and 275 nm. It was observed in these complexes that they underwent a blue shift. Of course, these Ti(IV) complexes have no possibility of d-d transitions, so the overriding changes could be attributed to the charge transfer transitions.

Equation (9) was applied to the new Ti(IV) complex emission spectra to determine the luminescence quantum yields (φf) in 10% DMSO (aq).

9 $$\varphi = \varphi {\text{R}} \times \frac{{{\mathbf{I}}_{{\mathbf{s}}} }}{{{\mathbf{I}}_{{\mathbf{R}}} }} \times \frac{{{\mathbf{OD}}_{{\mathbf{R}}} }}{{{\mathbf{OD}}_{{\mathbf{S}}} }} \times \frac{{\eta _{{\mathbf{S}}} ~}}{{\eta _{{\mathbf{R}}} }}$$

Notations involved herein are as φ = quantum yield, I = peak area, OD = absorbance at λ_max_ of sample(s) and reference(R), η = refractive index of solvent (s) and reference (R).

As shown in ESI Fig. S48† and Table 1, essentially identical emission bands were found at 343, 347, 350, 340, 337, 390, 390 and 350 nm. Following the excitation of the complexes at 350 nmthe emission spectra persisted until 500 nm. For all Ti(IV) complexes at the excitation peak of 350 nm, values for Stoke’s shift and luminescence quantum yield (φ_f_) were computed in (Table 1). **TiC1** displayed the highest optical density and extension coefficient, while **TiC8** showed the highest luminescence quantum yield (φ_f_ = 0.0464) among the complexes.

**Table 1:** Photophysical characterization of all **Ti(IV)** complexes; ^a^Absorption maxima; ^b^Wavelength of emission spectra; ^c^Stokes shift; ^d^Optical density; ^e^Extinction coefficient; ^f^Quantum yield; ^g^Partition coefficient of n-Octanol/water; ^h^Conductance in DMSO and DMSO–aqueous (1: 9) medium.

Complexes	λ _max_^a^ (nm)		λ _f_^b^ (nm)	Stoke’s shift^c^	OD^d^	ε^e^ (M^− 1^cm^− 1^)	(φ_f_)^f^	log *P*_o/w_^g^	ΛM^h^ (S cm^2^mol^−1^)	
	π − π*	LMCT							DMSO	10%DMSO
TiC1	285	343	326	17	1.9	54,000	0.0168	0.12	30	110
TiC2	265	347	314	33	1.6	54,000	0.0190	0.03	28	115
TiC3	280	350	322	28	1.5	28,500	0.0206	0.16	56	136
TiC4	282	340	320	20	1.0	15,000	0.0317	0.27	49	125
TiC5	277	337	375	38	1.1	22,000	0.0338	0.49	62	156
TiC6	272	390	320	70	1.6	29,500	0.0173	0.20	36	118
TiC7	280	390	333	57	1.5	30,500	0.0191	0.17	54	148
TiC8	263	350	320	30	1.3	60,000	0.0464	0.07	38	122

### Solubility

The capability of metal complexes to prevent the growth of tumors is highly dependent on the equilibrium between hydrophilicity and lipophilicity. The prepared complexes were nearly soluble in benzene, DMF and THF but fairly soluble in DMSO while rationally soluble in H_2_O, MeOH, EtOH, and MeCN. Eventually, it is important to note that these **TiC1-TiC8** were found soluble (1 mg/mL) in DMSO and 10% DMSO(aq) at room temperature.

### Conductivity studies

Molar conductance values of **TiC1-TiC8** in DMSO are displayed in (Table 1). Conductance experiments of these complexes were performed in neat DMSO as well as in 10% DMSO aqueous medium. A remarkable increase in molar conductance of the complexes in 10% aqueous DMSO suggested their electrolytic nature and **TiC5**,** TiC7** and **TiC3** exhibited greater conductance values out of all eight complexes (Table 1). Thus, the complexes’ acquired conductivity was related to the Ti-O and Ti-N bonds dissociation in the tested system and the ionization of complexes in an aqueous medium was validated, ensuring considerable binding characteristics of the complexes with biomolecules.

### Stability

Time-dependent UV-visible spectral stability studies of the new Ti(IV) complexes using two distinct solvent systems; 1:9 DMSO: H_2_O and GSH media were recorded for over 72 h. **TiC1-TiC8** exhibited a hypochromic shift in the π–π* and MLCT regions for the first 24 h, and kept assessing stabilization investigation for up to 72 h as visible in ESI Fig. S49†. However, the complexes **TiC1**,** TiC6**, and **TiC7** exhibited a small amount of hyperchromism in the LMCT area. **TiC3** and **TiC5** did not realize change significantly and remained steady for 48 h until displaying hypochromism in the π–π* area for 72 h. Interestingly, even after 72 h, **TiC2** and **TiC4** complexes stayed stable.

It is commonly recognized that when glutathione S-transferase (GST) is present in cells, glutathione (GSH) plays a critical detoxifying role. Further, to observe the GSH activity on these Ti(IV) derivatives, a time-dependent UV-Vis spectroscopy stability investigations were performed in the presence of excess glutathione. ESI Fig. S50† represents that there were only slight variations in absorbance over time, indicated that complexes were not being deactivated by GSH, whereas, in the GSH medium all the complexes persisted as such for 72 h.

### Partition coefficient determination (lipophilicity)

Potential of the complexes to permeate cell membranes is evaluated by analyzing their lipophilicity using the partition coefficient (log P) values. It is determined by the distribution and solubility of the complexes in a mixture of octanol and water using the shake flask method^64^. Computed log P values of **TiC1-TiC8** are depicted in Table 1, reflected that the complexes differ in lipophilicity, probably because of various substituents in the co-ligands. **TiC5** and **TiC4** possess tertiary butyl and fluoro substituted moieties, exhibited greater lipophilicity than the mate complexes (ESI Fig. S51†), it proved that the substituents on the phenyl ring boosted the lipophilicity. The observed log P values for the complexes are consistent with the reported titanium complexes.

### DNA binding studies

#### UV-vis absorption titrations

The technique of using electronic absorption spectroscopy appeared to be efficacious in investigating the DNA binding mechanism with the metal complexes.

In order to determine the binding mechanism and affinity of the newly synthesized Ti(IV) complexes associated with calf thymus DNA and to demonstrate their potential towards various cancer cells and their DNA binding capability, UV-Visible spectrophotometric DNA titration was performed. DNA is the main pharmacological target for a wide range of anticancer agents. Hence, an appropriate investigation into the DNA binding profile of these metal complexes turns crucial in designing and formulation of metallo-drugs^65,66^.

**Fig. 2:**
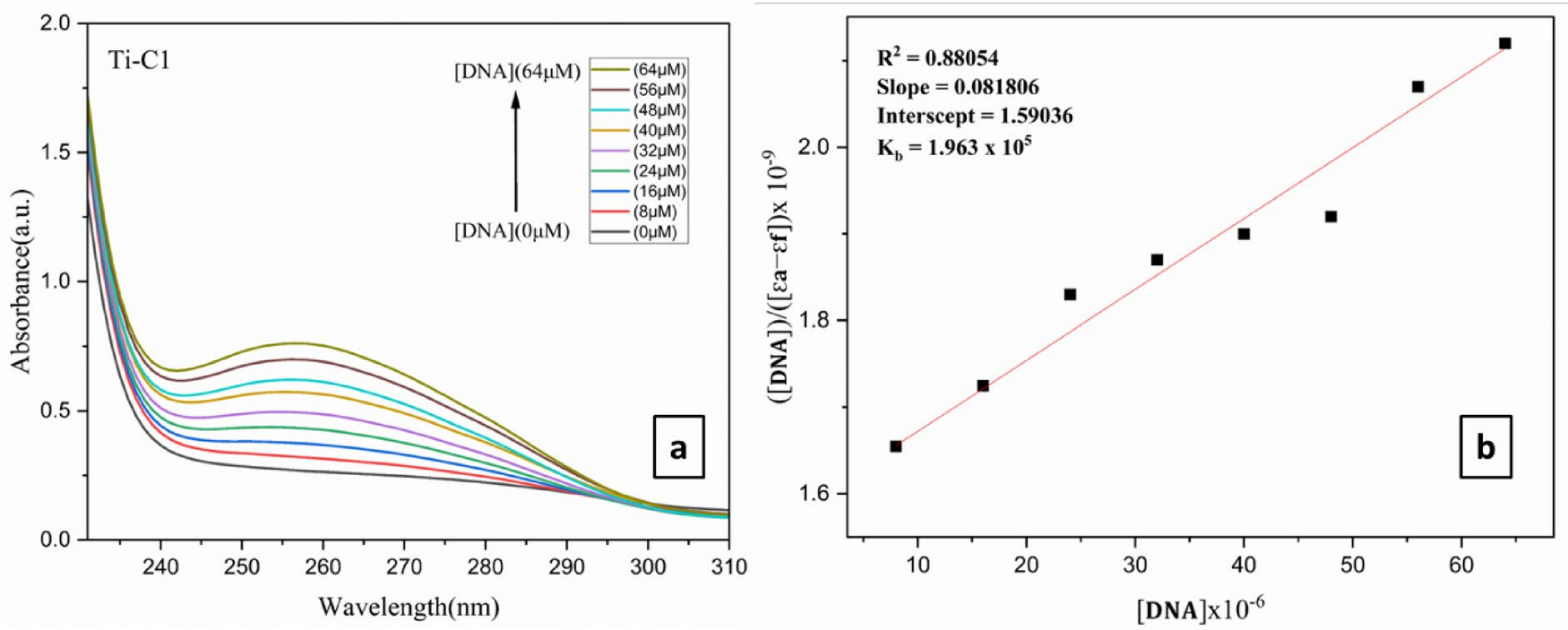
(**a**) UV absorption titration for **TiC1** with increasing CT-DNA (0–64 µM) concentration in DMSO; (**b**) linear plot of **TiC1**.

Purine (adenine and guanine) and pyrimidine (cytosine and thymine) DNA base pairs are known as referents, and they act as catalysts for electronic changes. The newly synthesized complexes of titanium(IV) were subjected for DNA binding studies using UV-visible titration, and the resulting λ_max_ values were determined. As the concentration of DNA was increased by keeping Ti(IV) complexes as constant in PBS buffer, these data reflected a hyperchromic shift in the absorbance intensity (ESI Fig. 52† and Table 2). **TiC1-TiC8** exhibited distinct absorbance bands at 255–260 nm due to intraligand p / p* transitions and LMCT. During the addition of DNA (0 to 64 µM) to Ti(IV) derivatives, hyperchromic shift (255–260 nm) was observed (ESI Fig. S52(a, c, e, g, i, k, m and o)†) and **TiC1** in Fig. 2, that indicates the existence of groove binding between the DNA and complexes.

The hyperchromic shift indicates that the modes of binding for the metal complex and DNA may be covalent and noncovalent. Later, the binding parameters including binding constant (K_b_) and binding sites were calculated for the above-said complexes by means of Eq. (10) as given below:

10 $$\frac{{\left[ {{\mathbf{DNA}}} \right]}}{{\left[ {\varepsilon _{{\mathbf{a}}} - \varepsilon _{{\mathbf{f}}} } \right]}} = \frac{{\left[ {{\mathbf{DNA}}} \right]}}{{\left[ {\varepsilon _{{\mathbf{b}}} - \varepsilon _{{\mathbf{f}}} } \right]}} + \frac{1}{{{\mathbf{Kb}}\left[ {\varepsilon _{{\mathbf{a}}} - \varepsilon _{{\mathbf{f}}} } \right]}}$$

where ε_a_ is the apparent extinction coefficient of the complex, ε_f_ is the extinction coefficient of the complex in its free form, ε_b_ is the extinction coefficient of the complex when completely bound to DNA and [DNA] is the concentration of DNA in the base pairs. Linear plots of [DNA]/(ε_a_ - ε_f_) vs. [DNA] were obtained from the resultant data.

Electrostatic, covalent and noncovalent interactions (intercalation and groove binding) are widely recognized methods to demonstrate how metal complexes bind with DNA. Calculating the intrinsic binding constant (K_b_), it was found that Ti(IV) complexes displayed groove binding due to p-p* stacking interactions (Fig. 2, ESI Fig. S52(b, d, f, h, j, l, n and p)† and Table 2). **TiC2** and **TiC4** exhibited higher K_b_ values as 5.54 × 10^5^ and 5.07 × 10^5^ when compared among all the Ti(IV) complexes as displayed in (Table 2).

#### Competitive DNA binding: EtBr fluorescence quenching studies

Fluorescence spectra measurements were performed on CT-DNA by altering the concentration of the complexes to elucidate their binding mechanism. The fluorescence emission intensity of Ethidium Bromide attached to DNA as a probe is used to assess the binding of the Ti(IV) complexes with CT-DNA at ambient temperature in aqueous solution as it does not exhibit any luminescence^67,68^. Because of the strong contact between the neighbouring DNA base pairs and EtBr a fluorophore, which could be mitigated by the addition of another molecule, where EtBr exhibits powerful fluorescent properties in the presence of DNA. The study examined the proportional binding of every titanium complex to CT-DNA using a solution of EtBr-bound CT-DNA in a 5 mM PBS (pH = 7.4).

**Fig. 3:**
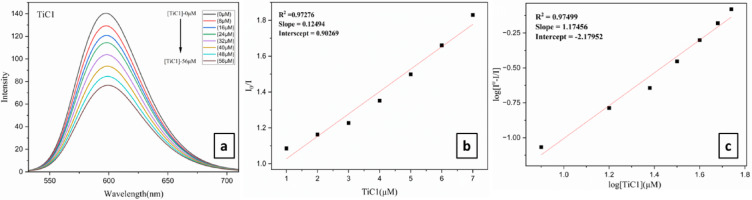
(**a**) Fluorometric titrations of CT-DNA 5 mM concentration in DMSO with **TiC1** (0–56 µM); **(b)** Stern Volmer plot; **(c)** Modified Stern-Volmer/ Scatchard plot for **TiC1**.

In order to explore the binding pattern of DNA with metal complexes, a titration was carried out at increased concentrations (0–80 µM) of **TiC1-TiC8** while affixing the concentration of EtBr-CT-DNA, where a decrease in fluorescence intensity was detected for EtBr bound 5 mM CT-DNA. The decreased fluorescence intensity might be ascribed to the displacement of the EtBr molecule from the CT-DNA. **TiC1-TiC8**, on titration with EtBr-CT-DNA displayed a decrease in fluorescence intensity during competitive binding studies (Fig. 3a, ESI Fig. S53(a, d, g, j, m, p, s and v) † and Table 2) and the excitation and emission wavelengths were observed at 600 nm. These complexes drove EtBr out of the CT-DNA grooves and bound themselves to the CT-DNA base pairs.

Magnitude of the binding strength determination quantitatively for new Ti(IV) complexes with CT-DNA, the quenching ability has been then analyzed by the Stern–Volmer equation and K_SV_ values are provided in Table 2, where low association between Kb and K_SV_ values was observed, possibly due to the dissimilar sensitivity of the two techniques used and Eq. (11) was taken in account to calculate the Stern-Volmer quenching constant K_SV_.

11 $${\mathbf{I}}_{{\mathbf{0}}} /{\mathbf{I}} = 1 + {\mathbf{Ksv}}\left[ {\mathbf{Q}} \right]$$

Here ‘I’ is the fluorescence intensity of the DNA-EB adduct in the presence of the complex (quencher) and I_0_ is the fluorescence intensity of the adduct in its absence. K_SV_ is the Stern-Volmer quenching constant and [Q] is the quencher concentration (Fig. 3b, ESI Fig. S53(b, e, h, k, n, q, t and w) †).

Furthermore, binding constant K_app_ was assessed and the values obtained using equation (12) for **TiC1-TiC8**.

12 $${\text{K}}_{{{\text{app}}}} \times \left[ {{\text{Complex}}} \right]_{{50}} = {\text{k}}_{{{\text{EtBr}}}} ~ \times ~\left[ {{\text{EtBr}}} \right]$$

Here [EtBr] is the concentration of EtBr = 10 µM, [Complex]_50_ denotes the complex concentration at which the fluorescence intensity of the DNA-EB adduct is decreased to 50%, and K_EtBr_ (1.0 × 10^7^ M^− 1^) is the DNA binding constant of EtBr.

Scatchard Eq. (13) was used to obtain Scatchard plots (Fig. 3c, ESI Fig. S53(c, f, i, l, o, r, u and x) †). Further, computations were performed to ascertain the binding sites (n) and the equilibrium binding constant K, where I_0_ represents the fluorescence intensity of CT-DNA + EtBr in the absence of complex and I stands for the fluorescence intensities of CT-DNA + EtBr in the presence of a complex concentration [Q].

13 $${\mathbf{Log}}{\text{ }}\left( {{\mathbf{I}}_{{\mathbf{0}}} - {\text{ }}{\mathbf{I}}/{\mathbf{I}}} \right) = {\mathbf{logK}} + {\mathbf{n}}~{\mathbf{log}}\left[ {\mathbf{Q}} \right]$$

From the results, it is observed that Ti(IV) complexes bind with CT-DNA *via* groove binding mode, as observed by the substantial quenching in fluorescence intensity of all metal complexes, among them it is observed that **TiC2**, **TiC4** and **TiC6** exhibited higher intrinsic DNA binding constant values (K_b_), **TiC7**, **TiC6**, **TiC3** and **TiC1** displayed higher K_**SV**_, Stern − Volmer quenching constant, **TiC1**, **TiC2** and **TiC6** reflected higher K_app_, apparent DNA binding constant, **TiC2**, **TiC1** and **TiC5** appeared with higher n, i.e. number of binding sites when compared with other complexes as illustrated in (Table 2).

**Table 2:** Binding factors for the CT-DNA interaction with all the Ti(IV) complexes; ^a^K_b_, intrinsic DNA binding constant; ^b^K_SV_, stern − volmer quenching constant; ^c^K_app_, apparent DNA binding constant; ^d^n, number of binding sites.

Complex	λ_max_ (nm)	Change in absorbance intensity	K_b_^a^ (× 10^5^ M^− 1^)	K_SV_^b^ (× 10^6^ M^− 1^)	K_app_^c^ (× 10^6^ M^− 1^)	*n* ^d^
TiC1	258	Hyperchromism (30.48%)	1.96	0.124	2.87	1.17
TiC2	260	Hyperchromism (18.46%)	5.54	0.104	2.42	1.26
TiC3	260	Hyperchromism (28.69%)	2.09	0.131	2.27	1.04
TiC4	259	Hyperchromism (29.41%)	5.07	0.090	1.51	0.89
TiC5	260	Hyperchromism (13.33%)	2.60	0.092	1.66	1.16
TiC6	258	Hyperchromism (20.36%)	3.17	0.138	2.42	0.98
TiC7	260	Hyperchromism (16.45%)	1.73	0.140	2.27	1.02
TiC8	262	Hyperchromism (32.56%)	1.27	0.067	2.27	1.92

#### Viscosity studies

The prominent approach to identify the binding mode of CT-DNA with metal complexes is viscosity studies that measures the change in CT-DNA chain length after the interaction with the metal complex. According to certain research findings, if compounds are inserted between DNA base pairs, the viscosity of the CT-DNA solution will increase. This finding suggests that interacting with ligands may encourage base pair separation, which will lengthen the CT-DNA helix. In the presence of a groove or other nonclassical interaction, double helix seems to be bent or twisted, affecting in shortening of the CT-DNA length and consequently reducing its viscosity^69^.

The current work elaborates on the attempt to determine the binding mechanism between the Ti(IV) complexes and CT-DNA by measuring the viscosity in response to an increase in complex concentration. The plots of (η/η^0^)^1/3^ vs. r (the molar ratio of complex vs. CT-DNA) are displayed in ESI Fig. S54† that reveals a modest decrease in CT-DNA viscosity after binding with Ti(IV) complexe(s). Results obtained herein suggested that interaction occurred through groove binding mode between CT-DNA and Ti(IV) derivatives. Further, the complexes have bent the unique size of DNA by inserting themselves in between the CT-DNA grooves. Among all the complexes synthesized, viscosity experiment adequately demonstrated how **TiC1-TiC8** interact with DNA *via* strong groove binding while favoring DNA binding mode.

#### DNA binding studies by Cyclic voltammetry

A highly sensitive approach that enhances other common methods such as fluorescence and absorption investigations in the use of electroanalytical measures as probe for the interactions between nucleic acids and metal complexes. The electrochemical method is an alternate method to determine band overlap and weak absorption bands (d-d electronic transition). Although metallo-organic complexes have been studied using cyclic voltammetry because of their accessible redox states. Some reports have expressed the electrochemical profiles of the DNA that are attributed to the redox behaviour of purine and pyrimidine bases as well^70–73^.

**Fig. 4:**
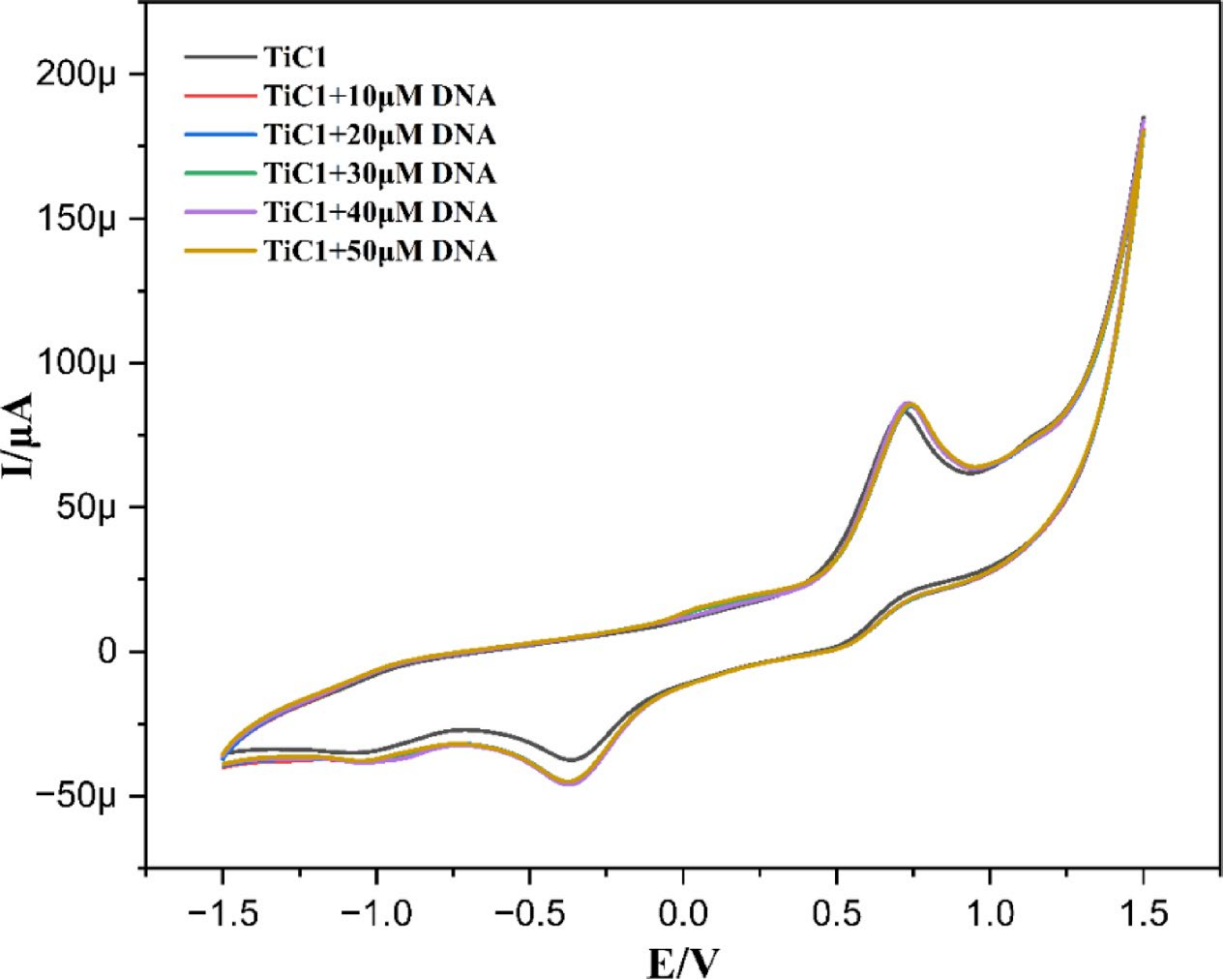
Cyclic voltammogram of **TiC1** in the absence and presence of CT-DNA.

Cyclic voltammetry is a flexible and popular technique that assesses the binding mode of metallo-organic complexes with CT-DNA. Thus, direct nucleobase oxidation by redox active metal complex or indirect detection using these DNA-binding complexes could be investigated. DNA interaction with the complexes might cause the peak potential and current to alter while sometimes reverse the scan, so the species in the backward scan are also important in revealing the binding mode.The cyclic voltammogram of the Ti(IV) complexes in the absence and in presence of the CT-DNA have been recorded in 0.1 M electrolytic solution of tetrabutylammonium hexafluorophosphate with a scan rate of 50 mVs − 1 over a potential range from − 1.5 to 1.5 V at room temperature. The characteristic stripping peak for the complexes appeared at different potentials displaying both cathodic and anodic peaks as displayed in Table S1. It was evident from Fig. 4 that peaks appearing at + 0.058 and + 0.75 V resembled with the oxidation peak of TiC1 at the electrode surface coupled with its reduction peak at -0.32 and − 1.02 V, respectively.

By titrating CT-DNA (0–80 µM) with the 0.1 M electrolytic solution of **TiC1-TiC8** (1 × 10^− 3^ M), it was observed from Fig. 4 and ESI Fig. S55† that there was a slight shift towards the cathodic and anodic current and also it was closely observed that there was a deviation of the peak towards positive potential as the indication of groove binding. Likewise, all Ti(IV) complexes exhibited similar sorts of oxidation and reduction peaks associated with the movement of current towards positive potential as observed and highlighted in ESI Table S1 and Fig. S55†, establishing groove binding.

#### DNA cleavage by gel electrophoresis

**Fig. 5:**
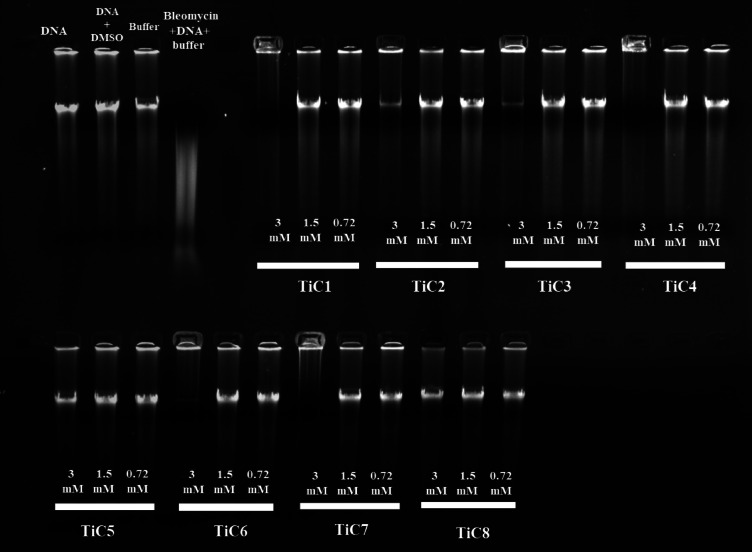
Agarose gel electrophoresis of CT-DNA with **TiC1-TiC8 (1)** CT-DNA in PBS buffer; **(2)** CT-DNA in PBS buffer + DMSO; **(3)** PBS; **(4)** CT-DNA in PBS buffer + bleomycin; **(5)** Lanes 5–28 CT-DNA+ (3, 1.5 and 0.75 mM) **TiC1-TiC8**.

Agarose gel electrophoresis is a straightforward and incredibly efficient technique for separating, detecting and purifying nucleic acid fragments in DNA. A gel electrophoresis experiment was conducted to determine whether the produced complexes interacted with CT-DNA^74^. Samples of 1% agarose gel containing DNA-binding dye was loaded with ladder, CT-DNA (20 µL) and Ti(IV) complexes (0.72-3 mM), respectively, and these specimens were incubated for three h. An electric current of 100 V was administered for half an hour. Under UV light, it was evident that all complexes interacted with CT-DNA and the highest concentration samples of **TiC1**,** TiC2**,** TiC3**,** TiC4**,** TiC6** and **TiC7** were capable of cleaving the CT-DNA compared to other concentrations of the complexes, whereas, **TiC5** and **TiC8** could not cleave even in the highest concentration (3mM) as making it visible in (Fig. 5).

### BSA binding studies through fluorescence quenching

The extent to which a drug binds with blood plasma proteins affects its effectiveness. Many endogenous and exogenous materials involved in cell metabolism are excited and administered with the aid of serum albumin, a notable component of plasma proteins, to determine the ability of the complexes as well as to bind and examine the structural alterations in the secondary structure of BSA. BSA, a structural equivalent of HSA is mostly found in cellular environments, aids in drug dispersion^75,76^. The fluorescence titration method is the most appropriate method used in the BSA binding experiment to investigate the ability of Ti(IV) complexes (1 × 10^− 5^ M)with their gradual addition (0 to 56 µM) into BSA (3 × 10^− 6^ M) to determine binding efficacy.

**Fig. 6:**
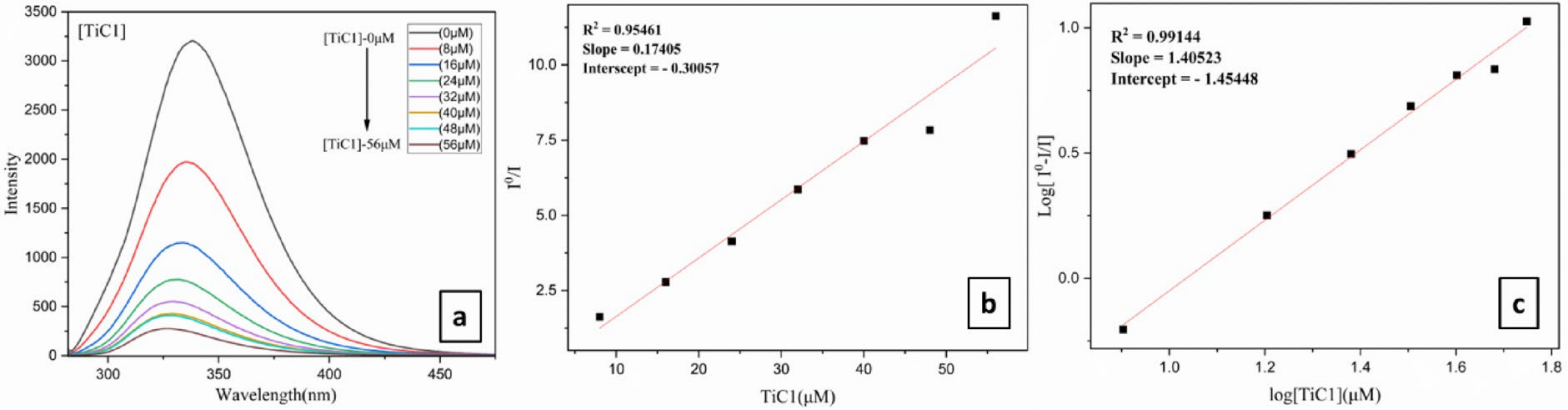
(**a**) Fluorometric titrations of BSA (1 × 10^− 5^ M) with **TiC1** of concentration (0–56 µM); (**b**) Stern-Volmer plot and; (**c**) Modified Stern-Volmer plot for **TiC1**.

Proteins bound with metal complexes suppress the intrinsic fluorescence of phenylalanine, tyrosine and tryptophan in either a static or dynamic fashion. The BSA molecule in this study displayed strong fluorescence at 335 nm. The plot illustrates how the fluorescence of BSA reflected a clear decline (ca. 70–80%) when the concentration of the relevant metal complexes was increased (Fig. 6a and ESI Fig. S56 (a, d, g, j, m, p, s and v)†. K_q_ and K_BSA_ were evaluated using Stern–Volmer Eq. (14) where the K_q_ values of the newly synthesized complexes suggested static quenching mode and are given in Fig. 6b and ESI Fig. S56 (b, e, h, k, n, q, t and w)†. Number of binding sites (n) and binding constants (K_b_) were calculated from the Scatchard Eq. (15) and Scatchard plots are displayed in Fig. 6c and ESI Fig. S56 (c, f, i, l, o, r, u and x)†.

14 $${\mathbf{I}}_{{\mathbf{0}}} /{\mathbf{I}} = 1 + \user2{K}_{{\user2{BSA}}} \left[ \user2{Q} \right] = 1 + \user2{kq\tau }_{0} \left[ \user2{Q} \right]$$

15 $${\mathbf{Log}}{\text{ }}\left( {{\mathbf{I}}_{{\mathbf{0}}} - {\text{ }}{\mathbf{I}}/{\mathbf{I}}} \right) = {\mathbf{logK}} + {\mathbf{n}}~{\mathbf{log}}\left[ {\mathbf{Q}} \right]$$

Herein, I_0_ and I represent the fluorescence intensities of BSA in the presence and absence of the concentration [Q] of complex respectively, while K_SV_, K_q_, and τ_0_ correspond to the quenching constant, quenching rate constant and average lifetime of tryptophan (1 × 10^− 8^ s), while K and n represent the binding constant and number of binding sites determined using the Scatchard equation.

**TiC1-TiC8** exhibited profound K_BSA_, Stern − Volmer quenching constant and K_q_, quenching rate constant values and it was found that complexes **TiC1**, **TiC3**, **TiC5** and **TiC7** appeared with higher K_BSA_ and K_q_ values, complexes **TiC1**,** TiC2** and **TiC7** displayed higher K_b_ values and **TiC2**, **TiC3** and **TiC7** reflected higher n values i.e. number of binding sites and are displayed in (Table 3).

**Table 3:** Binding factors for the BSA interaction with all the Ti(IV) complexes with BSA; ^a^K_BSA_, stern−volmer quenching constant; ^b^Kq, quenching rate constant; ^c^ Kb binding constant; ^d^n, number of binding sites.

Complex	K_BSA_^a^ (× 10^6^ M^− 1^)	K_q_^b^ (× 10^13^ M^− 1^ s^− 1^)	Kb^c^ (× 10^4^ M^− 1^)	*n* ^d^
TiC1	0.174	1.74 × 10^13^ M^− 1^ s^− 1^	0.350	1.40
TiC2	0.118	1.18 × 10^13^ M^− 1^ s^− 1^	0.155	1.46
TiC3	0.168	1.68 × 10^13^ M^− 1^ s^− 1^	0.560	1.83
TiC4	0.085	0.85 × 10^13^ M^− 1^ s^− 1^	0.898	0.96
TiC5	0.156	1.56 × 10^13^ M^− 1^ s^− 1^	0.800	1.11
TiC6	0.093	0.93 × 10^13^ M^− 1^ s^− 1^	0.721	1.08
TiC7	0.231	2.31 × 10^13^ M^− 1^ s^− 1^	0.312	1.42
TiC8	0.102	1.02 × 10^13^ M^− 1^ s^− 1^	0.430	2.37

#### BSA binding studies through synchronous fluorescence quenching

Synchronous fluorescence spectroscopy was pioneered by Lloyd and co-workers since 1970 to obtain information about the molecular environment around fluorophore molecules at low concentrations under physiological circumstances. In fact, this technique is a quick and valuable method to interpret the fluorescence quenching and possible changes in the maximum emission wavelength λ_max_^77,78^. It provides distinctive information about the tyrosine and tryptophan residues in BSA by setting Δλ to 15 nm and 60 nm, respectively. The synchronous fluorescence spectrum acquired at Δλ = 15 nm revealed the spectral characteristics of tyrosine residues, while tryptophan residues were indicated by Δλ equal to 60 nm. Tryptophan and tyrosine residues in proteins have maximum emission wavelengths influenced by the polarity of their micro surroundings; variations in these wavelengths might indicate conformational changes in the protein^79,80^.

**Fig. 7:**
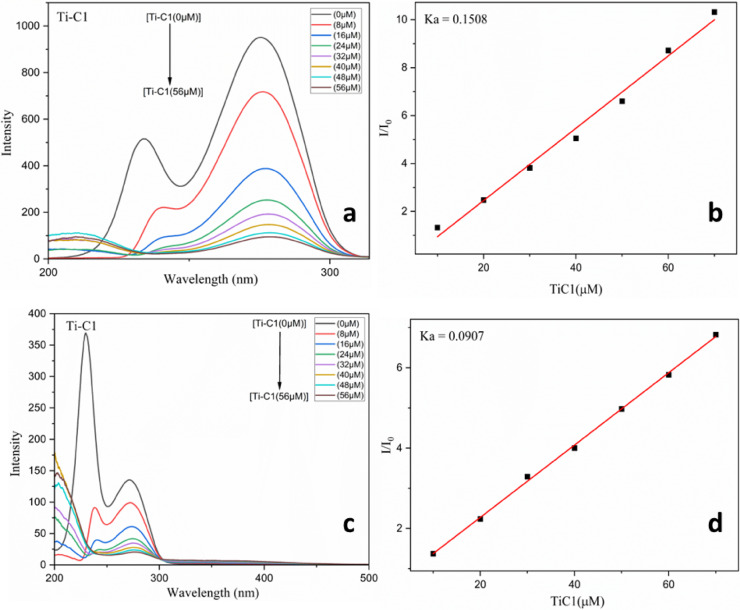
Synchronous titrations of BSA (1 × 10^− 5^ M) with **TiC1** of concentration (0–56 µM); (**a**) at Δλ = 15 nm and (**c**) at 60 nm at 298 K; (**b**) and (**d**) are the Stern-Volmer plots for **TiC1** at Δλ = 15 and 60 nm.

The information on the tyrosine and tryptophan binding microenvironments were obtained by synchronous fluorescence spectra of BSA which were obtained at different concentrations of **TiC1-TiC8**. The spectra of different chromophores could be determined by measuring the difference between the emission and excitation wavelengths (Δλ = λ_emi_ - λ_exc_) in synchronous fluorescence spectroscopy. Figure 7a, b and ESI Fig. S57 (a, c, e, g, i, k, m and o )† and Fig. S58 (a, c, e, g, i, k, m and o)† illustrate how Ti(IV) complexes affect BSA synchronous fluorescence spectroscopy with Δλ = 15 nm and Δλ = 60 nm, respectively.

Addition of the Ti(IV) complexes at λ = 15 nm gradually increased the bathochromic shift in BSA and all of the complexes realized redshift (Bathochromic shift) in the maximum emission wavelength of BSA that occurred at 275–285 nm for all the derivatives exhibiting changes in confirmation of BSA with the increase in polarity around Tyrosine residue. Figure 7a and ESI Fig. S57 (a, c, e, g, i, k, m and o)† display the binding of **TiC1-TiC8** with Tyrosine residues at λ = 15 nm.

It was observed that there was a slight drop and shift in emission magnitude corresponding to tryptophan at Δλ = 60 nm. **TiC1-TiC8** reflected slight redshift (bathochromic shift) on increasing their concentration and the emission wavelengths appeared between 270 and 280 nm as portrayed in Fig. 7c and ESI Fig. S58 (a, c, e, g, i, k, m and o)†. These observations demonstrated that the metal complexes affected the polarities and microenvironments of the tryptophan residue during the binding process. On occurring the quenching at maximum intensities, implies that the Ti(IV) complexes bind closer to the tryptophan residue where Trp in BSA experiences a redshift.

Higher quenching at Δλ = 60 nm suggests that the Ti(IV) derivatives bind to a BSA site that is closer to tryptophan residue. The results of the experiment depicted that the hydrophobicity had increased around the tryptophan and tyrosine residues, while polarity around the tryptophan and tyrosine residues has slightly decreased. Such observations illustrate how the presence of Ti(IV) derivatives alter the BSA structure. The binding constant K_a_ is provided with the help of Stern-Volmer plots using Eq. (11) and is accessible in Fig. 7b, d for **TiC1** and **TiC1-TiC8** Fig. S57 (b, d, f, h, j, l, n and p)† and Fig. S58 (b, d, f, h, j, l, n and p)†.

BSA fluorescence quenching at Δλ = 15 and 60 nm kindled the ability to bind Ti(IV) complexes with tryptophan and tyrosine residues at the same time. As previously mentioned, there was red shift in the maximum emission wavelength of tyrosine residues, but a slight discernible shift in the wavelength of tryptophan residues was also observed. This indicated that the polarity surrounding tyrosine residues was decreased and hydrophobicity had increased.

#### BSA site marker fluorescence quenching studies

BSA is a heart-shaped helical monomer collectively made up of three homologous domains named domain I, II and III. Each domain comprises of two sub-domains A and B to form a cylindrical structure. In order to identify the metal complexes competitive binding site within BSA, site marker displacement studies were carried out using fluorescence quenching studies by selecting suitable site marker probes, Warfarin (WAR) and Ibuprofen (IBU) which could bind at site I and II, respectively^81–84^. Present study has determined whether the Ti(IV) complexes bind competitively to Sudlow sites I and II - two most often discovered drug-binding sites. At 298 K, fluorescence emission measurements were taken in account to assess whether the titanium complexes would displace these probes.

**Fig. 8:**
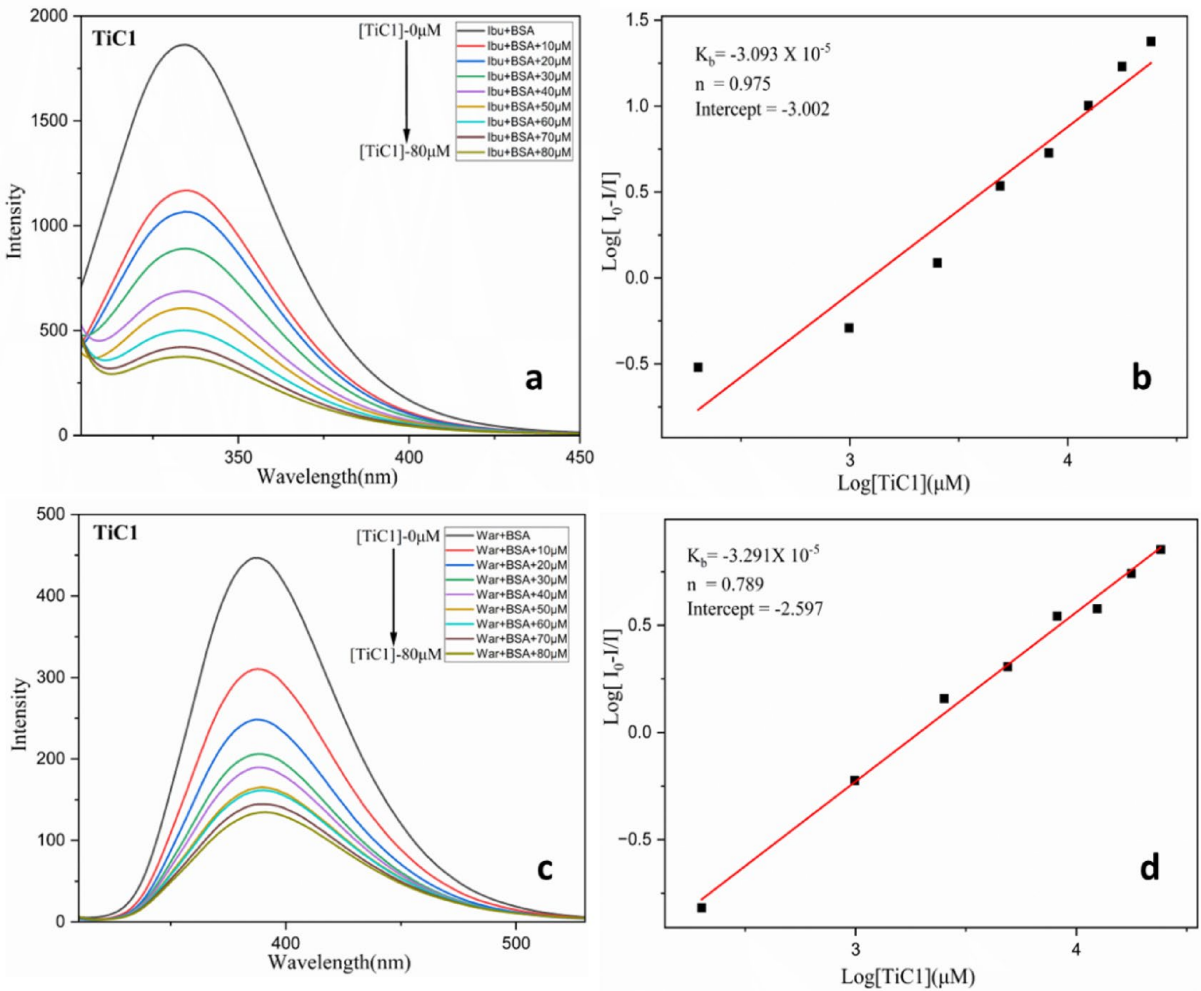
(**a**) Site marker study of BSA + Ibuprofen (1 × 10^− 5^ M) with **TiC1** of concentration (0–56 µM); (**b**) Double log plot for BSA + Ibuprofen with **TiC1**; (**c**) BSA + Warfarin ^**b**^(1 × 10^− 5^ M) with **TiC1** complex of concentration (0–56 µM); (**d**) Double log plot for BSA + Warfarin with **TiC1**.

The binding studies were carried out by titrating incremental addition of **TiC1-TiC8** to the site marker-bound BSA (1 × 10^− 5^ M). However, when connected to BSA, IBU did not exhibit this characteristic fluorescence. As a result, on addition of **TiC1-TiC8** (0–56 µM) to IBU-bound BSA, the fluorescence quenching of IBU-bound BSA (at 328 nm) was decreased gradually and a change in fluorescence intensity was observed, resulting in quenching for all complexes tending to undergo slight redshift tending to compete with IBU-bound BSA Fig. 8a and ESI Fig. S59 (a, c, e, g, i, k, m and o )†. In the probe displacement experiment with WAR, it was found that the addition of **TiC1-TiC8** (0–56 µM) caused the fluorescence intensity of WAR to quench; it occurred at greater wavelength than the maximal emission wavelength of WAR unbound BSA. This is because when WAR interacted with BSA, the hydrophobic drug displayed characteristic fluorescence at around (380 nm) for all the complexes. The competitive interaction of Ti(IV) complexes with WAR was confirmed by dimming of fluorescence intensity with redshift as a function of **TiC1-TiC8** concentration (Fig. 8c and ESI Fig. S60 (a, c, e, g, i, k, m and o)†).

**Table 4:** The comparison of binding constants of the **Ti(IV)** complexes with BSA before and after the addition of site marker probes(IBU and WAR) at 298 K; K_b_^a^binding constant.

Complexes	BSA K_b_^a^ (× 10^4^ M^− 1^)	BSA + Ibuprofen K_b_^a^ (× 10^4^ M^− 1^)	BSA + Warfarin K_b_^a^ (× 10^4^ M^− 1^)
TiC1	0.350	0.309	0.329
TiC2	0.155	0.348	0.367
TiC3	0.560	0.375	0.384
TiC4	0.898	0.334	0.482
TiC5	0.800	0.363	0.471
TiC6	0.721	0.336	0.460
TiC7	0.312	0.283	0.401
TiC8	0.430	0.144	0.412

Equation (13) was used to investigate the binding constant in the presence of site markers. The fluorescence intensity of BSA with site marker (warfarin or ibuprofen) added is similar to that of BSA without site marker, making it easier to compare how warfarin or ibuprofen affect the Ti(IV) complexes ability to bind with BSA. Double log plots determined the binding constant K_b_ (Fig. 8b, d, ESI Fig. S59 (b, d, f, h, j, l, n and p) † and Fig.S60 (b, d, f, h, j, l, n and p )†). K_b_ values were observed to be similar as of complexes containing free BSA, indicating a competition between Ti(IV) complexes, IBU and WAR for Sudlow’s sites I and II. As seen in Table 4, this led to a lower binding constant. However, the Ti(IV) complexes had substantially displaced IBU and WAR during their interaction with BSA.

### Molecular docking with BSA and DNA

Employing molecular docking experiments, binding mechanisms, properties and affinities of Ti(IV) complexes with DNA and BSA were investigated. Lamarckian genetic algorithm (LGA) was applied in all docking studies using Autodock vina 4.2 molecular docking software and Discovery Studio was taken in account to visualize the docked structures^85–87^.

**Fig. 9:**
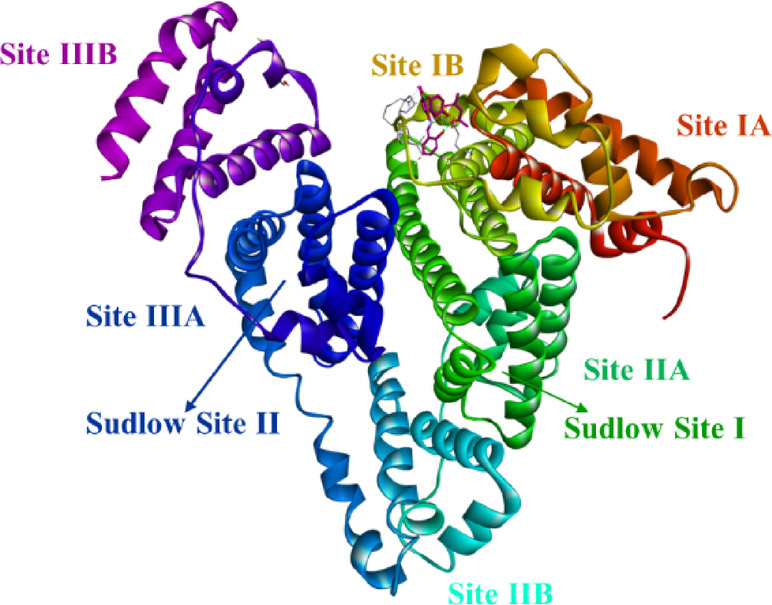
Binding sites of molecules in BSA protein.Binding sites of molecules in BSA protein.

The crystal structures of DNA (PDB ID: 1BNA) and BSA (PDB ID: 4F5S) were obtained from the protein data bank and required modifications were made using the Swiss model online tool. BSA consists of three primary domains, further each domain is classified into 2 sub-domains namely IA, IB, IIA, IIB, IIIA and IIIB as displayed in (Fig. 9). The most electrostatic surfaces are found between domain II residues. Titanium derivatives were best fitted in between domains IB and IIA. BSA comprises of unique polypeptide chain of 583 amino acid units and a molecular mass of 66,200 Da, it is said to be abundant in albumin in plasma while assisting in carrying out and delivering the drug to the targeted spots as it is.

**Fig. 10:**
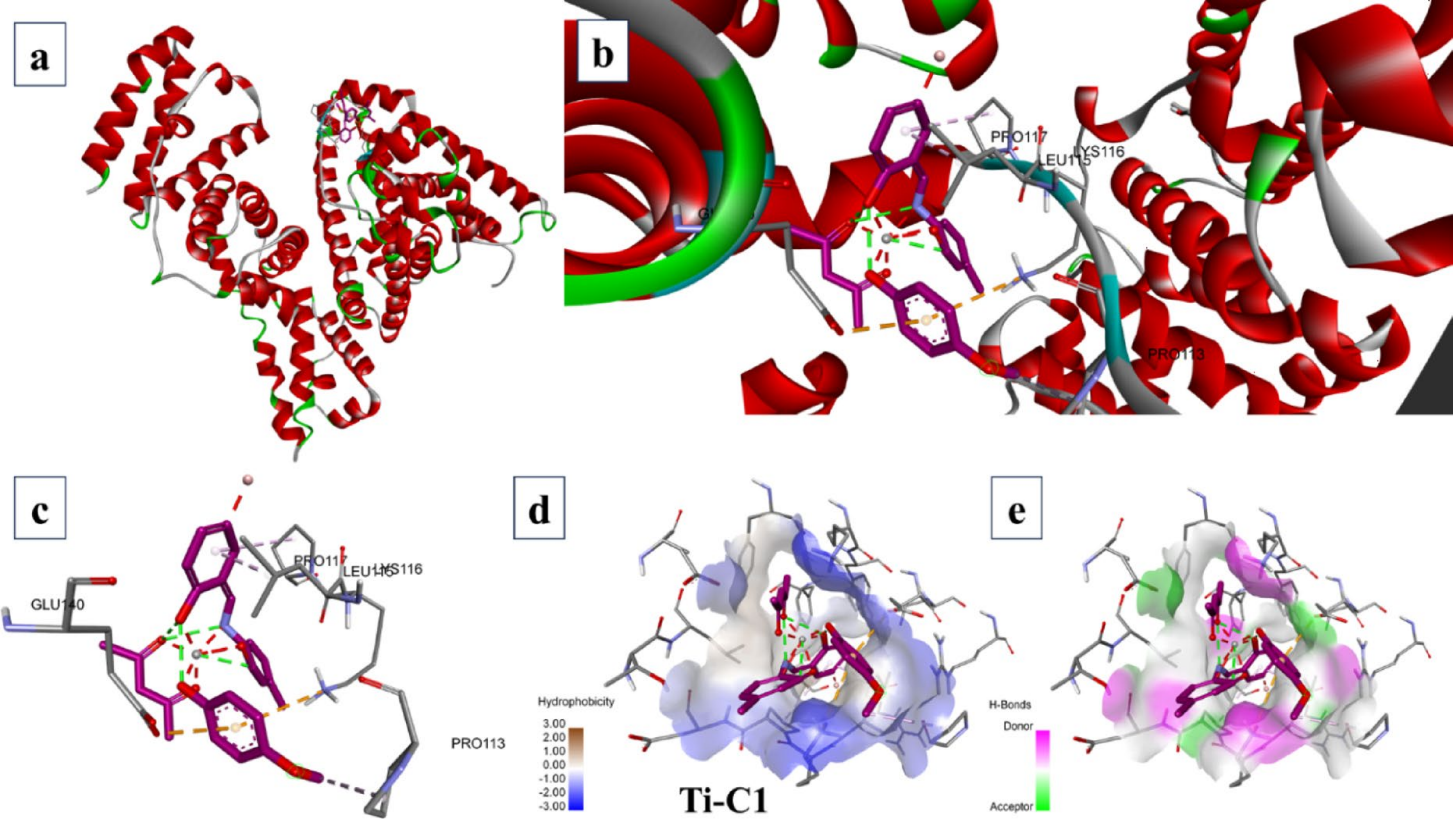
Binding sites of **TiC1** with BSA; (**a**) Binding site on BSA; (**b**) Elaborated binding site; (**c**) Expanded view of interactions; (**d**) Hydrogen bonding around the complex and; (**e**) Hydrophobicity around the **TiC1**.

According to the molecular docking data, by varying the grid dimensions of x, y and z to values of 34.885, 23.976 and 98.792, respectively with spacing 0.375, it was possible to dock **TiC1-TiC8** with BSA. The docking results were congregated according to the binding energies. Finally, the highest cluster members and structures with the lowest binding energy were recorded. The Ti(IV) complexes were binding between Site IB and Site IIA as displayed in Fig. 10a and ESI Fig. S61(a) − 68(a)†,

**TiC1** was able to bind with distinct amino acids of bovine serum albumin like PRO117, LYS115, PRO113, GLU140 and LYS116 Fig. 10b, c and ESI Fig. S61 (b and c)†, **TiC2** binds to LYS116, LYS114, PRO516, ASP517 and THR518 ESI Fig. S62 (b and c)†, **TiC3** binds to LEU115 and LYS136 ESI Fig. S63 (b and c)†, **TiC4** binds to LYS136, ILE141, GLU140, LEU115 and TYR137 ESI Fig. S64 (b and c)†, **TiC5** binds to LYS136, PHE133, TYR160 and TYR160 ESI Fig. S65 (b and c)†, **TiC6** binds to ARG144, SER109, PRO110 and LYS114 ESI Fig. S66 (b and c)†, **TiC7** binds to SER109, PRO110, ARG144 and LYS114 ESI Fig. S67 (b and c)† and **TiC8** binds to HIS145, ILE522 and VAL423 ESI Fig. S68 (b and c)†. The docking scores of the complexes are found to be -9.0 kcal mol^− 1^ for **TiC1**, -9.7 kcal mol^− 1^ for **TiC2**, -9.2 kcal mol^− 1^ for **TiC3**, -9.3 kcal mol^− 1^ for **TiC4**, -11.2 kcal mol^− 1^ for **TiC5**, -8.7 kcal mol^− 1^ for **TiC6**, -8.6 kcal mol^− 1^ for **TiC7** and − 10.2 kcal mol^− 1^ for **TiC8.** The hydrophobic property exhibited by the complexes was also found higher at the phenyl rings of the complexes supported by the ligand **(L1)** sub-ligands (phenol derivatives) Fig. 10d and ESI Fig. S61d – 68(d)†. All Ti(IV) derivatives exhibited hydrogen bonding near the ligands and sub-ligands region Fig. 10e and ESI Fig. S61(e)–68(e)†. Similarly, the charge of the complexes is said to have higher values as it was supported by high *p*-electron density supported by the substitution moieties supporting phenyl rings of sub-ligand (phenol derivatives) of the complex and is said to be basic at the acetylacetone end and neutral towards the entire ligand distribution site.

**Fig. 11:**
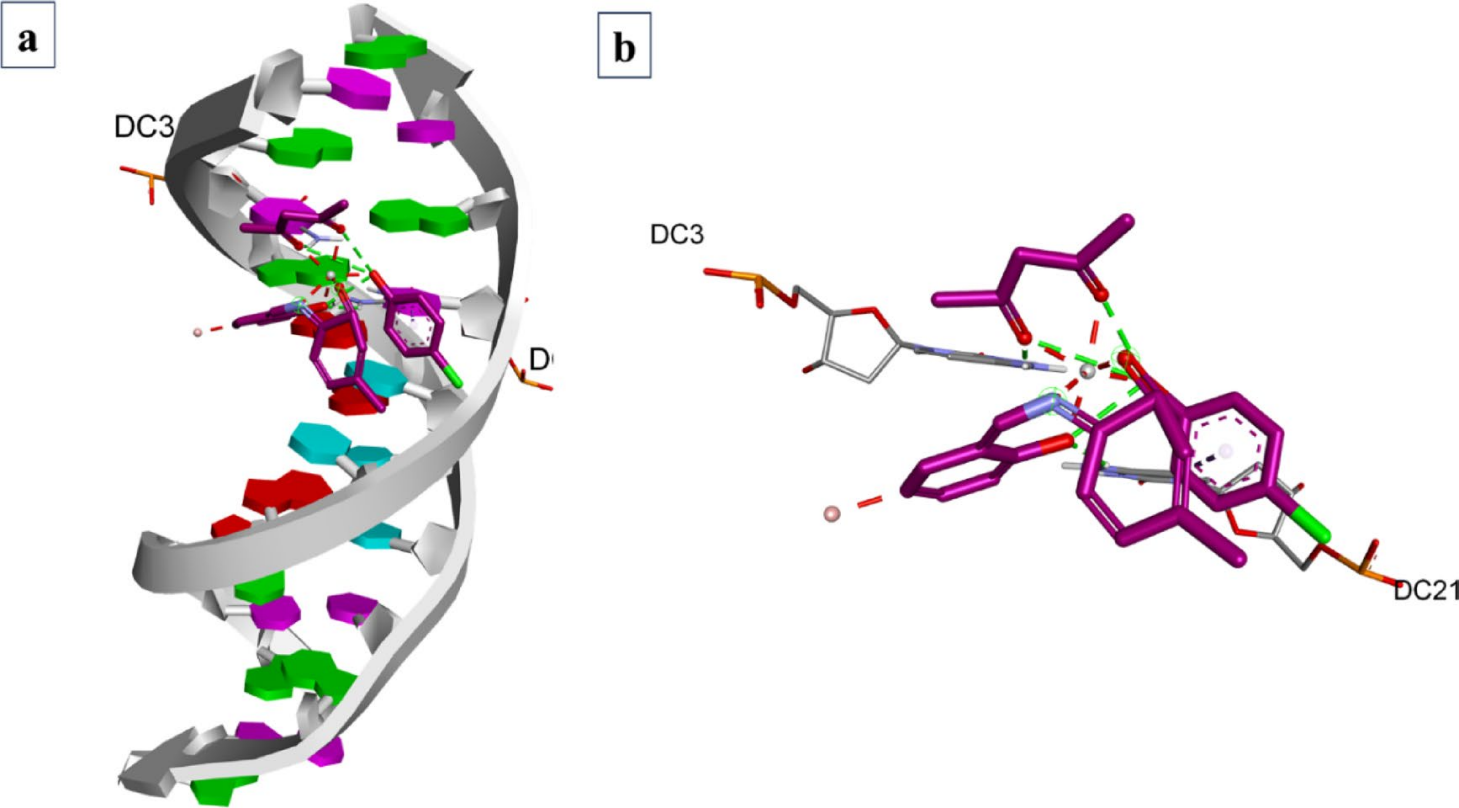
Binding mode of **TiC1** with DNA.

**TiC1-TiC8** were subjected to molecular docking investigations in the presence of DNA (PDB ID: 1BNA), with the central grid points of the maps being remapped to x = 14.780, y = 20.976, and z = 8.807 with spacing 0.375. It was observed that there was strong groove binding between the complexes and the DNA dodecamer as displayed in Fig. 11a, b and ESI Fig. S69(a, b)†. As a result, when attached to DNA, these complexes had significant binding energies ranging from − 8.3 to -10.7 kcal mol^− 1^. Among all the titanium derivatives, **TiC8** exhibited the best binding affinity (free energy of binding = 10.3 kcal mol^− 1^) ESI Fig. S69(o and p)†.

Similarly, the outcomes of molecular docking of the complexes with DNA also established favorable binding energies − 8.3 kcal mol^− 1^ for **TiC1**, ESI Fig. S69(a and b)†, -8.6 kcal mol^− 1^ for **TiC2**, ESI Fig. S69(c and d)†, -8.7 kcal mol^− 1^ for **TiC3**, ESI Fig. S69(e and f)†, -9.5 kcal mol^− 1^ for **TiC4**, ESI Fig. S69(g and h)†, -8.9 kcal mol^− 1^ for **TiC5**, ESI Fig. S69(i and j)†, -8.3 kcal mol^− 1^ for **TiC6**, ESI Fig. S69(k and l)†, -8.4 kcal mol^− 1^ for **TiC7** ESI Fig. S69(m and n)† and − 10.3 kcal mol^− 1^ for **TiC8**, ESI Fig. S69(o and p)†. It is visible that the Ti(IV) complexes bind *via* groove binding mode with slight intercalation to DNA helix possibly allowing the complexes to bind nucleotides depending on their substitution as displayed in ESI Fig. S69(a-p)†. Molecular docking results exhibit the highest negative values of energy while implying a powerful interaction between DNA and above said titanium complexes.

### DFT studies

All computations were performed using the Gaussian 09 (G 09) computational codes by applying Becke’s three-parameter hybrid exchange and the Lee-Yang-Parr non-local correlation function (B3LYP) using the density functional theory (DFT)^88^. For all the calculations, standard basis set 6-311G (d, p) for lighter elements (C, H, N O, F, Cl and Br) and LanL2DZ effective core potential for Ti metal was used. Geometry of Ti(IV) complexes was optimized to zero negative vibration frequency to represent the local minima associated with positive eigenvalues. Vertical electronic excitations based on B3LYP were obtained with time-dependent density functional theory (TD-DFT) using the ground state optimized geometry. In order to account for the solvent effect, both the DFT and TD-DFT studies were associated with the conductor-like polarizable continuum model (CPCM) in a dimethyl sulfoxide (DMSO) medium.

**Fig. 12:**
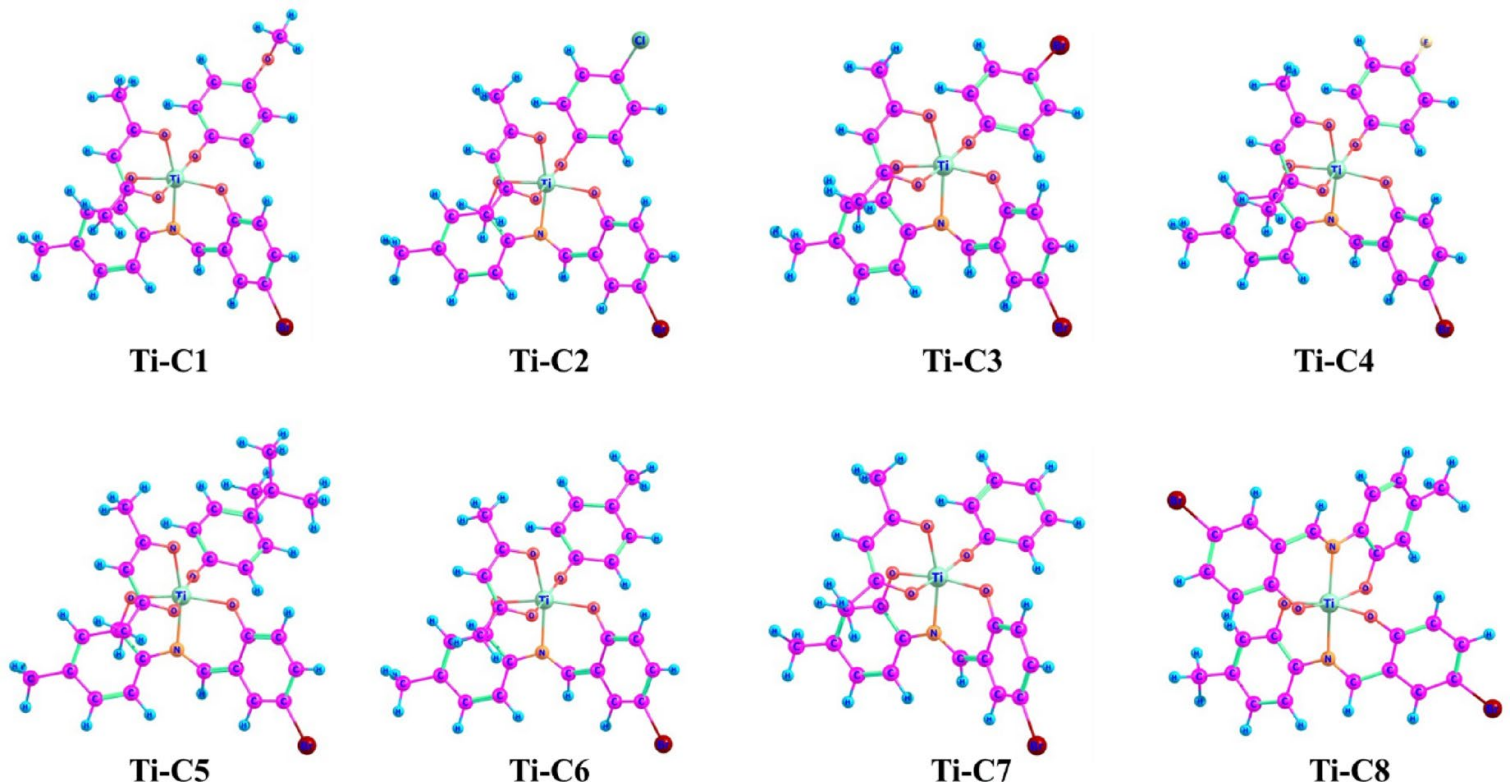
Optimized molecular geometry of Ti(IV) complexes.

Computational studies of the synthesized Schiff base 4-bromo-2-(((2-hydroxy-4-methylphenyl)imino)methyl)phenol with pentane-2,4-dione and substituted phenol derivatives in complex with Ti metal ions for **TiC1-TiC8** were carried out using combined DFT-B3LYP method by means of Gaussian 09 computational tool. Different quantum chemical parameters were calculated by applying B3LYP/6-31G**/LanL2DZ ECP methods such as geometry optimization, molecular energy, ESP charges, energy of frontier molecular orbitals and bandgap. All Ti(IV) complexes exhibited distorted octahedral geometry and the corresponding key bond lengths are revealed in Fig. 12 and ESI Table S2†.

**Fig. 13:**
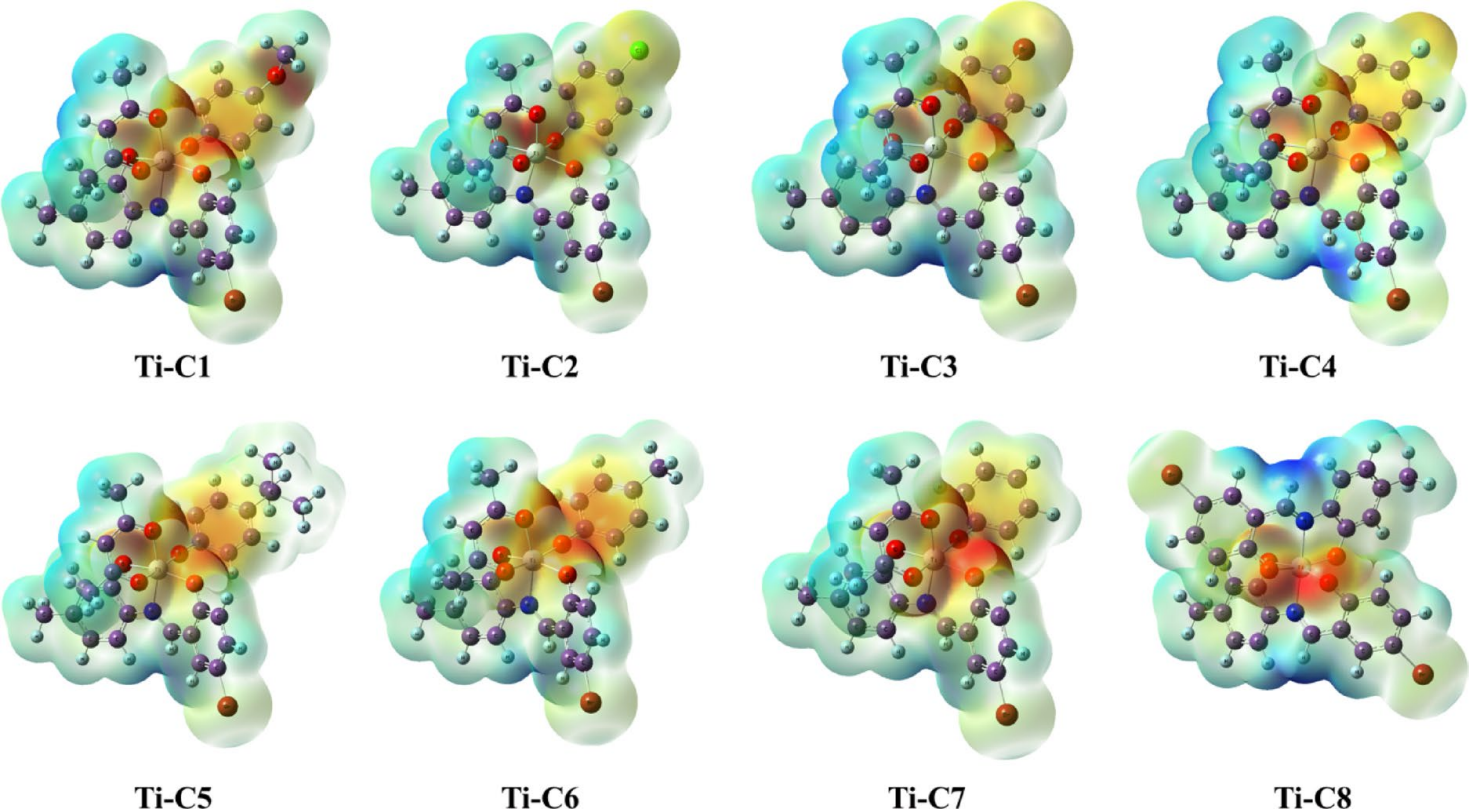
Electrostatic potential mapped on the surface of the optimized molecular geometry of Ti(IV) complexes using DFT/B3LYP-LanL2DZ method.

Electrostatic potential mapping (ESP) was carried out to determine the optimal geometry on the Van der Walls surface. Electrostatic potential mapped onto the constant electron density surface was extremely supportive in studying the relationship between photophysical properties and molecular structure as well as hydrogen bonding interactions in the complexes. Maximum negative region as the preferred site for an electrophilic attack, is indicated as red and the maximum positive region as the preferred site for the nucleophilic attack, appears in blue colour (Fig. 13).

**Fig. 14:**
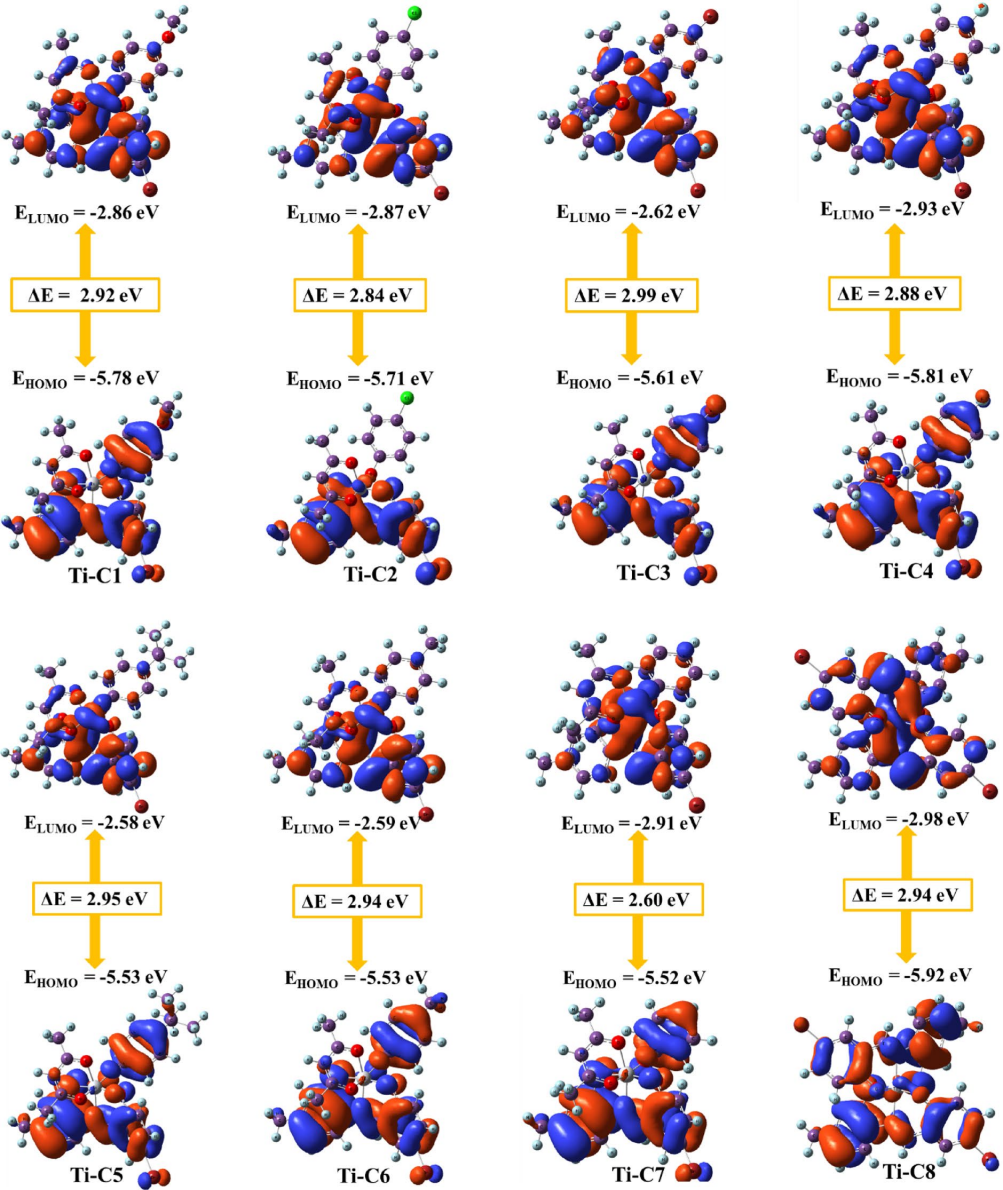
FMOs of Ti(IV) complexes using the DFT/B3LYP method.

Frontier molecular orbitals (FMO) comprising the highest occupied molecular orbital (HOMO) and the lowest unoccupied molecular orbital (LUMO) with the energy gap between HOMO and LUMO of all Ti(IV) complexes were calculated and displayed in (Fig. 14; Table 5). **TiC1-TiC8** exhibits the band gap energy range from 2.60 to 2.99 eV. Furthermore, the energy gap (ΔE) is an important parameter to characterize the chemical reactivity and kinetic stability of the molecules^89,90^. The energies of frontier molecular orbitals (E_HOMO_ and E_LUMO_), energy band gap (ΔE) which explains the eventual charge transfer interaction within the molecule, electronegativity (χ), chemical potential (µ), global hardness (ɳ), global softness (S) and global electrophilicity index (ω) are enlisted in (Table 5). The importance of these parameters is to measure the molecular stability and reactivity. The electrophilicity index is one of the most important quantum chemical parameters in describing the toxicity of various compounds in terms of their reactivity and site selectivity. Also, the electrophilicity property quantifies the biological activity of drug-receptor interaction.

**Table 5:** Calculated molecular electronic parameters of Ti(IV) complexes.

S. no	Code	Energy (Kcal/mol)	DM	HOMO (eV)	LUMO (eV)	Gap (eV)	Χ (eV)	Μ (eV)	ɳ (eV)	S (eV)	ω (eV)
1	TiC1	-2610725.93	7.09	-5.78	-2.86	2.92	4.32	-4.32	-1.46	-0.73	-13.59
2	TiC2	-2826128.51	9.74	-5.71	-2.87	2.84	4.29	-4.29	-1.42	-0.71	-13.04
3	TiC3	-4156258.55	9.07	-5.61	-2.62	2.99	4.11	-4.11	-1.50	-0.75	-12.66
4	TiC4	-2601096.44	8.44	-5.81	-2.93	2.88	4.37	-4.37	-1.44	-0.72	-13.74
5	TiC5	-2635716.93	5.76	-5.53	-2.58	2.95	4.05	-4.05	-1.48	-0.74	-12.12
6	TiC6	-2561412.08	5.91	-5.53	-2.59	2.94	4.06	-4.06	-1.47	-0.73	-12.12
7	TiC7	-3035016.14	9.14	-5.52	-2.91	2.60	4.21	-4.21	-1.30	-0.65	-11.56
8	TiC8	-4218631.57	1.68	-5.92	-2.98	2.94	4.45	-4.45	-1.47	-0.73	-14.53

**Fig. 15:**
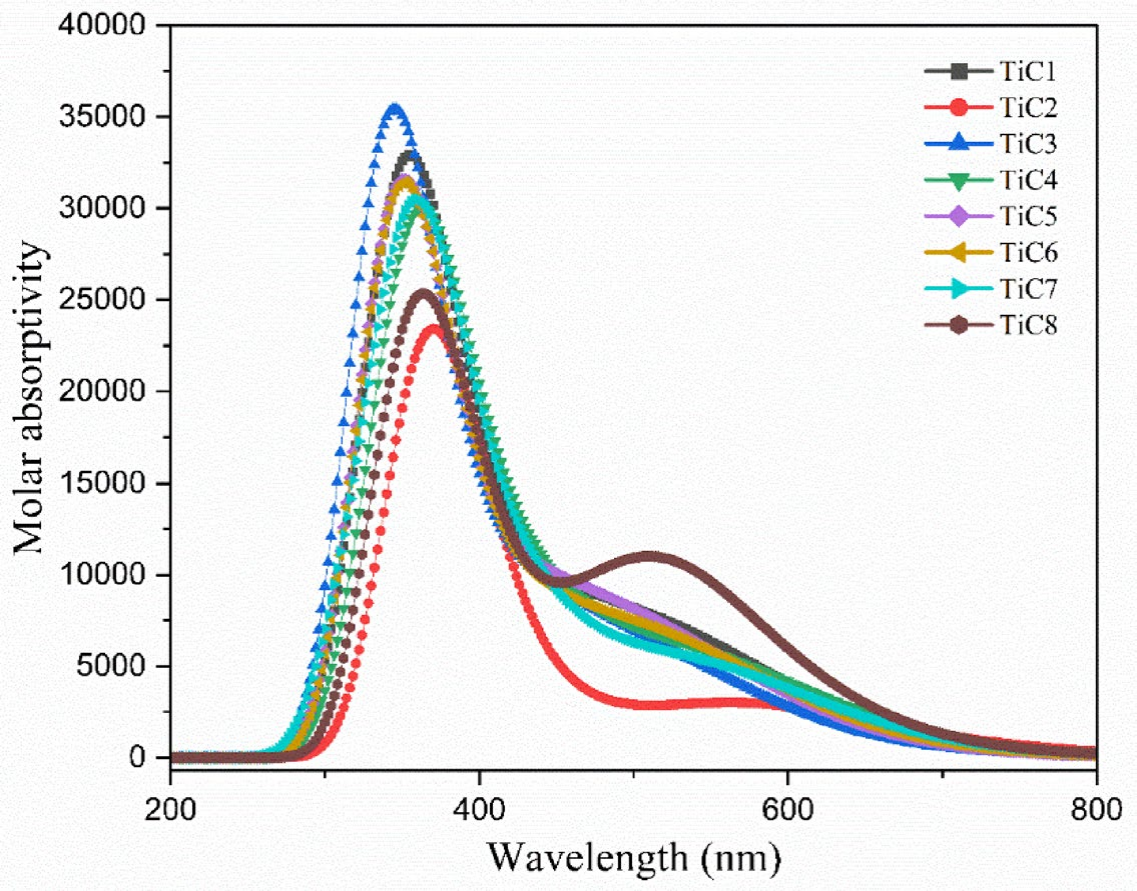
TD-DFT UV-Vis spectra for Ti(IV) complexes in the DMSO medium.

The ground state optimized geometries were performed for TD-DFT^91^ calculations and the corresponding electronic transitions with their associated orbital contributions were carried out. Ti(IV) complexes displayed strong electronic bands, appearing in the range of 350 to 370 nm for the π→π* transition and the small charge transfer band appeared at around 500 nm. Comparatively, both the experimental and predicted absorbance bands were in coordination with their characteristic electronic transition as provided in ESI Table S3†. Also, the obtained UV-Vis spectra from TD-DFT calculations for **TiC1-TiC8** are made available in Fig. 15, which are in accordance with the experimental absorbance bands.

### DPPH assay

Free radical scavenging activity of titanium derivatives at 517 nm was performed using the 2,2-diphenyl-1-picrylhydrazyl (DPPH) technique^92^. It examined the interaction of DPPH with **TiC1-TiC8** individually at different concentrations (9.4, 18.8, 37.5, 75, and 150µM/uL) as displayed in ESI Fig. S70†. Using ascorbic acid as a standard, the findings of antioxidant activity of these titanium(IV) complexes are displayed in (Table 6). These results suggest that the complexes could be ranked according to their IC_50_ values, where **TiC2** exhibited the lowest IC_**50**_ with the highest radical scavenging activity among all the titanium derivatives.

**Table 6:** IC_50_ values of DPPH assay for synthesized Ti(IV) complexes.

S.no	Code	IC_50_ values µM/uL
1	TiC1	93.71 ± 0.1
2	TiC2	42.88 ± 0.3
3	TiC3	56.86 ± 1.2
4	TiC4	76.99 ± 0.1
5	TiC5	63.64 ± 1.0
6	TiC6	73.93 ± 0.1
7	TiC7	85.28 ± 0.7
8	TiC8	67.08 ± 0.2

### MTT assay

**Fig. 16:**
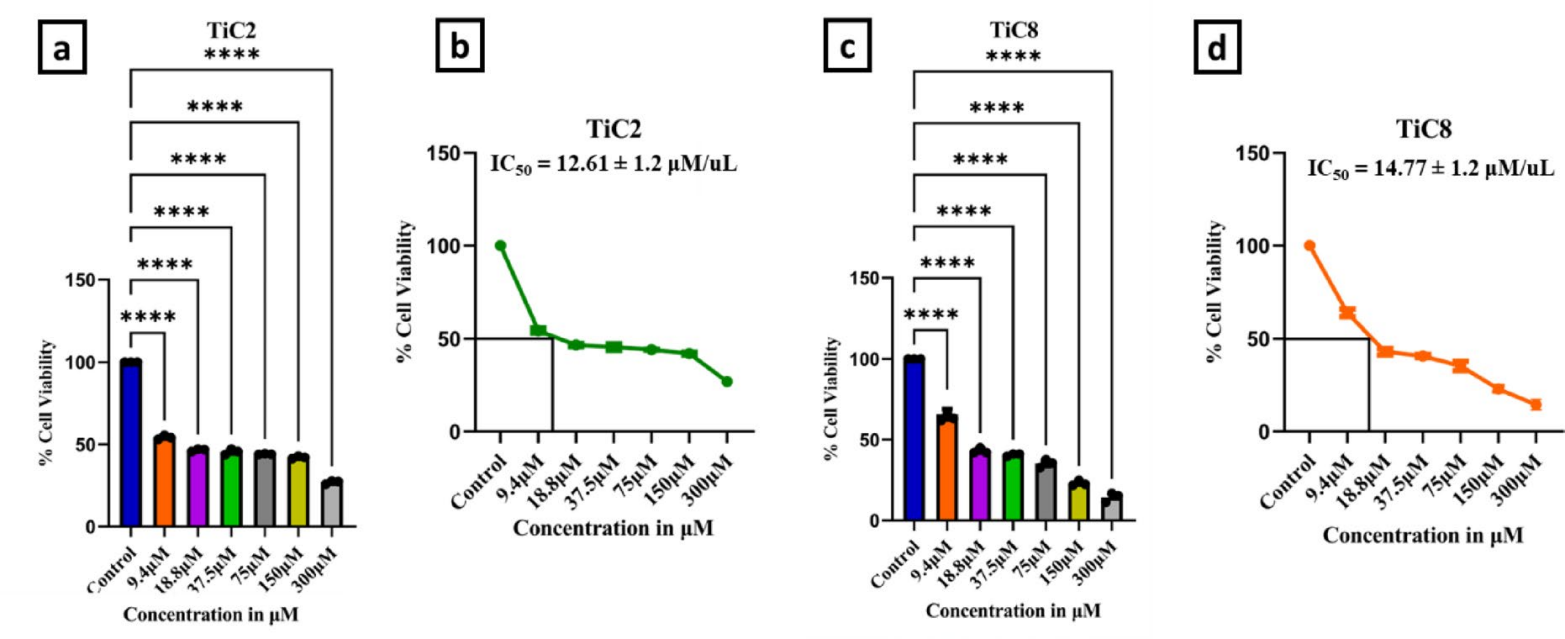
MTT assay and IC_50_ value determination: cell inhibition (%) of HeLa cell line of **TiC2** (**a**,**b**) and **TiC8** (**c**,**d**). All values are expressed as mean ± SEM. ****p < 0.0001 denotes statistical significance as determined by MTT Assay, Error bars indicate the standard deviation from three independent experiments.

Using standard 3-(4,5-dimethylthiazol-2-yl)-2,5-diphenyltetrazolium bromide (MTT) assay protocol, the in vitro cytotoxicity of all newly developed titanium(IV) complexes, Ligand[L1] and cisplatin as control were investigated in triplicates alongside a panel of cancer cell lines, namely human epitheloid cervix carcinoma (HeLa), breast cancer (MCF7) and normal human embryonic kidney cells (HEK-293) cell lines^93^. Cisplatin was the typical positive control drug. On treatment with (9.4, 18.8, 37.5, 75, 150 and 300µM) of Ti(IV) complexes, morphological changes were detected in the HeLa cell line, all the complexes displayed notable cytotoxicity (12.61 − 62.80 µM/uL); however, **TiC2** and **TiC8** outperformed compared to the other Ti(IV) complexes of the series in terms of potency and selectivity in HeLa cell lines, IC_50_ = 12.61 ± 1.2 µM/uL, selectivity > 14 for **TiC2**, IC_50_ = 14.77 ± 1.3 µM/uL, selectivity > 16 for **TiC8**. Figure 16, ESI Fig. S71, S72 and S73† and Table 7 reflects the notable potency of titanium derivatives against HeLa cells with their IC_50_ values concerned. Cytotoxicity of these Ti(IV) complexes appeared meagre in MCF7 cell line, while the cytotoxicity of potent **TiC2** and **TiC8** was completely outperformed by non-cancerous HEK-293 cell lines. In the IC_50_ merit of **TiC2** and **TiC8**, these complexes were subjected for further cytotoxic investigation employing additional tests.

**Table 7:** Preliminary MTT assay of synthesized Ti(IV) complexes at 48 h of drug exposure and calculated IC_50_ values.

S. no	Code	IC_50_ values in µM/uL		
		HeLa	MCF7	HEK-293
1	TiC1	55.66 ± 0.3	100.25 ± 1.6	-
2	TiC2	12.61 ± 1.2	69.63 ± 0.3	> 250
3	TiC3	39.03 ± 0.9	102.40 ± 1.6	-
4	TiC4	62.80 ± 1.1	66.74 ± 1.3	-
5	TiC5	49.73 ± 0.2	77.10 ± 0.9	-
6	TiC6	52.09 ± 2.0	90.45 ± 1.5	-
7	TiC7	57.12 ± 1.8	101.37 ± 0.6	-
8	TiC8	14.77 ± 1.3	94.21 ± 0.8	> 250
9	Cisplatin	10.68 ± 1.3	11.96 ± 1.1	> 250
10	L1	23.56 ± 0.4	35.65 ± 1.4	-

### AO/EB STAINING

AO-EB dual staining technique is a qualitative technique used to determine and understand apoptosis and helps in classifying living, early apoptotic, late apoptotic and necrotic cells using fluorescent pictures to record changes in the nuclear morphology of the cells. The underlying mechanism of titanium complexes’ high cytotoxicity and antiproliferative activities is based on fluorescence emission and cell morphological characteristics and could be validated by the dual AO-EB fluorescent staining method^94,95^. Physical characteristics of apoptotic cells include DNA fragmentation, chromatin condensation, phosphatidylserine (PS) translocation to the extracellular side, cytoplasmic cell shrinkage and plasma membrane blebbing. Nucleic acid binding dyes are AO and EB, the nuclei of both living and dead cells could be stained with AO. On the other hand, only the cells with damaged membranes are stained by EB. Living cells fluorescence is uniformly green, while necrotic cells appear to be uniformly orange to red under the confocal microscope. The green glowing patches that result from chromatin condensation and DNA fragmentation are apoptotic cells. Because of the highly ordered cell structure of living cells, the cells in the control group are dense and produce a consistent green light. The potent **TiC2** (12.61 ± 1.2 µM/uL) and **TiC8** (14.77 ± 1.2 µM/uL) complexes were cultivated in HeLa cells and underwent AO-EB staining using the standard protocol.

**Fig. 17:**
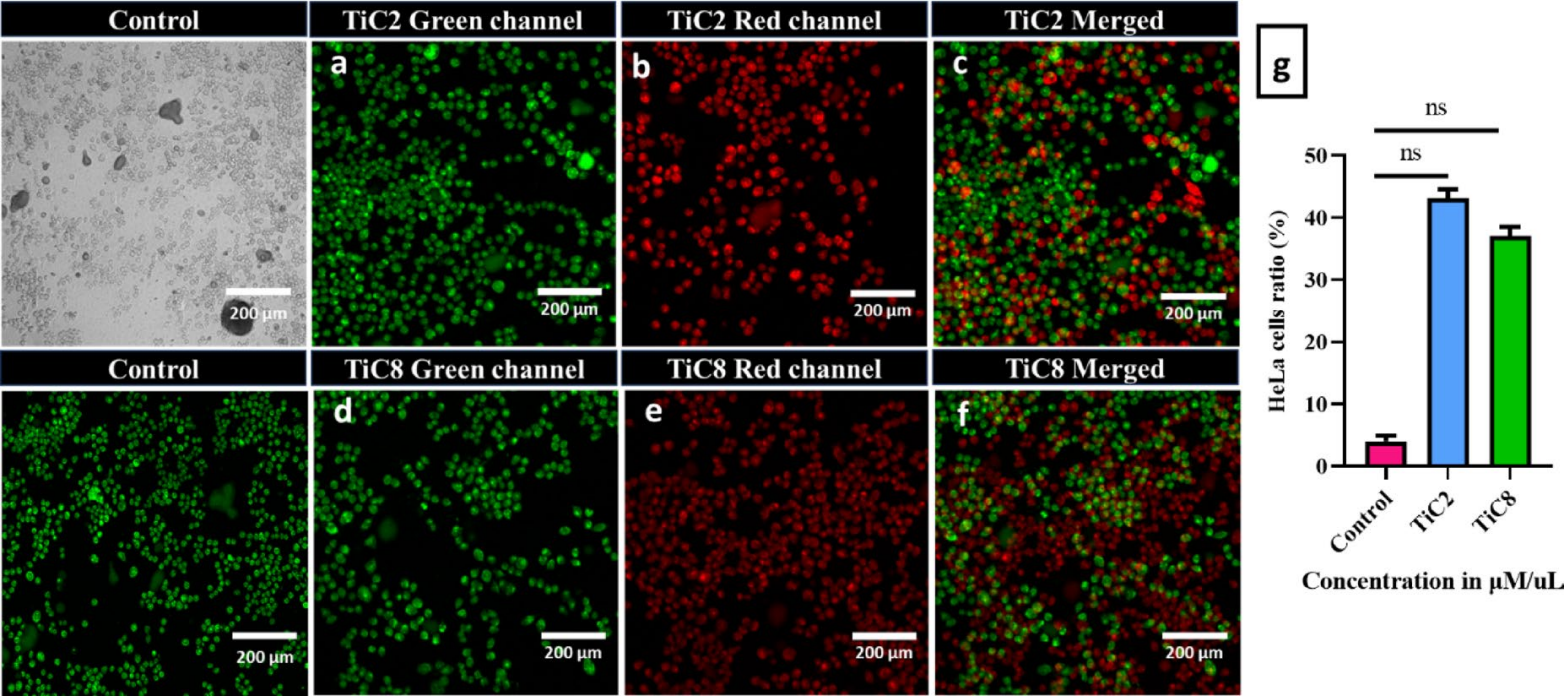
**i.** (**a**–**c**) AO-EB staining of HeLa cells with **TiC2** (12.61μM/uL, equivalent concentrations) for 48 h; **ii**. (**d**–**f**) AO-EB staining of HeLa cells **TiC8** (14.77μM/uL, equivalent concentrations) for 48 h; **iii.** (**g**) % Cell population in the microscopic field in AO-EB staining graph represented as the mean ± s.d; ns *p* < 0.033.

Due to the Ti(IV) complexes’ induction of apoptotic cell death through the breakdown of the cancer cell membrane, EB entered the cell and overpowered the fluorescence of AO to generate a greenish-orange stain. Consequently, both the complexes caused apoptosis of approximately 45% in the case of **TiC2** and 37% (Fig. 17a–c) in the case of **TiC8** as depicted in (Fig. 17d–f). In particular, the potency of **TiC2** was higher than that of **TiC8**, as it revealed a drop in the number of living cells and an increase in apoptotic bodies and displayed in (Fig. 17g).

### Flow cytometry for the detection of cell cycle arrest

**Fig. 18:**
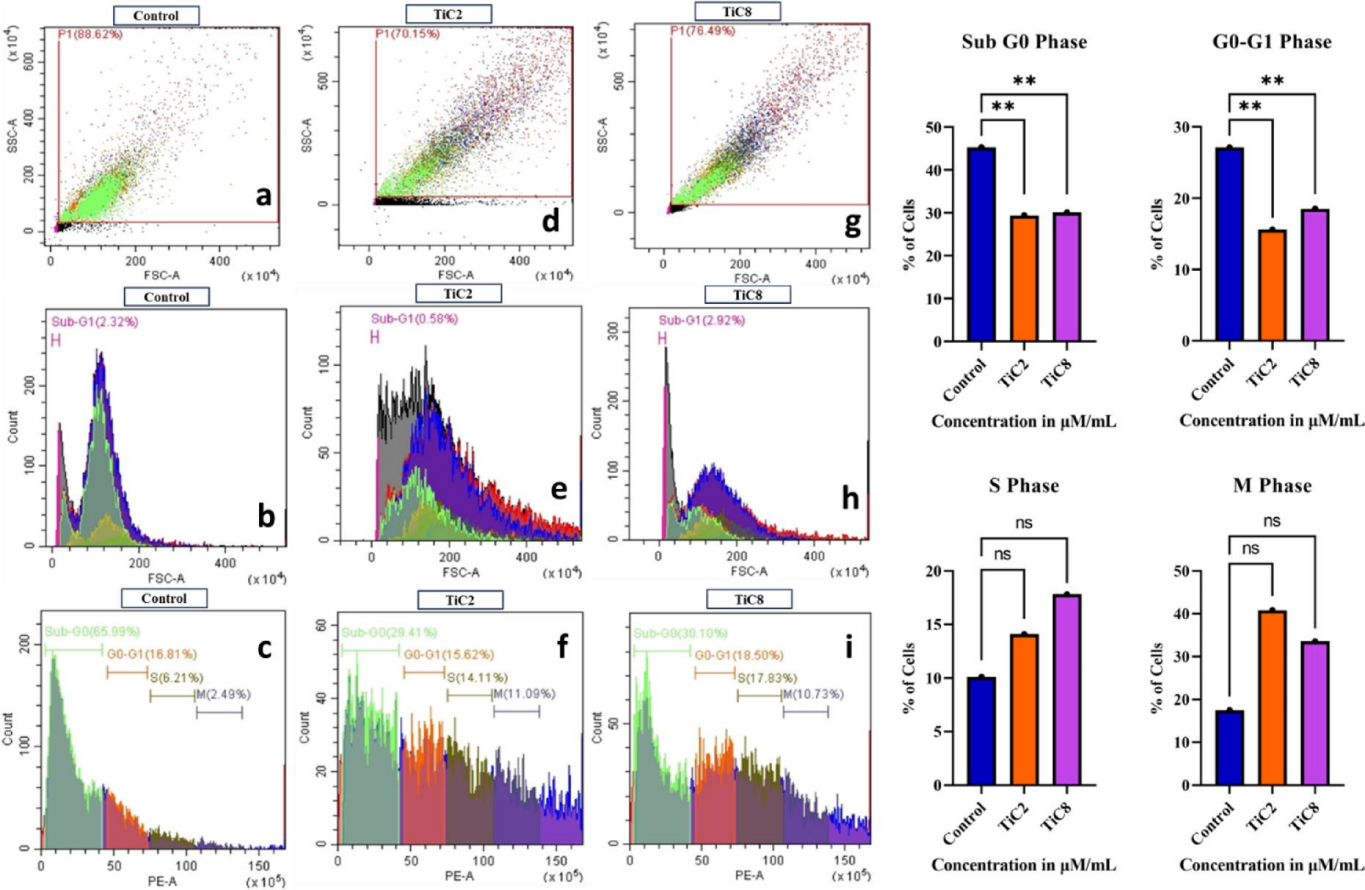
Cell cycle analysis of HeLa cells by PI staining for 48 h; **(i)** Control (**a**–**c**); **(ii) TiC2** (12.61µM/uL, equivalent concentrations (**d**–**f**); **ii. TiC8** (14.77µM/uL, equivalent concentrations (g, h and i); **(iii)** Graphical representation of the different phases of the cell cycle on the treatment of **TiC2** and **TiC8**; The P values of > 0.12 (ns), 0.033 (*), 0.002 (**), were considered as significant.

Propidium iodide cell labelling has been used with fluorescence-activated cell sorting (FACS) in the measurement of cellular DNA to examine cell cycle progression and assess the potency of the complexes^96,97^. Here, a distribution of cells in various phases is revealed by the unambiguous univariate analysis of DNA content. After 48 h exposure of HeLa cell lines with IC_50_ concentration of **TiC2** (12.61 ± 1.2 µM/uL) and **TiC8** (14.77 ± 1.3 µM/uL), it was noted that **TiC2** and **TiC8** impacted on a sequence of processes related to cellular division in HeLa cells. According to FACS data, it was observed that in the control condition, HeLa cell lines exhibited the Sub-G0 phase accounting for around 45.25% ofthe cells in the control well, followed by the G0-G1 phase (27.12%), S phase (10.11%) and M phase (17.52%) as displayed in (Fig. 18a–c; Table 8). HeLa cell lines Sub-G0 phase dropped to 29.41% after treatment with **TiC2** (12.61 µM/uL) and in the G0-G1 phase (15.62%), whereas, there was increase in both S phase (14.11%) and M phase (40.86%) cell cycle by activity of **TiC2** as presented in (Fig. 18d–f; Table 8). Similarly, on treatment of **TiC8** (14.77µM/uL) to the HeLa cell line, there was a slight decrease in the G0-G1 phase (30.10%) and G0-G1 phase (18.50%) while the upsurge was observed in the S phase (17.83%) and M phase (33.57%) of the cell cycle as displayed in presented in (Fig. 18g–i) and Table 8). **TiC2** and **TiC8** were effective against HeLa cancer cells and could induce cell cycle arrest in the G0-G1, S and M phases of the cell cycle, all the findings of the cell cycle analysis are displayed in (Fig. 18; Table 8).

**Table 8:** Tabular representation of phases of the cell cycle analysis on different treatments of **TiC2** and **TiC4**.

Sl.no	Condition	Sub G0 phase (%)	G0-G1 phase (%)	S phase (%)	M phase (%)
1	Control	45.25	27.12	10.11	17.52
2	**TiC2** (12.61µM/uL)	29.41	15.62	14.11	40.86
3	**TiC8** (14.77µM/uL)	30.10	18.50	17.83	33.57

### Generation of reactive oxygen species (ROS)

The amount of ROS (reactive oxygen species) is essential for maintaining normal cell function. It opens the door for human disorders on its interference. Herein, ROS levels in HeLa cells were examined by treating with the IC_50_ concentration of **TiC2** (12.61 ± 1.2µM/uL) and **TiC8** (14.77 ± 1.3µM/uL). ROS assay is frequently used to analyze oxidative stress^98^. Wherein, cellular esterase effectively deacetylates the nonpolar dye DCFH-DA (2′,7′Dichloro dihydro-fluorescein diacetate) to DCFH, which is undetectable under a microscope. Later, it is oxidized to fluorescent DCF (2′,7′Dichloro-dihydro-fluorescein) with the help of cytoplasmic ROS. The amount of ROS in cells is closely associated with the fluorescence intensity.

**Fig. 19:**
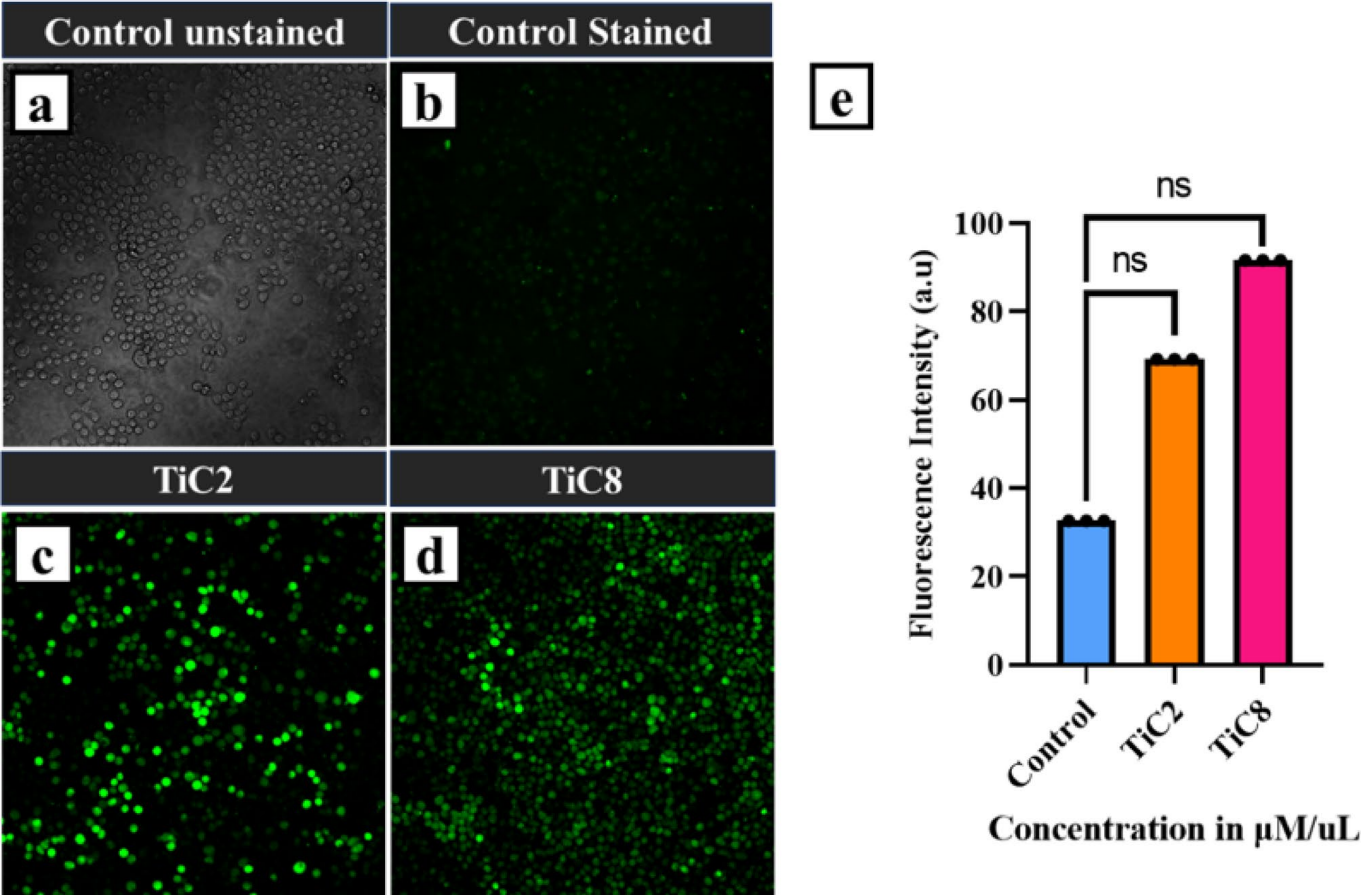
Fluorescence images of ROS levels in HeLa cells treated (**a**) Control unstained, (**b**) Control stained and treated IC_**50**_ equivalent concentrations of (**c**) **TiC2** and (**d**) **TiC8** for 48 h. (**e**) Graphical data representing the ROS levels in complex-treated HeLa cells. All quantified values are expressed as mean ± SD (*n* = 3). Statistically significant values at ns < 0.033.

In the current case, the green fluorescence of all the treated cells appeared to be brighter than that of the control (Fig. 19a, b), indicating a rise in ROS levels (Fig. 19c, d). Moreover, using the plate reader test, ROS was quantified for each compound against HeLa cell line as displayed in (Fig. 19e). **TiC8** was found to have a fluorescence intensity of 92 a.u., greater than that of **TiC2** (69.6 a.u). As ROS-mediated DNA damage is the primary cause of cell death in HeLa cells, the complexes might enhance intracellular oxidative stress and induce apoptosis.

## Conclusion

A set of eight titanium(IV) complexes were synthesized and characterized using relevant spectroscopic techniques such as UV-vis, FT-IR, NMR^1^H^13^, C) and ESI-MS. Comparative in vitro interaction investigations of the complexes **TiC1–TiC8** with CT-DNA and BSA were conducted employing UV-vis absorption and fluorescence spectroscopy to assess their binding potential. Produced data revealed that complexes were attached to CT-DNA by groove binding and caused static quenching of the BSA. Further, molecular docking investigations authenticated the mode interaction of the complexes with DNA/BSA and corroborated with the spectral data. The experimental findings indicated the strong affinity of **TiC2** for DNA and **TiC8** for BSA, as consistent with the theoretical estimates. The type of group attached to the Ti(IV) metal centre that affects the in *vitro* activity of the complexes and was identified as the cause of the observed trend in the binding interactions and computational analyses. A panel of cancer cell lines, including HeLa (cervical) and MCF7 (breast) were tested for in vitro anticancer activity. The results of **TiC2** and **TiC8** showcased remarkable cytotoxicity with IC_**50**_ values for HeLa cell lines as **12.61 ± 1.2** and **14.77 ± 1.2 µM/uL** but the complexes were unable to exhibit cytotoxicity against the MCF7 cell line. Eventually, complexes **TiC2** and **TiC8** were subjected for further in vitro assays with HeLa cell lines i.e. dual staining by AO-EB, flow cytometry by PI staining and ROS by DCFH-DA staining to enable **TiC2** and **TiC8** exhibit notable potency as anticancer drugs due to their strong efficacy.

## Supplementary Information

Below is the link to the electronic supplementary material.

Supplementary Material 1

## Data Availability

Authors declare that the data supporting the findings of the present study are available within the paper as well as in its Supplementary Information files. Regarding the crystal structure of ligand [L1], CCDC deposition 2170513 is enclosed in the supplementary crystallographic data for this paper. The relevant crystal data could be obtained (free of charge) from www.ccdc.cam.ac.uk/data_request/cif or by sending an email to data_request@ccdc.cam.ac.uk or by contacting ‘The Cambridge Crystallographic Data Centre, 12 Union Road, Cambridge CB2 1EZ, UK; fax: +44 1223 336033’.

## References

[1] Kargar, H. et al. Ultrasound-based synthesis, SC-XRD, NMR, DFT, HSA of new Schiff bases derived from 2-aminopyridine: Experimental and theoretical studies. *J Mol Struct* 1233, (2021).

[2] Rauf, A. et al. Synthesis, physicochemical elucidation, biological screening and molecular Docking studies of a schiff base and its metal(II) complexes. *Arab. J. Chem.* **13**, 1130–1141 (2020).

[3] Aspinall, H. C. Chiral lanthanide complexes: coordination chemistry and applications. *Chem. Rev.* **102**, 1807–1850 (2002). 12059255 10.1021/cr010288q

[4] Ardakani, A. A., Kargar, H., Feizi, N. & Tahir, M. N. Synthesis, characterization, crystal structures and antibacterial activities of some schiff bases with N2O2 donor sets. *J. Iran. Chem. Soc.* **15**, 1495–1504 (2018).

[5] Kargar, H., Ardakani, A. A., Tahir, M. N., Ashfaq, M. & Munawar, K. S. Synthesis, spectral characterization, crystal structure and antibacterial activity of nickel(II), copper(II) and zinc(II) complexes containing ONNO donor schiff base ligands. *J. Mol. Struct.* **1233**, (2021).

[6] Shongwe, M. S. et al. Coordination versatility of tridentate pyridyl aroylhydrazones towards iron: tracking down the elusive aroylhydrazono-based ferric spin-crossover molecular materials. *Dalton Trans.* **41**, 2500–2514 (2012). 22216420 10.1039/c1dt11407g

[7] Hiremath, K. B., Manochkumar, J., Ramamoorthy, S. & Shivashankar, M. Studies on DNA/HSA binding properties of new triazole-based Imine functionalized derivatives using spectroscopic and computational methods. *Bioorg. Chem.* **162**, (2025). 10.1016/j.bioorg.2025.108608 40403495

[8] Haas, K. L. & Franz, K. J. Application of metal coordination chemistry to explore and manipulate cell biology. *Chem. Rev.* **109**, 4921–4960 (2009). 19715312 10.1021/cr900134a PMC2761982

[9] Hartinger, C. G. & Dyson, P. J. Bioorganometallic chemistry—from teaching paradigms to medicinal applications. *Chem. Soc. Rev.* **38**, 391–401 (2009). 19169456 10.1039/b707077m

[10] Raczuk, E., Dmochowska, B., Samaszko-Fiertek, J. & Madaj, J. Different schiff Bases—Structure, importance and classification. *Molecules Doi*. 10.3390/molecules27030787 (2022). 10.3390/molecules27030787 PMC8839460 35164049

[11] Gopalakrishnan, A. K., Angamaly, S. A. & Velayudhan, M. P. An insight into the biological properties of Imidazole-Based schiff bases: A review. *ChemistrySelect* **6**, 10918–10947. 10.1002/slct.202102619 (2021).

[12] Egekenze, R., Gultneh, Y. & Butcher, R. Catalysis of alkene epoxidation by manganese(II) and (III) complexes of both schiff base and reduced schiff base ligands utilizing environmentally benign H2O2. *Polyhedron* **144**, 198–209 (2018).

[13] Al Zoubi, W. & Ko, Y. G. Schiff base complexes and their versatile applications as catalysts in oxidation of organic compounds: part I. *Appl. Organomet. Chem.* **31**, (2017).

[14] De, S., Jain, A. & Barman, P. Recent advances in the catalytic applications of chiral Schiff-Base ligands and metal complexes in asymmetric organic transformations. *ChemistrySelect*10.1002/slct.202104334 (2022).

[15] Boulechfar, C. et al. Schiff bases and their metal complexes: A review on the history, synthesis, and applications. *Inorg. Chem. Commun. Doi*. 10.1016/j.inoche.2023.110451 (2023).

[16] Krátký, M. et al. Sulfadiazine salicylaldehyde-based schiff bases: synthesis, antimicrobial activity and cytotoxicity. *Molecules* **22**, (2017). 10.3390/molecules22091573 PMC6151383 28925956

[17] Parveen, S. Recent advances in anticancer ruthenium schiff base complexes. *Appl. Organomet. Chem.*10.1002/aoc.5687 (2020).

[18] Ammar, R. A., Alaghaz, A. N. M. A., Zayed, M. E. & Al-Bedair, L. A. Synthesis, spectroscopic, molecular structure, antioxidant, antimicrobial and antitumor behavior of Mn(II), Co(II), Ni(II), Cu(II) and Zn(II) complexes of O2N type tridentate chromone-2-carboxaldehyde schiff’s base ligand. *J. Mol. Struct.* **1141**, 368–381 (2017).

[19] Alorini, T. A., Al-Hakimi, A. N., El-Sayed Saeed, S., Alhamzi, E. H. L. & Albadri, A. E. A. E. Synthesis, characterization, and anticancer activity of some metal complexes with a new schiff base ligand. *Arabian J. Chem.* **15**, (2022). 10.1080/07391102.2023.2191725 36961125

[20] Ghosh, S. & Cisplatin The first metal based anticancer drug. *Bioorg. Chem.*10.1016/j.bioorg.2019.102925 (2019). 10.1016/j.bioorg.2019.102925 31003078

[21] Calvert, H. The clinical development of carboplatin – A personal perspective. *Inorganica Chim. Acta*10.1016/j.ica.2019.118987 (2019).

[22] O’Dowd, P. D., Sutcliffe, D. F. & Griffith, D. M. Oxaliplatin and its derivatives – An overview. *Coord. Chem. Rev.*10.1016/j.ccr.2023.215439 (2023).

[23] Imran, M., Ayub, W. & Butler, I. S. Zia-ur-Rehman. Photoactivated platinum-based anticancer drugs. *Coord. Chem. Rev.* **376**, 405–429. 10.1016/j.ccr.2018.08.009 (2018).

[24] Amable, L. Cisplatin resistance and opportunities for precision medicine. *Pharmacol. Res.* **106**, 27–36. 10.1016/j.phrs.2016.01.001 (2016). 26804248 10.1016/j.phrs.2016.01.001

[25] Argyriou, A. A. Updates on oxaliplatin-induced peripheral neurotoxicity (OXAIPN). *Toxics* **3**, 187–197 10.3390/toxics3020187 (2015). 10.3390/toxics3020187 PMC5634688 29056657

[26] Muhammad, N. & Guo, Z. Metal-based anticancer chemotherapeutic agents. *Curr. Opin. Chem. Biol.* **19** 144–153 10.1016/j.cbpa.2014.02.003 (2014). 10.1016/j.cbpa.2014.02.003 24608084

[27] Gibson, D. Platinum(IV) anticancer prodrugs-hypotheses and facts. *Dalton Trans.* **45**, 12983–12991. 10.1039/c6dt01414c (2016). 27214873 10.1039/c6dt01414c

[28] Oun, R., Moussa, Y. E. & Wheate, N. J. The side effects of platinum-based chemotherapy drugs: A review for chemists. *Dalton Trans.* **47**, 6645–6653. 10.1039/c8dt00838h (2018). 29632935 10.1039/c8dt00838h

[29] Bergamo, A. & Sava, G. Linking the future of anticancer metal-complexes to the therapy of tumour metastases. *Chem. Soc. Rev.* **44**, 8818–8835. 10.1039/c5cs00134j (2015). 25812154 10.1039/c5cs00134j

[30] Anthony, E. J. et al. Metallodrugs are unique: opportunities and challenges of discovery and development. *Chem. Sci.* **11**, 12888–12917 (2020). 34123239 10.1039/d0sc04082g PMC8163330

[31] Moreno-Alcántar, G., Picchetti, P. & Casini, A. Gold complexes in anticancer therapy: from new design principles to Particle-Based delivery systems. *Angewandte Chemie - Int. Ed. Doi*. 10.1002/anie.202218000 (2023). 10.1002/anie.202218000 36847211

[32] Međedović, M. et al. Synthesis, characterization, biomolecular interactions, molecular docking, and in vitro and in vivo anticancer activities of novel ruthenium(III) schiff base complexes. *J. Inorg. Biochem.* **248**, (2023). 10.1016/j.jinorgbio.2023.112363 37689038

[33] Bouché, M., Hognon, C., Grandemange, S., Monari, A. & Gros, P. C. Recent advances in iron-complexes as drug candidates for cancer therapy: reactivity, mechanism of action and metabolites. *Dalton Trans.* **49**, 11451–11466. 10.1039/d0dt02135k (2020). 32776052 10.1039/d0dt02135k

[34] Abedi, A., Lighvan, Z. M. & Ostad, S. N. Cytotoxicity and DNA/BSA binding ability of copper(II) complexes with dimethylbithiazole. *Monatsh Chem.* **147**, 1651–1658 (2016).

[35] Zhang, P. & Huang, H. Future potential of osmium complexes as anticancer drug candidates, photosensitizers and organelle-targeted probes. *Dalton Trans.* **47**, 14841–14854 (2018). 30325378 10.1039/c8dt03432j

[36] Bauer, E. B., Haase, A. A., Reich, R. M., Crans, D. C. & Kühn, F. E. Organometallic and coordination rhenium compounds and their potential in cancer therapy. *Coord. Chem. Rev.* **393**, 79–117. 10.1016/j.ccr.2019.04.014 (2019).

[37] Bruijnincx, P. C. A. & Sadler, P. J. Controlling platinum, ruthenium, and osmium reactivity for anticancer drug design. *Adv. Inorg. Chem.* **61**, 1–62. 10.1016/S0898-8838(09)00201-3 (2009). 21258628 10.1016/S0898-8838(09)00201-3 PMC3024542

[38] Meker, S. et al. Anti-proliferative activity of nano-formulated phenolato titanium(IV) complexes against cancer cells. *ChemMedChem* **9**, 1294–1298 (2014). 24677761 10.1002/cmdc.201400038

[39] Ceballos-Torres, J. et al. Anti-cancer applications of titanocene-functionalised nanostructured systems: an insight into cell death mechanisms. *Chem.---Eur. J.* **20**, 10811–10828 (2014). 24715574 10.1002/chem.201400300

[40] Fernández-Gallardo, J. et al. Heterometallic titanium-gold complexes inhibit renal cancer cells in vitro and in vivo. *Chem. Sci.* **6**, 5269–5283 (2015). 27213034 10.1039/c5sc01753j PMC4869729

[41] Glasner, H. & Tshuva, E. Y. C 1-symmetrical titanium(IV) complexes of Salan ligands with differently substituted aromatic rings: enhanced cytotoxic activity. *Inorg. Chem.* **53**, 3170–3176 (2014). 24588655 10.1021/ic500001j

[42] Musa, M. et al. Probing the Mechanism of Action of Bis-Phenolato Amine (ONO Donor Set) Titanium(IV) Anti-Cancer Agents. 10.1021/acs.jmedchem.3c01874 PMC10895680 38331433

[43] Lord, R. M. et al. β-Diketonate Titanium Compounds Exhibiting High In Vitro Activity and Specific DNA Base Binding. *ChemistrySelect* **1**, 6598–6605 (2016).

[44] Musa, M. et al. Probing the mechanism of action of Bis(phenolato) amine (ONO donor Set) Titanium(IV) anticancer agents. *J. Med. Chem.* **67**, 2732–2744 (2024). 38331433 10.1021/acs.jmedchem.3c01874 PMC10895680

[45] Cini, M., Bradshaw, T. D. & Woodward, S. Using titanium complexes to defeat cancer: the view from the shoulders of titans. *Chem. Soc. Rev.* **46**, 1040–1051. 10.1039/c6cs00860g (2017). 28124046 10.1039/c6cs00860g

[46] Miller, M., Braitbard, O., Hochman, J. & Tshuva, E. Y. Insights into molecular mechanism of action of Salan titanium(IV) complex with in vitro and in vivo anticancer activity. *J. Inorg. Biochem.* **163**, 250–257 (2016). 27090292 10.1016/j.jinorgbio.2016.04.007

[47] Manna, C. M., Armony, G. & Tshuva, E. Y. Unexpected influence of stereochemistry on the cytotoxicity of highly efficient Ti IV Salan complexes: new mechanistic insights. *Chem.---Eur. J.* **17**, 14094–14103 (2011). 22076809 10.1002/chem.201102017

[48] Kargar, H. et al. Titanium(IV) complex containing ONO-tridentate schiff base ligand: synthesis, crystal structure determination, Hirshfeld surface analysis, spectral characterization, theoretical and computational studies. *J. Mol. Struct.* **1241**, (2021).

[49] Thanigachalam, S. & Pathak, M. Bioactive O^N^O^ schiff base appended homoleptic titanium(iv) complexes: DFT, BSA/CT-DNA interactions, molecular Docking and antitumor activity against HeLa and A549 cell lines. *RSC Adv.* **14**, 13062–13082 (2024). 38655487 10.1039/d3ra08574k PMC11034360

[50] Shavit, M., Peri, D., Manna, C. M., Alexander, J. S. & Tshuva, E. Y. Active cytotoxic reagents based on non-metallocene non-diketonato well-defined C2-symmetrical titanium complexes of tetradentate bis(phenolato) ligands. *J. Am. Chem. Soc.* **129**, 12098–12099 (2007). 17877357 10.1021/ja0753086

[51] Eshkourfu, R. et al. Synthesis, characterization, cytotoxic activity and DNA binding properties of the novel dinuclear cobalt(III) complex with the condensation product of 2-acetylpyridine and malonic acid dihydrazide. *J. Inorg. Biochem.* **105**, 1196–1203 (2011). 21722616 10.1016/j.jinorgbio.2011.05.024

[52] Li, Y., Yang, Z. Y. & Wang, M. F. Synthesis, characterization, DNA binding properties and antioxidant activity of Ln(III) complexes with hesperetin-4-one-(benzoyl) hydrazone. *Eur. J. Med. Chem.* **44**, 4585–4595 (2009). 19615791 10.1016/j.ejmech.2009.06.027

[53] Winkler, D. Modelling topoisomerase i Inhibition by minor groove binders. *Bioorg. Med. Chem.* **19**, 1450–1457 (2011). 21273082 10.1016/j.bmc.2011.01.003

[54] Shuai, L., Wang, S., Zhang, L., Fu, B. & Zhou, X. Cationic porphyrins and analogues as new DNA topoisomerase I and II inhibitors. *Chem. Biodivers.* **6**, 827–837 (2009). 19551725 10.1002/cbdv.200800083

[55] Salih Aǧrtaş, M., Cabir, B. & Özdemir, S. Novel metal (II) phthalocyanines with 3,4,5-trimethoxybenzyloxy- substituents: synthesis, characterization, aggregation behaviour and antioxidant activity. *Dyes Pigm.* **96**, 152–157 (2013).

[56] Özel, A., Barut, B., Demirbaş, Ü. & Biyiklioglu, Z. Investigation of DNA binding, DNA photocleavage, topoisomerase i Inhibition and antioxidant activities of water soluble titanium(IV) phthalocyanine compounds. *J. Photochem. Photobiol B*. **157**, 32–38 (2016). 26882290 10.1016/j.jphotobiol.2016.02.005

[57] Fasano, M. et al. The extraordinary ligand binding properties of human serum albumin. *IUBMB Life*. **57**, 787–796. 10.1080/15216540500404093 (2005). 16393781 10.1080/15216540500404093

[58] Topală, T., Bodoki, A., Oprean, L. & Oprean, R. Bovine serum albumin interactions with metal complexes. *Clujul Med.* **87**, 5 (2014). 10.15386/cjmed-357 PMC4620676 26528027

[59] Singh, A., Priya Gogoi, H. & Barman, P. Comparative study of palladium(II) complexes bearing tridentate ONS and NNS schiff base ligands: synthesis, characterization, DFT calculation, DNA binding, bioactivities, catalytic activity, and molecular docking. *Polyhedron* **221**, (2022).

[60] Kargar, H. et al. Novel copper(II) and zinc(II) complexes of halogenated bidentate N,O-donor schiff base ligands: synthesis, characterization, crystal structures, DNA binding, molecular docking, DFT and TD-DFT computational studies. *Inorganica Chim. Acta* **514**, (2021).

[61] Samajpaty, S. Solubility of Nifedipine by shake flask UV-Spectrometry; review of safety concerns in pregnancy. *Biomedical Pharmacol. J.* **14**, 1823–1829 (2021).

[62] Aman, F. et al. Anticancer Ruthenium(n6- p -cymene) complexes of nonsteroidal anti-inflammatory drug derivatives. *Organometallics* **33**, 5546–5553 (2014).

[63] Kubanik, M., Holtkamp, H., Söhnel, T., Jamieson, S. M. F. & Hartinger, C. G. Impact of the halogen substitution pattern on the biological activity of organoruthenium 8-Hydroxyquinoline anticancer agents. *Organometallics* **34**, 5658–5668 (2015).

[64] Andrés, A. et al. Setup and validation of shake-flask procedures for the determination of partition coefficients (log D) from low drug amounts. *Eur. J. Pharm. Sci.* **76**, 181–191. 10.1016/j.ejps.2015.05.008 (2015). 25968358 10.1016/j.ejps.2015.05.008

[65] Sirajuddin, M., Ali, S. & Badshah, A. Drug-DNA interactions and their study by UV-Visible, fluorescence spectroscopies and cyclic voltametry. *J. Photochem. Photobiol. B: Biol.* **124**, 1–19 10.1016/j.jphotobiol.2013.03.013 (2013). 10.1016/j.jphotobiol.2013.03.013 23648795

[66] Phadte, A. A., Banerjee, S., Mate, N. A. & Banerjee, A. Spectroscopic and viscometric determination of DNA-binding modes of some bioactive dibenzodioxins and phenazines. *Biochem. Biophys. Rep.* **18**, (2019). 10.1016/j.bbrep.2019.100629 PMC6449707 30993216

[67] Baguley, B. C. & Le Bret, M. Quenching of DNA-Ethidium fluorescence by amsacrine and other antitumor agents: A possible Electron-Transfer effect?? *Biochemistry* **23** (1984). 10.1021/bi00300a022 6546881

[68] Karami, K. et al. Novel fluorescence palladium-alkoxime complexes: synthesis, characterization, DNA/BSA spectroscopic and Docking studies, evaluation of cytotoxicity and DNA cleavage mechanism. *J. Mol. Struct.* **1206**, (2020).

[69] Maiti, S. K., Kalita, M., Singh, A., Deka, J. & Barman, P. Investigation of DNA binding and bioactivities of thioether containing schiff base Copper(II), Cobalt(II) and Palladium(II) complexes: synthesis, characterization, spectrochemical study, viscosity measurement. *Polyhedron* **184**, (2020).

[70] Heidari, A., Dehghanian, E. & Ahmar, H. Investigation of DNA binding of newly designed Zn (II) complexes with N-N and O-O donor ligands as potential antioxidants: spectroscopic, electrochemical, and molecular Docking studies. *Appl. Organomet. Chem.* **39**, (2025).

[71] Ramzan, S. et al. Structural characterization, DNA binding study, antioxidant potential and antitumor activity of diorganotin(IV) complexes against human breast cancer cell line MDA-MB-231. *J. Organomet. Chem.* **990**, (2023).

[72] Shah, A. et al. Characterization and DNA binding studies of unexplored Imidazolidines by electronic absorption spectroscopy and Cyclic voltammetry. *J. Photochem. Photobiol. B*. **120**, 90–97 (2013). 23474470 10.1016/j.jphotobiol.2012.12.015

[73] Qadeer, L. et al. Synthesis, spectral Elucidation and DNA binding studies of cadmium(II) carboxylates with nitrogen donor heteroligands. *Inorg. Chem. Commun.* **168**, (2024).

[74] Dustkami, M. & Mansouri-Torshizi, H. Refolding and unfolding of CT-DNA by newly designed Pd(II) complexes. Their synthesis, characterization and antitumor effects. *Int. J. Biol. Macromol.* **99**, 319–334 (2017). 28249766 10.1016/j.ijbiomac.2017.02.063

[75] Mahendiran, D., Kumar, R. S., Viswanathan, V., Velmurugan, D. & Rahiman, A. K. Targeting of DNA molecules, BSA/: C -Met tyrosine kinase receptors and anti-proliferative activity of bis(terpyridine)copper(II) complexes. *Dalton Trans.* **45**, 7794–7814 (2016). 27063595 10.1039/c5dt03831f

[76] Gao, C. Y. et al. Synthesis, characterization, DNA binding and cleavage, BSA interaction and anticancer activity of dinuclear zinc complexes. *Dalton Trans.* **41**, 12220–12232 (2012). 22930131 10.1039/c2dt31306e

[77] Samari, F., Hemmateenejad, B., Shamsipur, M., Rashidi, M. & Samouei, H. Affinity of two novel five-coordinated anticancer Pt(II) complexes to human and bovine serum albumins: A spectroscopic approach. *Inorg. Chem.* **51**, 3454–3464 (2012). 22364149 10.1021/ic202141g

[78] Zarei, L., Asadi, Z., Samolova, E., Dusek, M. & Amirghofran, Z. Pyrazolate as bridging ligand in stabilization of self-assemble Cu(II) schiff base complexes: synthesis, structural investigations, dna/protein (BSA) binding and growth inhibitory effects on the MCF7, CT-26, MDA-MB-231 cell lines. *Inorg. Chim. Acta* **509**, (2020).

[79] Paitandi, R. P. et al. Interaction of ferrocene appended Ru(II), Rh(III) and Ir(III) Dipyrrinato complexes with dna/protein, molecular Docking and antitumor activity. *Eur. J. Med. Chem.* **84**, 17–29 (2014). 25014746 10.1016/j.ejmech.2014.06.052

[80] Jana, S. K., Seth, S. K., Puschmann, H., Hossain, M. & Dalai, S. Synthesis and X-ray structure of a new zinc(ii) coordination polymer: interaction with DNA and double stranded RNA and Elucidation of the molecular aspects of the binding to bovine serum albumin. *RSC Adv.* **4**, 57855–57868 (2014).

[81] Karami, K. et al. Synthesis of a novel trinuclear palladium complex: the influence of an oxime chelate ligand on biological evaluation towards double-strand DNA, BSA protein and molecular modeling studies. *RSC Adv.* **6**, 78424–78435 (2016).

[82] Bashir, M., Yousuf, I. & Prakash Prasad, C. Mixed Ni(II) and Co(II) complexes of Nalidixic acid drug: synthesis, characterization, DNA/BSA binding profile and in vitro cytotoxic evaluation against MDA-MB-231 and HepG2 cancer cell lines. *Spectrochim. Acta Mol. Biomol. Spectrosc.* **271**, (2022). 10.1016/j.saa.2022.120910 35077983

[83] Kalantari, R. & Asadi, Z. DNA/BSA binding of a new Oxovanadium (IV) complex of glycylglycine derivative schiff base ligand. *J. Mol. Struct.* **1219**, (2020).

[84] Annaraj, B., Balakrishnan, C., Neelakantan, M. A. & Synthesis Structure information, DNA/BSA binding affinity and in vitro cytotoxic studies of mixed ligand copper(II) complexes containing a phenylalanine derivative and diimine co-ligands. *J. Photochem. Photobiol B*. **160**, 278–291 (2016). 27155593 10.1016/j.jphotobiol.2016.04.021

[85] Kargar, H. et al. Synthesis, characterization, crystal structures, DFT, TD-DFT, molecular Docking and DNA binding studies of novel copper(II) and zinc(II) complexes bearing halogenated bidentate N,O-donor schiff base ligands. *Polyhedron* **195**, (2021).

[86] Deswal, Y. et al. Cobalt(II), nickel(II), copper(II) and zinc(II) complexes of thiadiazole based schiff base ligands: synthesis, structural characterization, DFT, antidiabetic and molecular Docking studies. *J. Mol. Struct.* **1253**, (2022).

[87] Abu-Dief, A. M. et al. Synthesis and intensive characterization for novel Zn(II), Pd(II), Cr(III) and VO(II)-Schiff base complexes; DNA-interaction, DFT, drug-likeness and molecular Docking studies. *J. Mol. Struct.* **1242**, (2021).

[88] Frisch, M. J. et al. Gaussian 09, Revision D. 01. Gaussian 09, Revision D. Inc., Wallingford CT (2009).

[89] Soleimani Amiri, S., Makarem, S., Ahmar, H. & Ashenagar, S. Theoretical studies and spectroscopic characterization of novel 4-methyl-5-((5-phenyl-1,3,4-oxadiazol-2-yl)thio)benzene-1,2-diol. *J. Mol. Struct.* **1119**, 18–24 (2016).

[90] Govindarajan, M., Periandy, S. & Carthigayen, K. FT-IR and FT-Raman spectra, thermo dynamical behavior, HOMO and LUMO, UV, NLO properties, computed frequency Estimation analysis and electronic structure calculations on α-bromotoluene. *Spectrochim Acta Mol. Biomol. Spectrosc.* **97**, 411–422 (2012). 10.1016/j.saa.2012.06.028 22820044

[91] Padmanabhan, J., Parthasarathi, R., Subramanian, V. & Chattaraj, P. K. Electrophilicity-based charge transfer descriptor. *J. Phys. Chem. A*. **111**, 1358–1361 (2007). 17256919 10.1021/jp0649549

[92] Mahadevi, P., Sumathi, S., Metha, A. & Singh, J. Synthesis, spectral, antioxidant, in vitro cytotoxicity activity and thermal analysis of schiff base metal complexes with 2,2′-Bipyridine-4,4′-dicarboxylic acid as co-ligand. *J. Mol. Struct.* **1268**, (2022).

[93] Ali, A. et al. Ligand substituent effect on the cytotoxicity activity of two new copper(ii) complexes bearing 8-hydroxyquinoline derivatives: validated by MTT assay and apoptosis in MCF-7 cancer cell line (human breast cancer). *RSC Adv.* **11**, 14362–14373 (2021). 35423979 10.1039/d1ra00172h PMC8697721

[94] Li, G. Y. et al. Synthesis, crystal structure, DNA interaction and anticancer activity of tridentate copper(II) complexes. *J. Inorg. Biochem.* **119**, 43–53 (2013). 23186647 10.1016/j.jinorgbio.2012.09.019

[95] Atale, N., Gupta, S., Yadav, U. C. S. & Rani, V. Cell-death assessment by fluorescent and nonfluorescent cytosolic and nuclear staining techniques. *J. Microsc*. **255**, 7–19 (2014). 24831993 10.1111/jmi.12133

[96] Miller, J. S. & Quarles, J. M. Flow cytometric identification of microorganisms by dual staining with FITC and PI. *Cytometry* **11**, 667–675 (1990). 1696535 10.1002/cyto.990110603

[97] Tian, S. et al. Ruthenium(II) polypyridyl complexes inhibit tumor growth through stimulating immune system to increase CD8 + T cell. *Eur. J. Med. Chem.* **289**, (2025). 10.1016/j.ejmech.2025.117470 40054298

[98] Arunachalam, A., Rengan, R., Umapathy, D. & Arockiam, A. J. V. Impact of biphenyl Benzhydrazone-Incorporatedð Arene Ru(II) complexes on cytotoxicity and the cancer cell death mechanism. *Organometallics* **41**, 2474–2486 (2022).

