# Supplementary material for: In-vitro antiproliferative evaluation of newly synthesized titanium(IV) metallacyclic complexes on HeLa and MCF7 cell lines
Source: Sci Rep. 2025 Aug 8;15:29066. doi: 10.1038/s41598-025-13995-0 (PMC12334760; doi:10.1038/s41598-025-13995-0)
Supplement: Supplementary file 1 — Supplementary Material 1 [file 41598_2025_13995_MOESM1_ESM.docx]

**Supporting Information**

***In-vitro* antiproliferative evaluation of newly synthesized titanium(IV) metallacyclic complexes on HeLa and MCF7 cell lines**

Shivabasayya V Salimath*^a^*, Kavita B Hiremath*^a^*, Sathish Thanigachalam*^a^*, Arjita Ghosh*^b^*, Selva Kumar Ramasamy*^c^*, Anbalagan Moorthy *^b^*, S K Ashokkumar*^a^*, Shivashankar M*^a^* and Madhvesh Pathak*^a^**

*^a^Department of Chemistry, School of Advanced Sciences, Vellore Institute of Technology (VIT), Vellore, Tamil Nadu, India.*

*^b^Department of Integrative Biology, School of Bioscience and Technology (SBST), Vellore Institute of Technology* *(VIT), Vellore, Tamil Nadu, India.*

*^c^Department of Chemistry, M.M. Engineering College, Maharishi Markandeshwar (Deemed to be University), Mullana, Ambala, Haryana, India.*

**Email:* [*madhveshpathak@vit.ac.in*](mailto:madhveshpathak@vit.ac.in) *ORCID iD*:* [*https://orcid.org/0000-0002-1567-6519*](https://orcid.org/0000-0002-1567-6519)

| Figures and tables | Title | Page.No |
| --- | --- | --- |
| Fig.S1-S6 | **NMR, FTIR, UV and Single crystal data of Ligand** | **3-5** |
| Fig.S7-S46 | **NMR, FTIR and ESI‐MS spectral data of Complexes 1-8** | **6-25** |
| Fig.S47-48 | **UV-Vis Spectra Fluorescence spectra of the complexes in DMSO: H_2_O (1:9)** | **26** |
| Fig.S49-50 | **Stability of complexes in** **1:9 DMSO: H_2_O and GSH medium** | **27-29** |
| Fig.S51 | **Lipophilicity; UV-Vis study of complexes in octanol and water** | **30-31** |
| Fig.S52 | **UV DNA Binding studies and Linear plots of complexes** | **32-34** |
| Fig.S53 | **EtBr Fluorescence quenching studies of DNA binding, Stern-Volmer plots of I_0_/I vs. complex and Scatchard plot of log([I_0_-I]/I) vs log [complex]** | **35-38** |
| Fig.S54 | **Viscosity studies of Complexes** | **39** |
| Fig.S55 | **Cyclic Voltammograms of Ti(IV) complexes with CT-DNA** | **39-40** |
| Table.S1. | **Oxidation and reduction peaks obtained from Cyclic Voltammograms of Ti(IV) complexes** | **40** |
| Fig.S56 | **BSA Fluorescence binding studies ,Stern-Volmer plots of I_0_/I vs complex and Scatchard plot of log([I_0_-I]/I) vs log [complex]** | **40-44** |
| Fig.S57 | **Synchronous spectra of BSA with increasing concentration of Ti(IV) complexes and Stern-Volmer plots of I_0_/I vs complex for Synchronous fluorescence spectra of complexes and BSA at Δλ=15 nm at 298 K** | **45-47** |
| Fig.S58 | **Synchronous spectra of BSA with increasing concentration of Ti(IV) complexes and Stern-Volmer plots of I_0_/I vs complex for Synchronous fluorescence spectra of complexes and BSA at Δλ=60 nm at 298 K** | **47-50** |
| Fig.S59 | **Site marker fluorescence quenching studies of BSA+Ibuprofin with an increase in the concentration of Ti(IV) complexes and Scatchard plot of log([I_0_-I]/I) vs log [complex]** | **51-53** |
| Fig.S60 | **Site marker fluorescence quenching studies of BSA+Warfarin with an increase in the concentration of Ti(IV) complexes and Scatchard plot of log([I_0_-I]/I) vs log [complex]** | **54-56** |
| Fig.S61 -68 | **Molecular docking of Ti (IV) complexes with BSA** | **57-60** |
| Fig.S69 | **Molecular docking of Ti (IV) complexes with DNA** | **61** |
| Table S2 -S3 | **Tabulations of DFT studies** | **62** |
| Fig.S70 | **DPPH assay** | **63** |
| Fig.S71-73 | **MTT** **assay** | **64-66** |
| Table S4 -S10 | **Crystal data of ligand L1** | **67-70** |
|  | **References** | **71** |

**
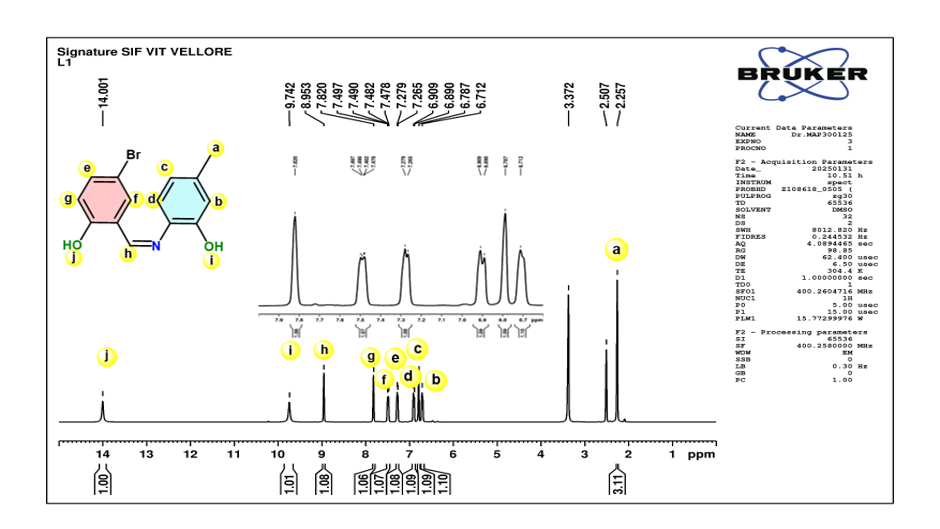
**

**
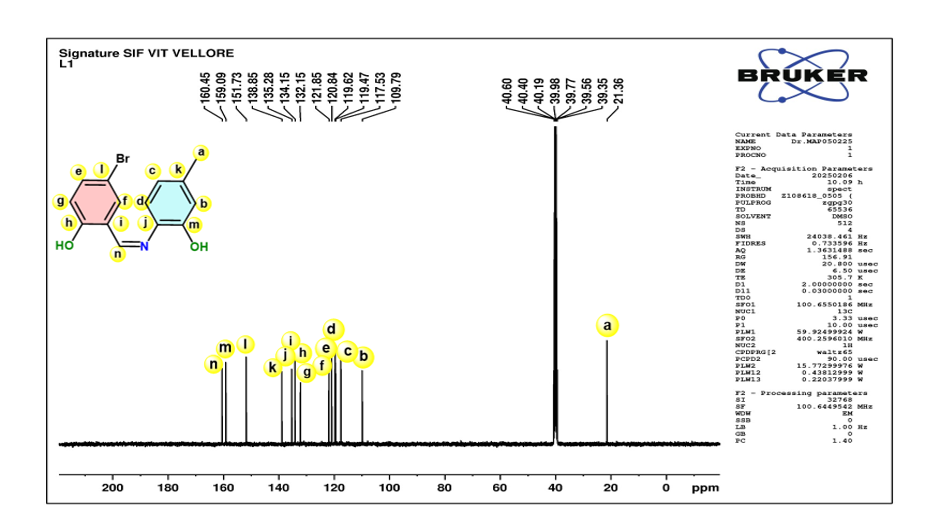
Figure.S1. ^1^H NMR spectrum of L1 (400 MHz, DMSO-d_6_)**

**Figure. S2. ^13^C NMR spectrum of L1 (400 MHz, DMSO-d_6_)**


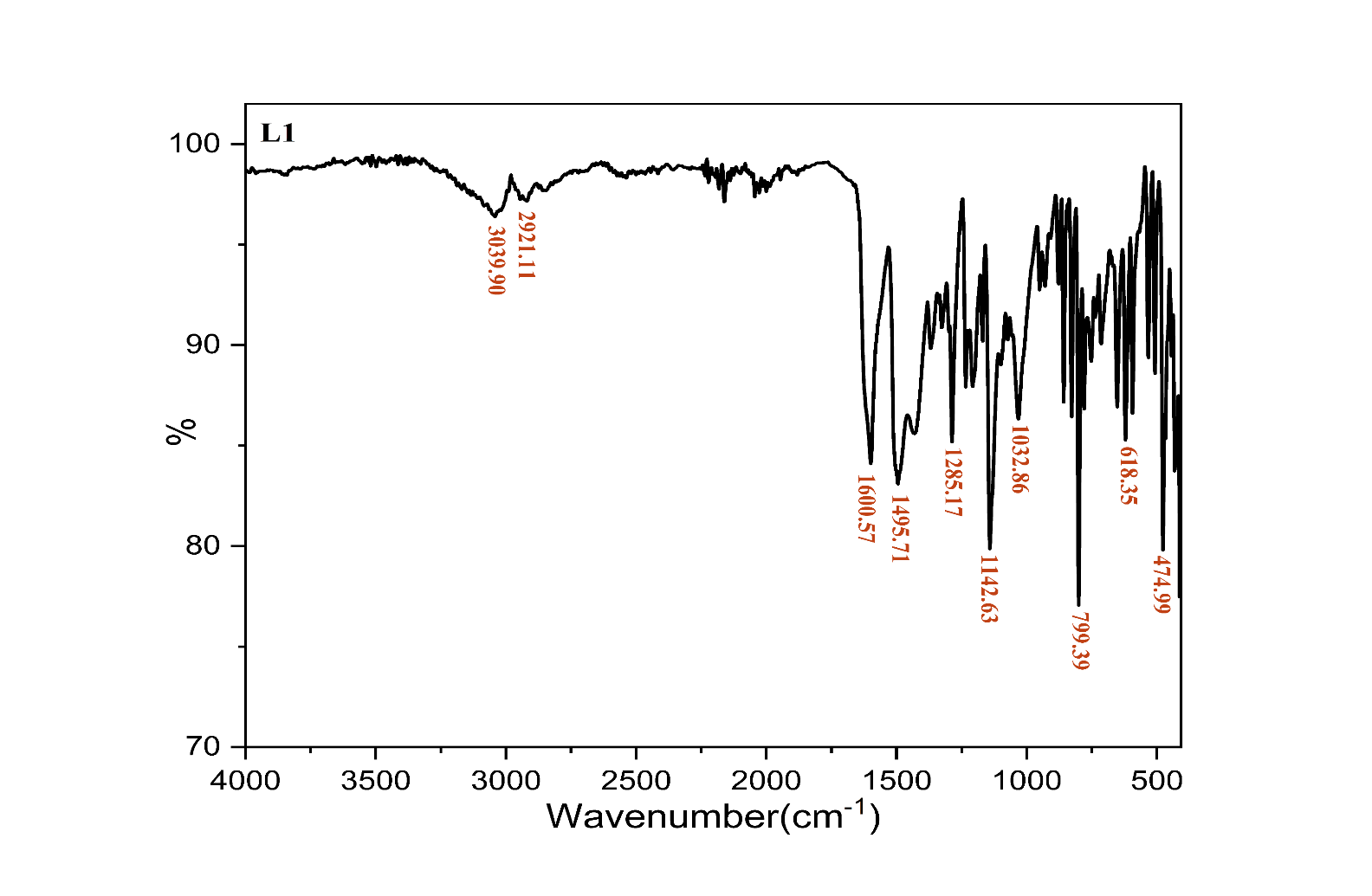


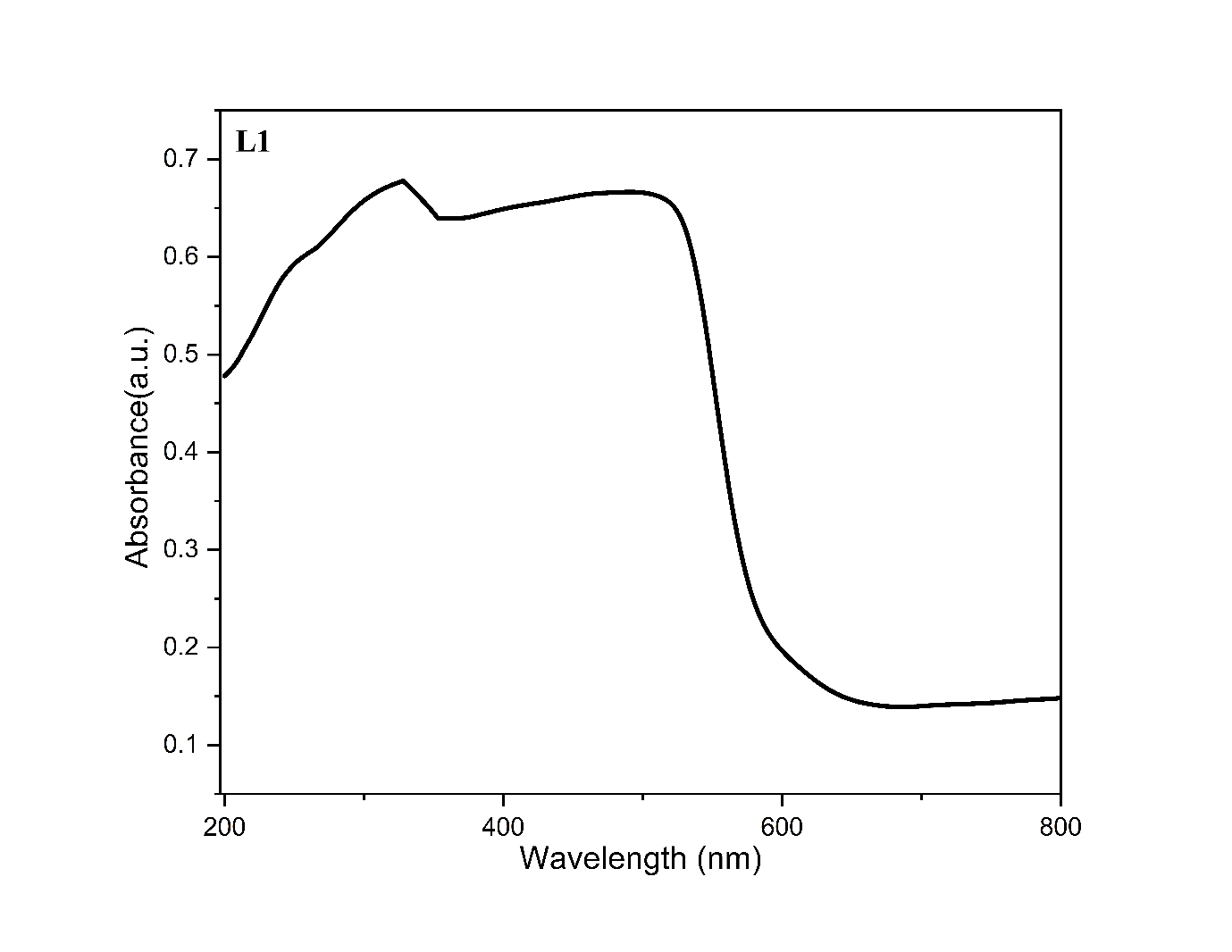
**Figure.S3****.** **FT- IR spectrum of L1**

**Figure.S4. UV spectrum of L1**


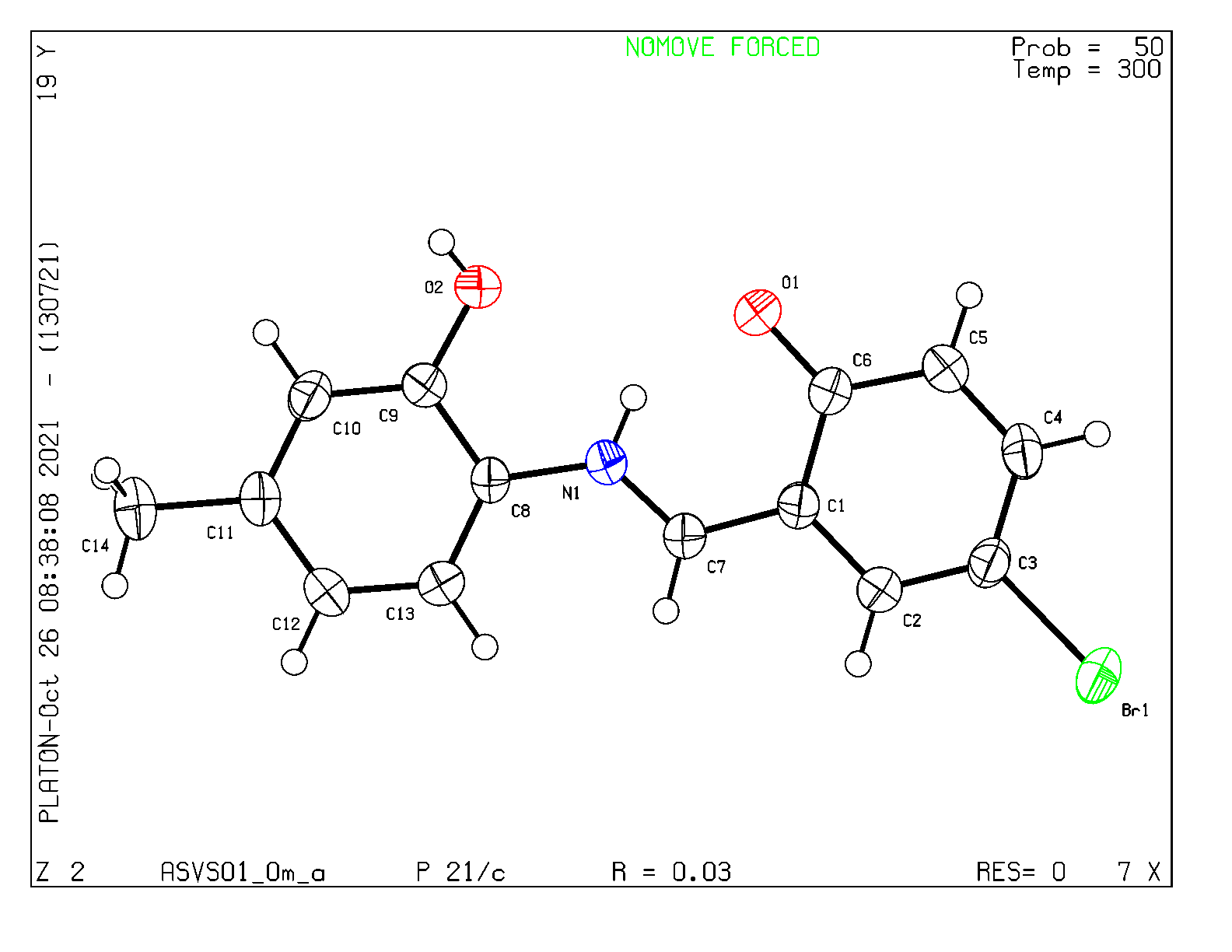


**Figure.S5. ORTEP diagram of ASVS01[L1]** **(CCDC Deposition Number: 2170513)**


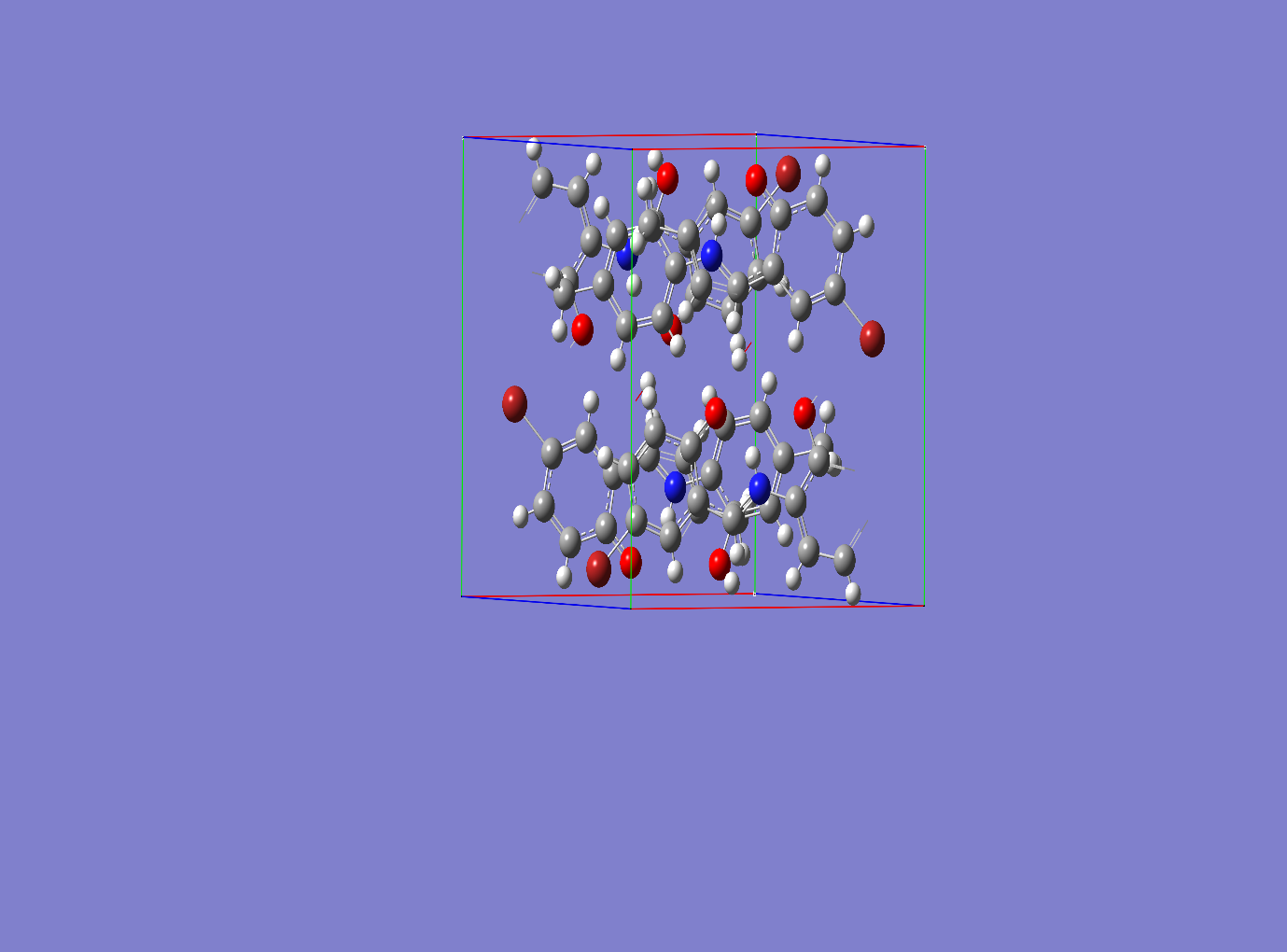

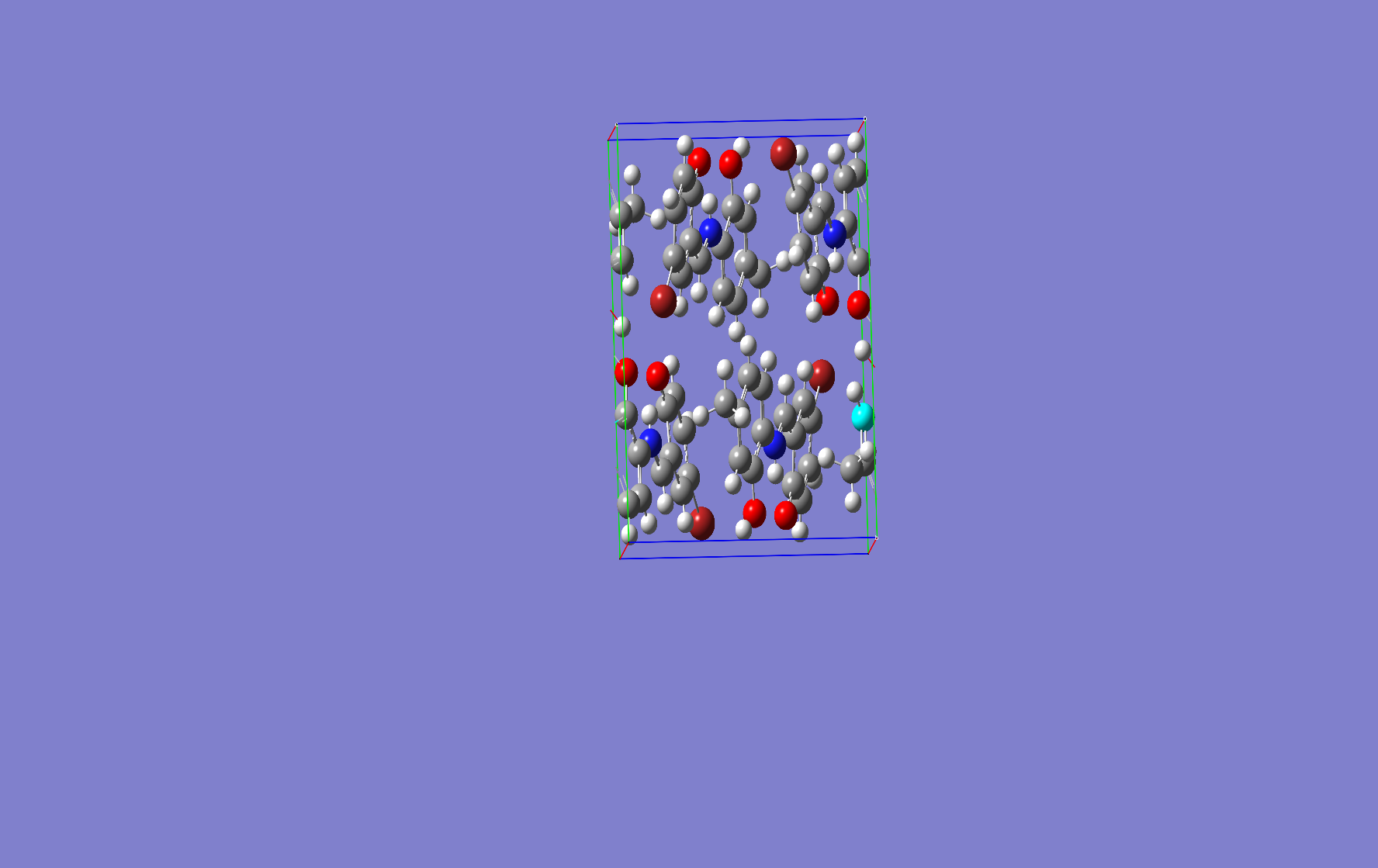

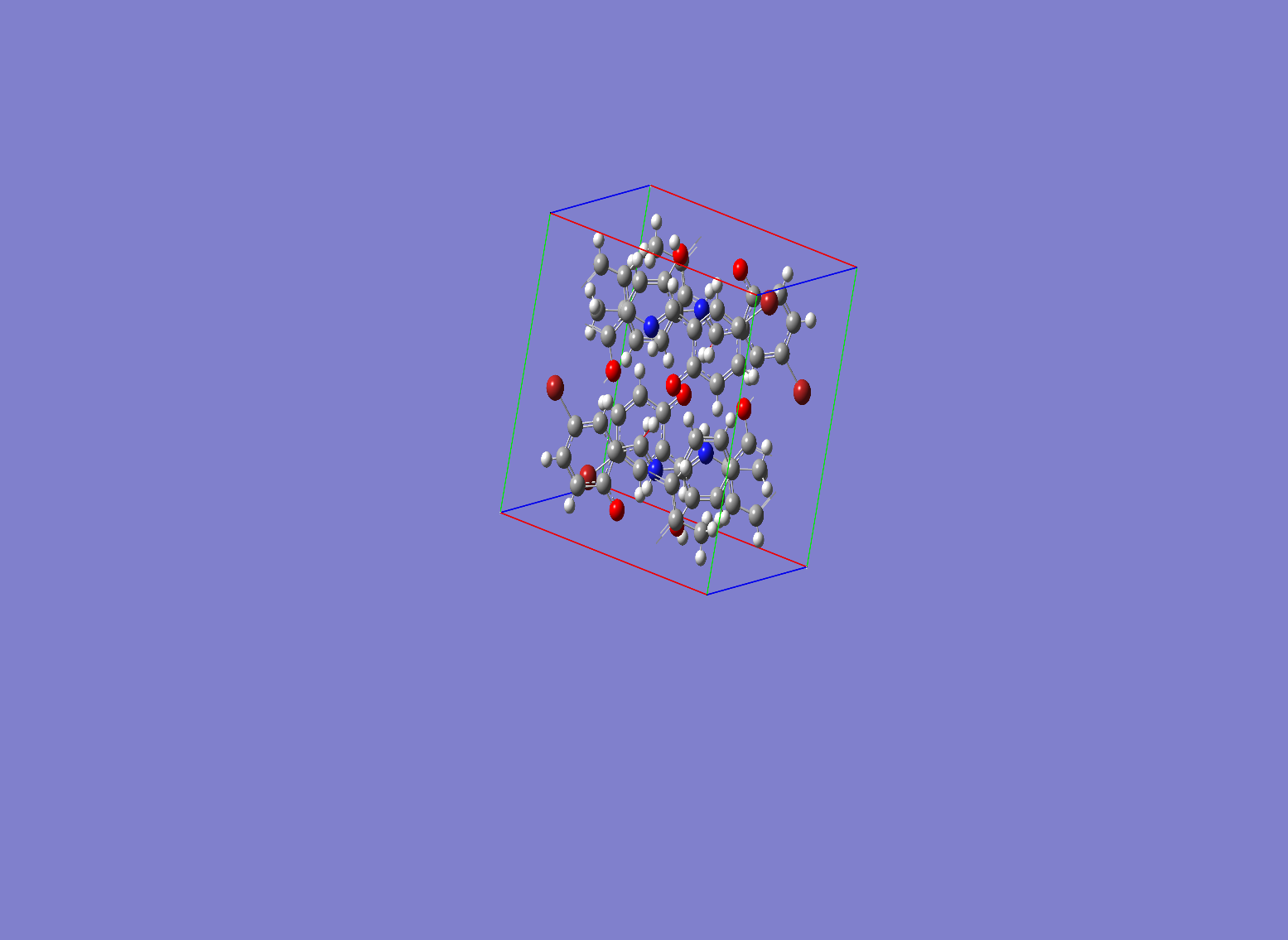


**Figure.S6. Packing diagram and unit cell of ASVS01 as obtained by X-ray diffractometric analysis.**


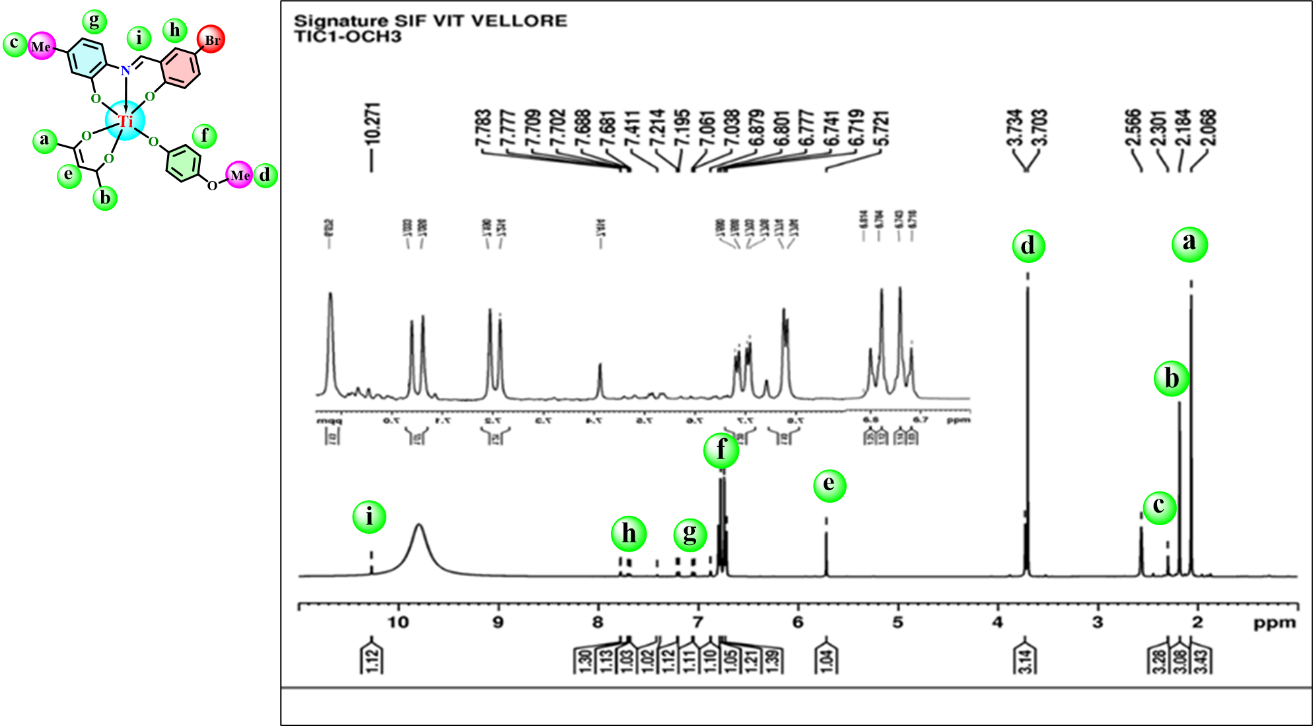


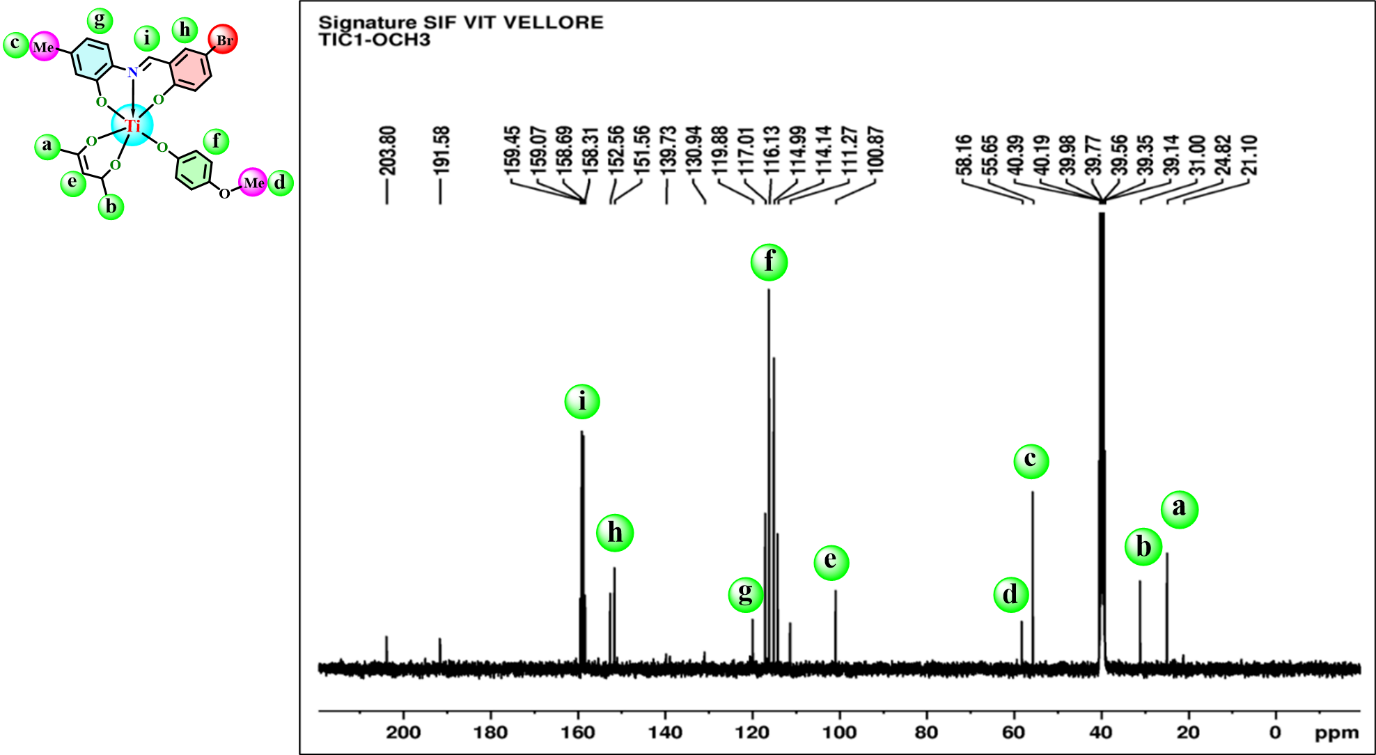
**Figure.S7.** **^1^H NMR spectrum of TiC1(400 MHz, DMSO-d_6_)**

**Figure.S8. ^13^C NMR spectrum of TiC1 (400 MHz, DMSO-d_6_)**


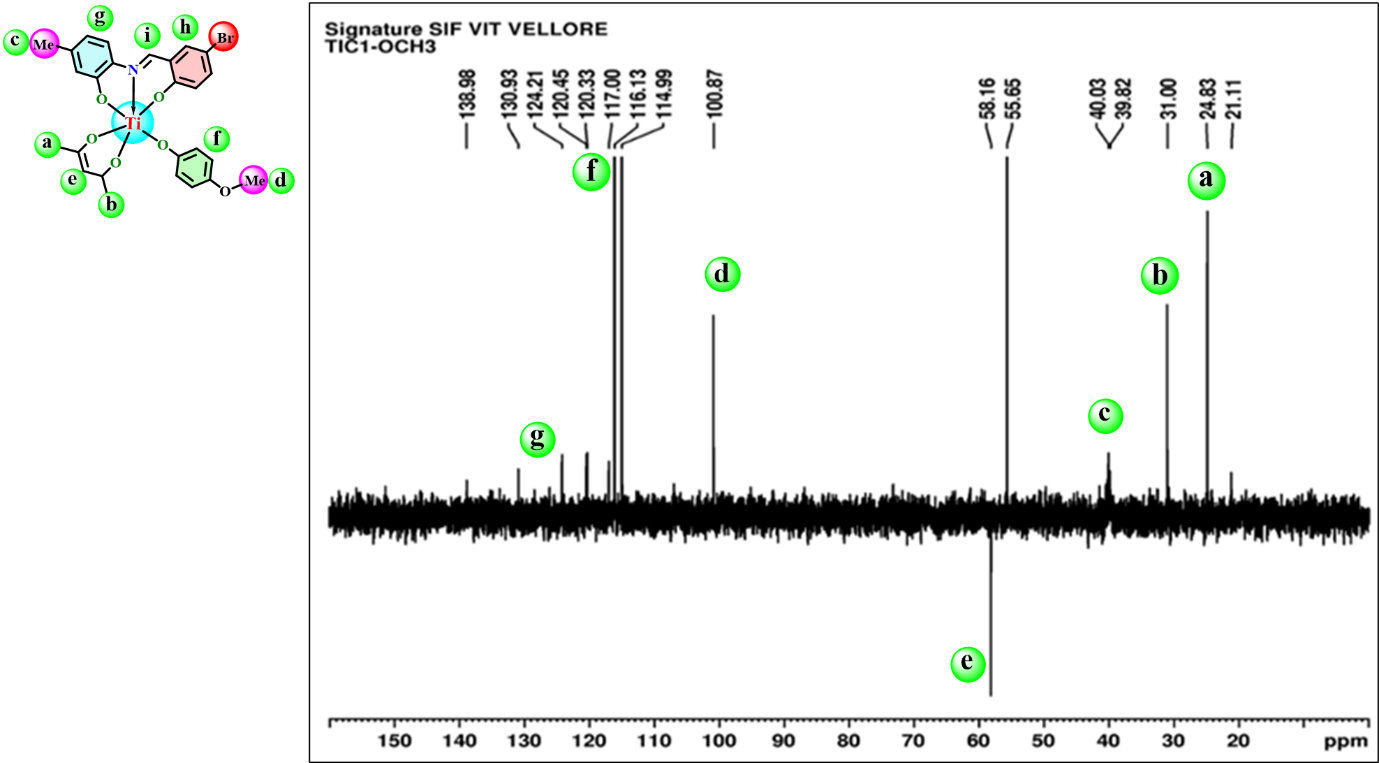


**Figure.S9. DEPT-135 NMR spectrum of TiC1 (400 MHz, DMSO-d_6_)**


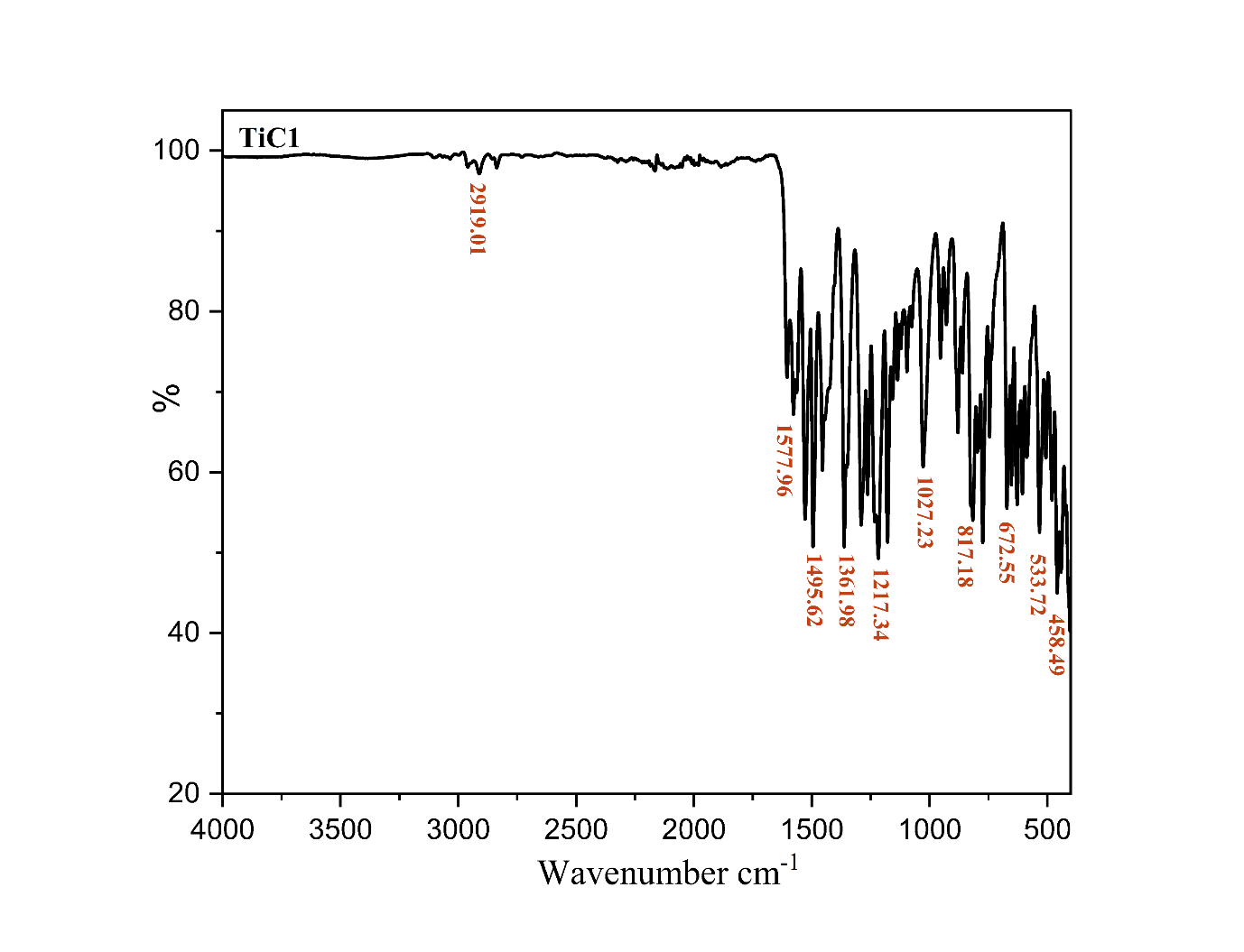
**Figure.S10. FT- IR spectrum of** **TiC1**


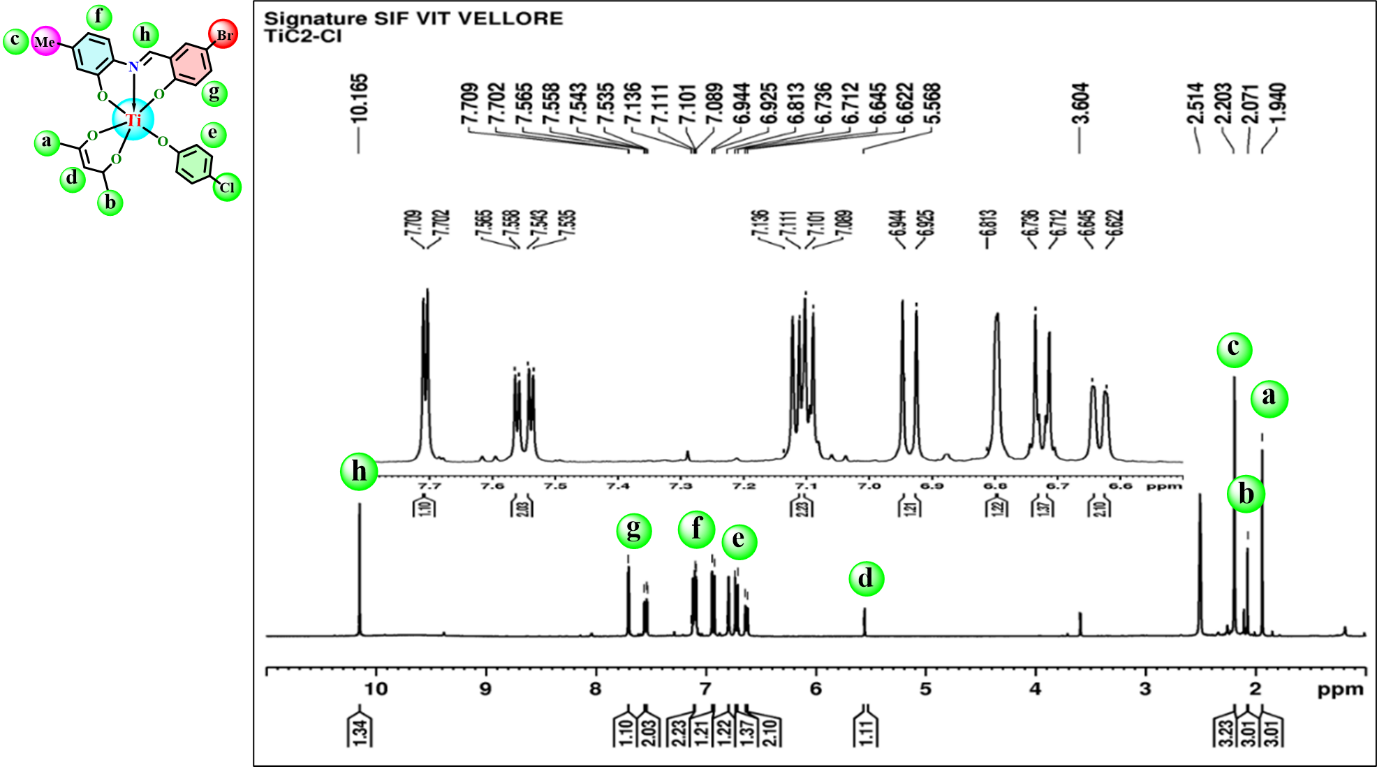

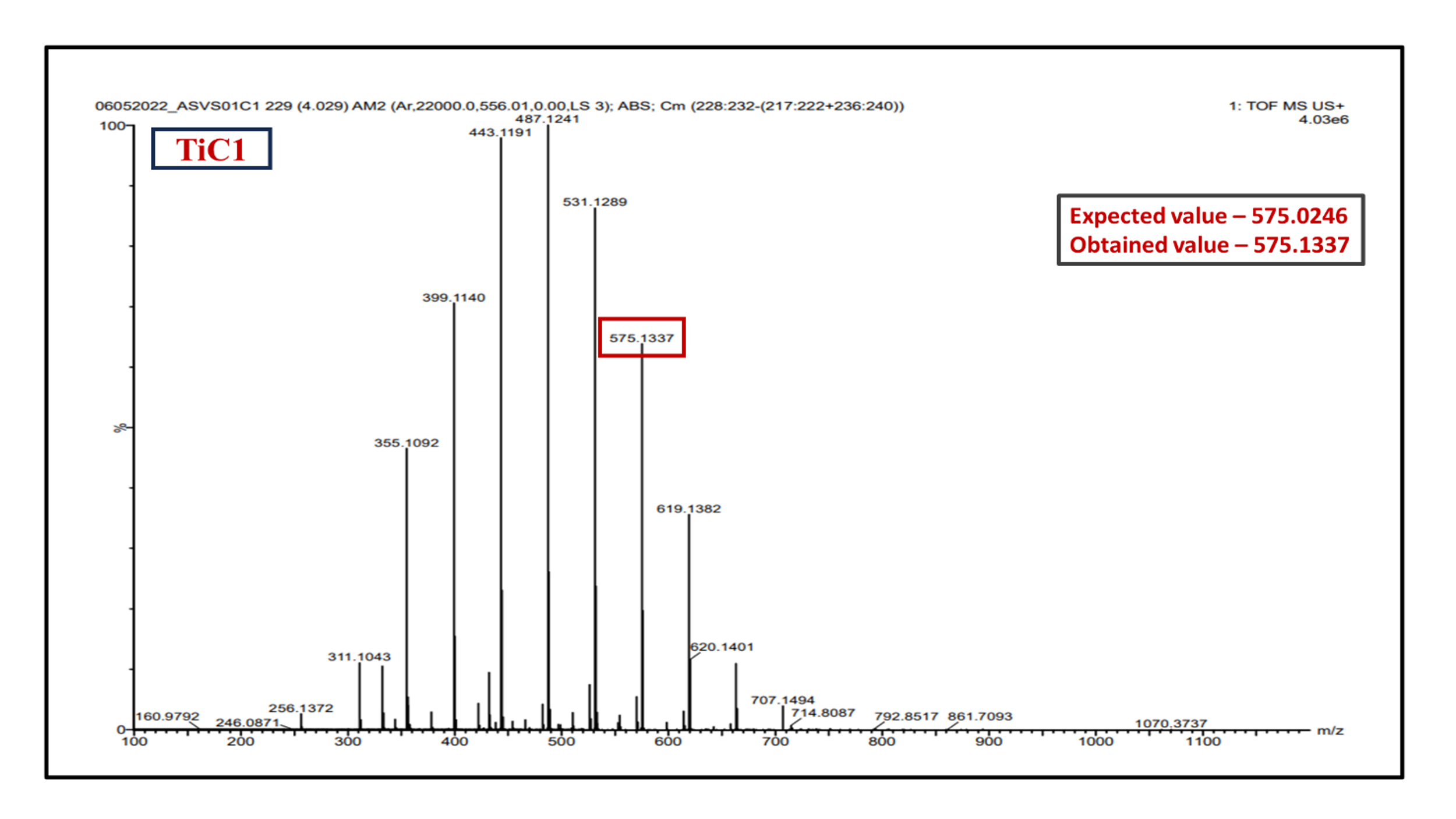
**Figure.S11.** **Electrospray ionization ESI‐MS (m/z) spectrum of TiC1**

**Figure.S12. ^1^H NMR spectrum of TiC2(400 MHz, DMSO-d_6_)**

**
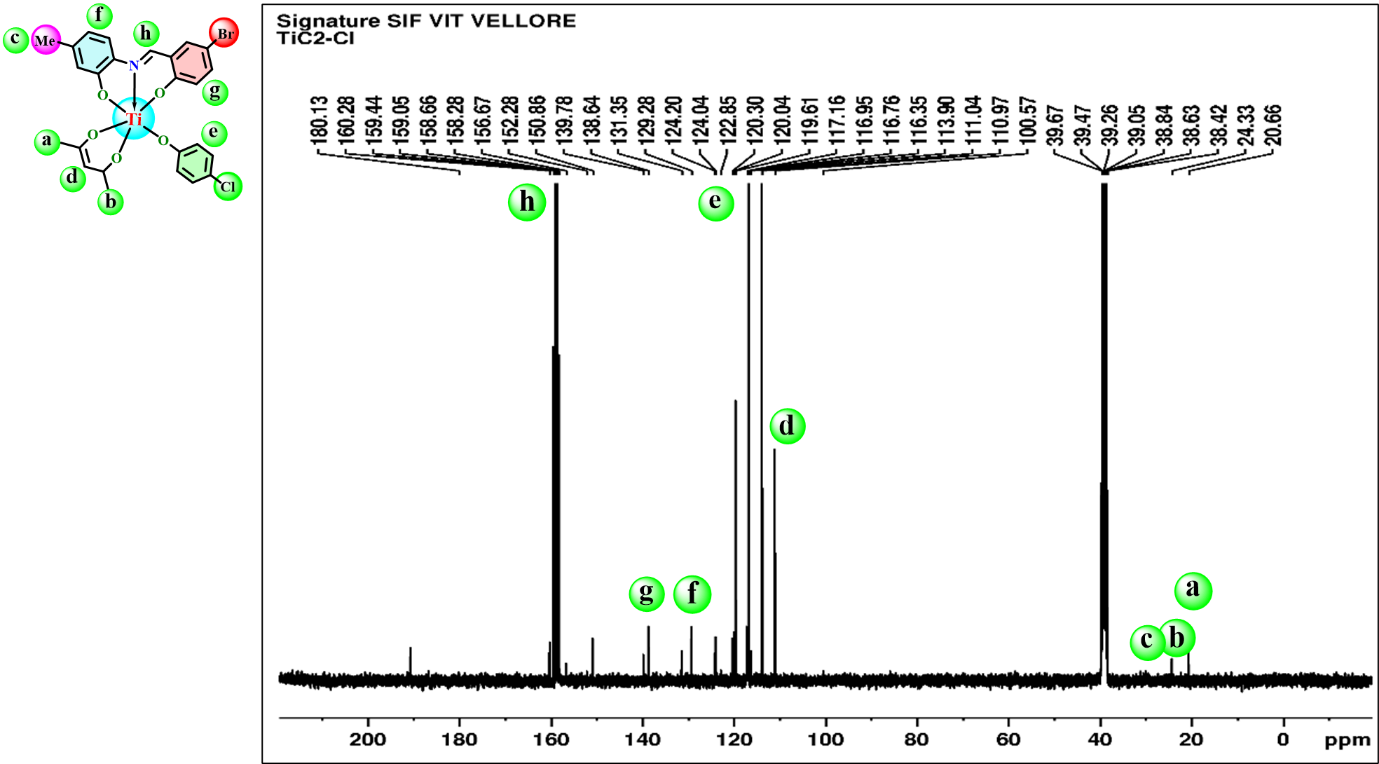
**

**Figure.S13. ^13^C NMR spectrum of TiC1(400 MHz, DMSO-d_6_)**


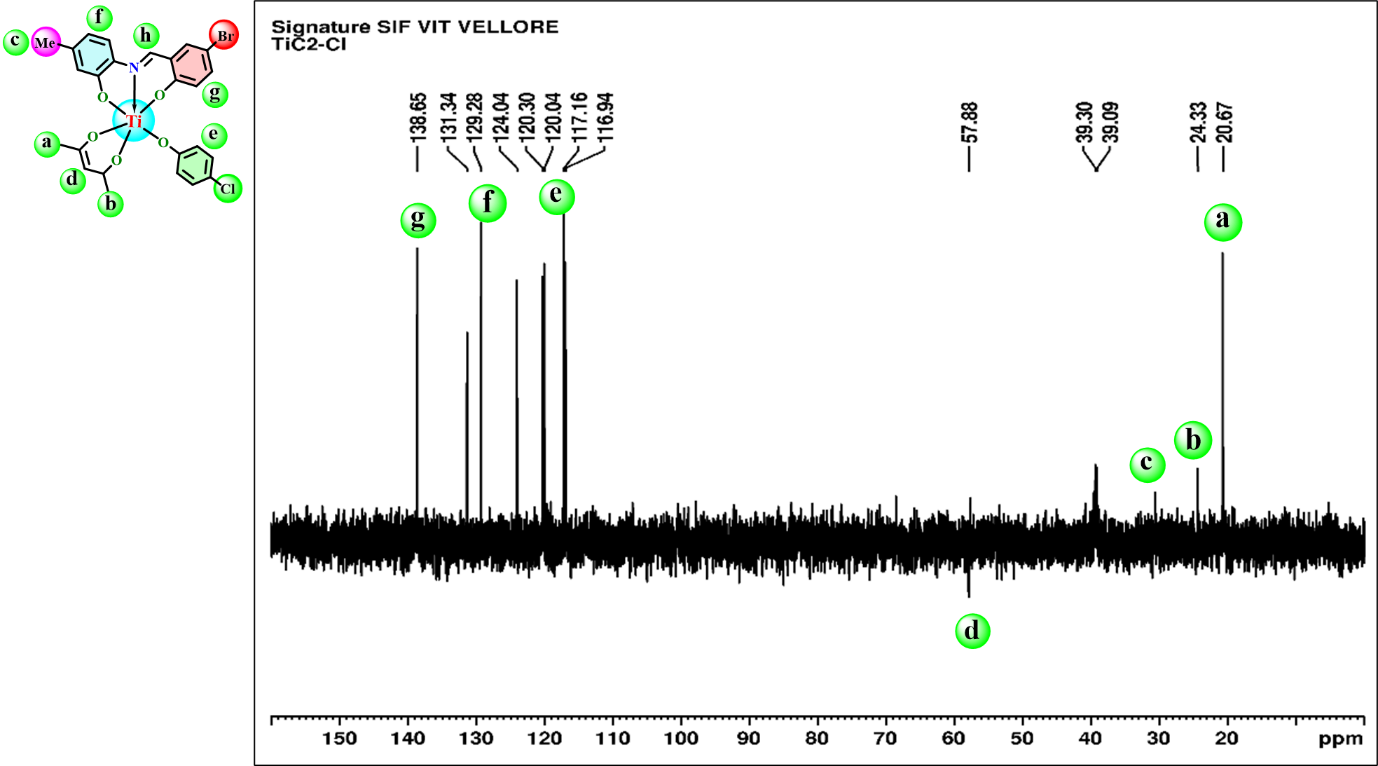


**Figure.S14. DEPT-135 NMR spectrum of TiC2****(400 MHz, DMSO-d_6_)**

|  |  |
| --- | --- |


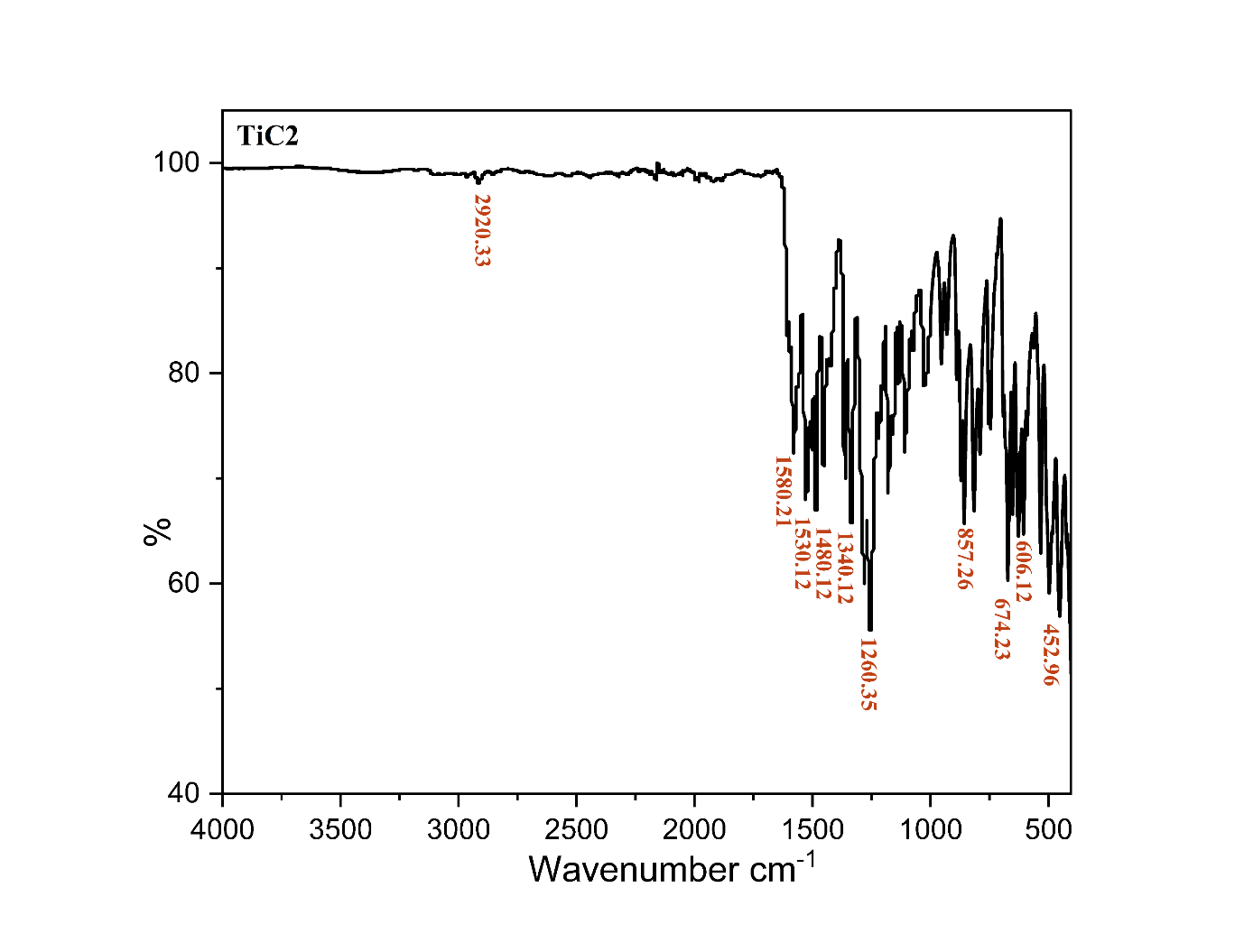


**Figure.S15. FT- IR spectrum of TiC2**


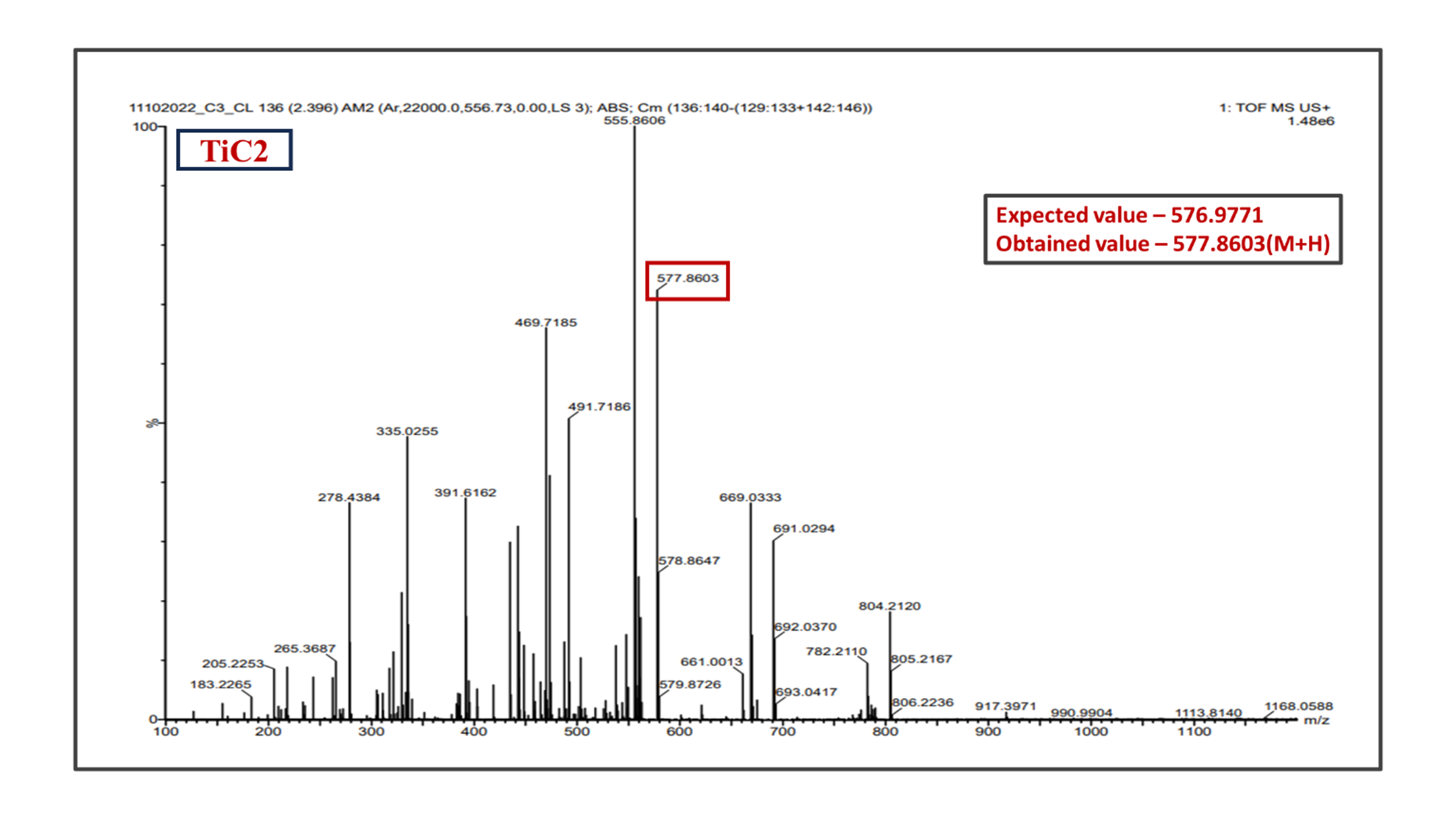


**Figure S16. Electrospray ionization ESI‐MS (m/z) spectrum of TiC2**


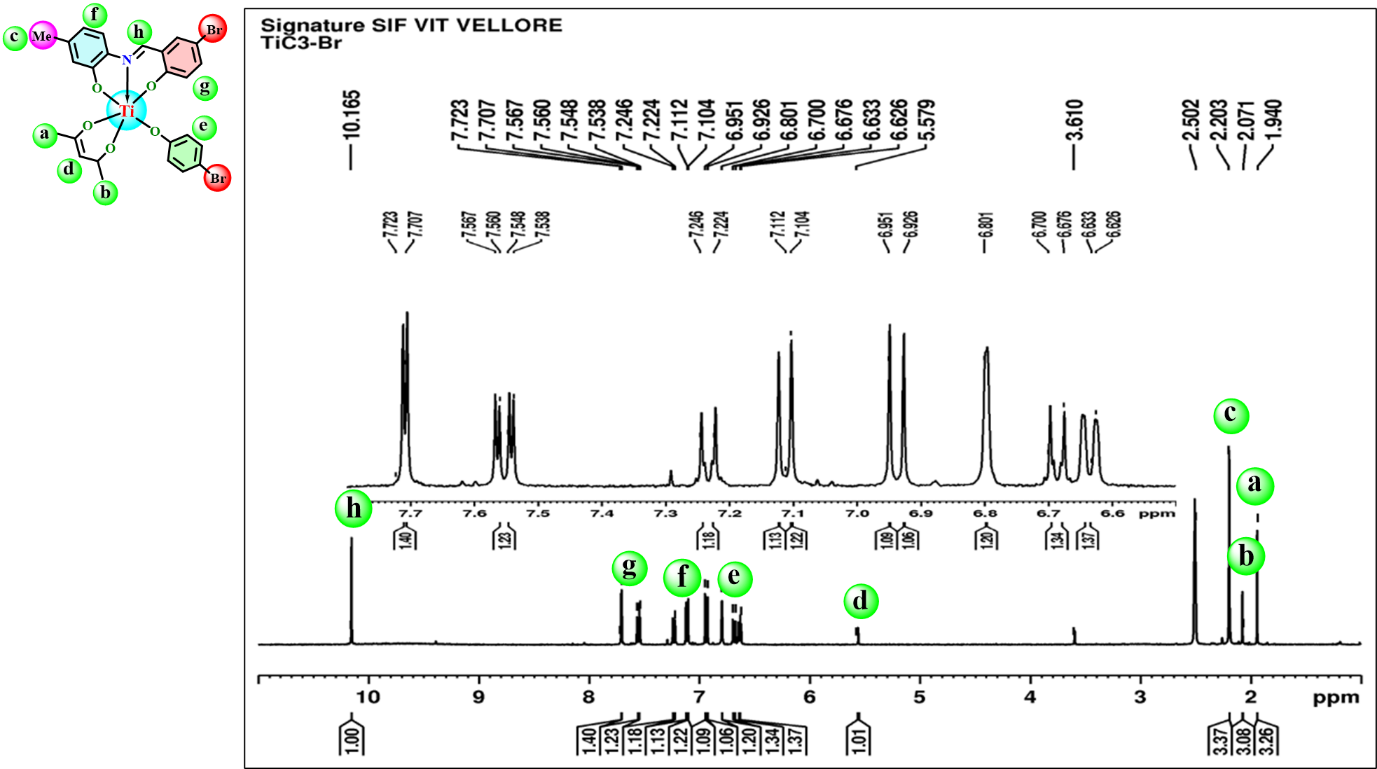


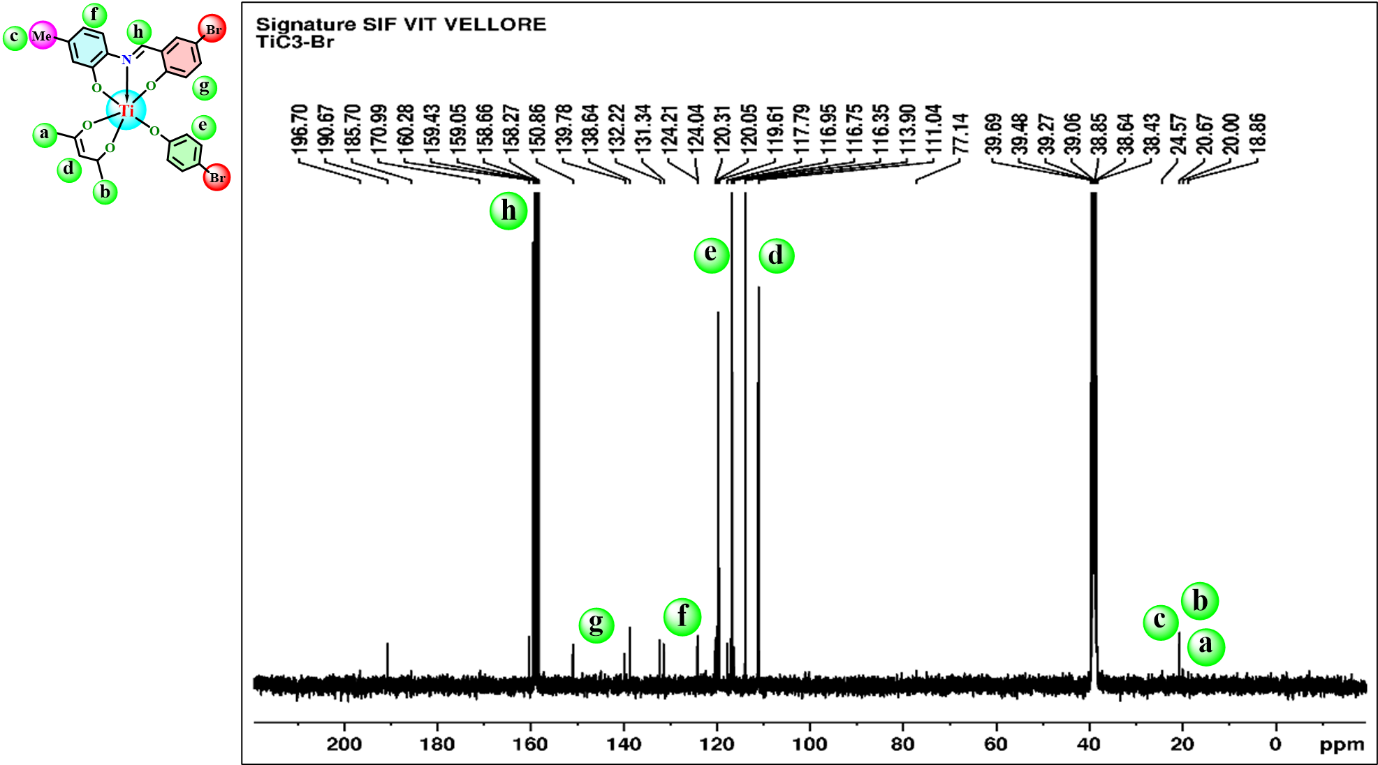
**Figure.S17.** **^1^H NMR spectrum of TiC3(400 MHz, DMSO-d_6_)**

**Figure.S18. ^13^ C NMR spectrum of TiC3(400 MHz, DMSO-d_6_)**


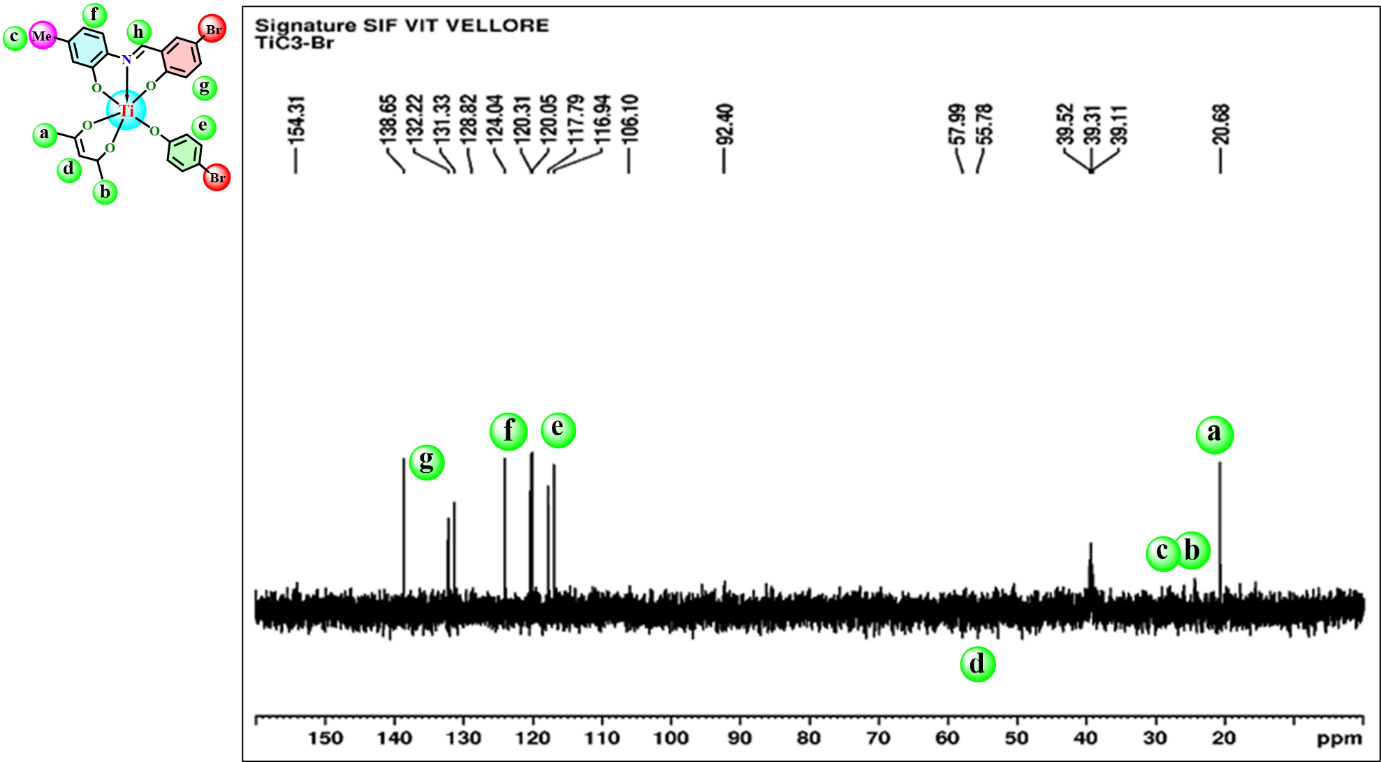


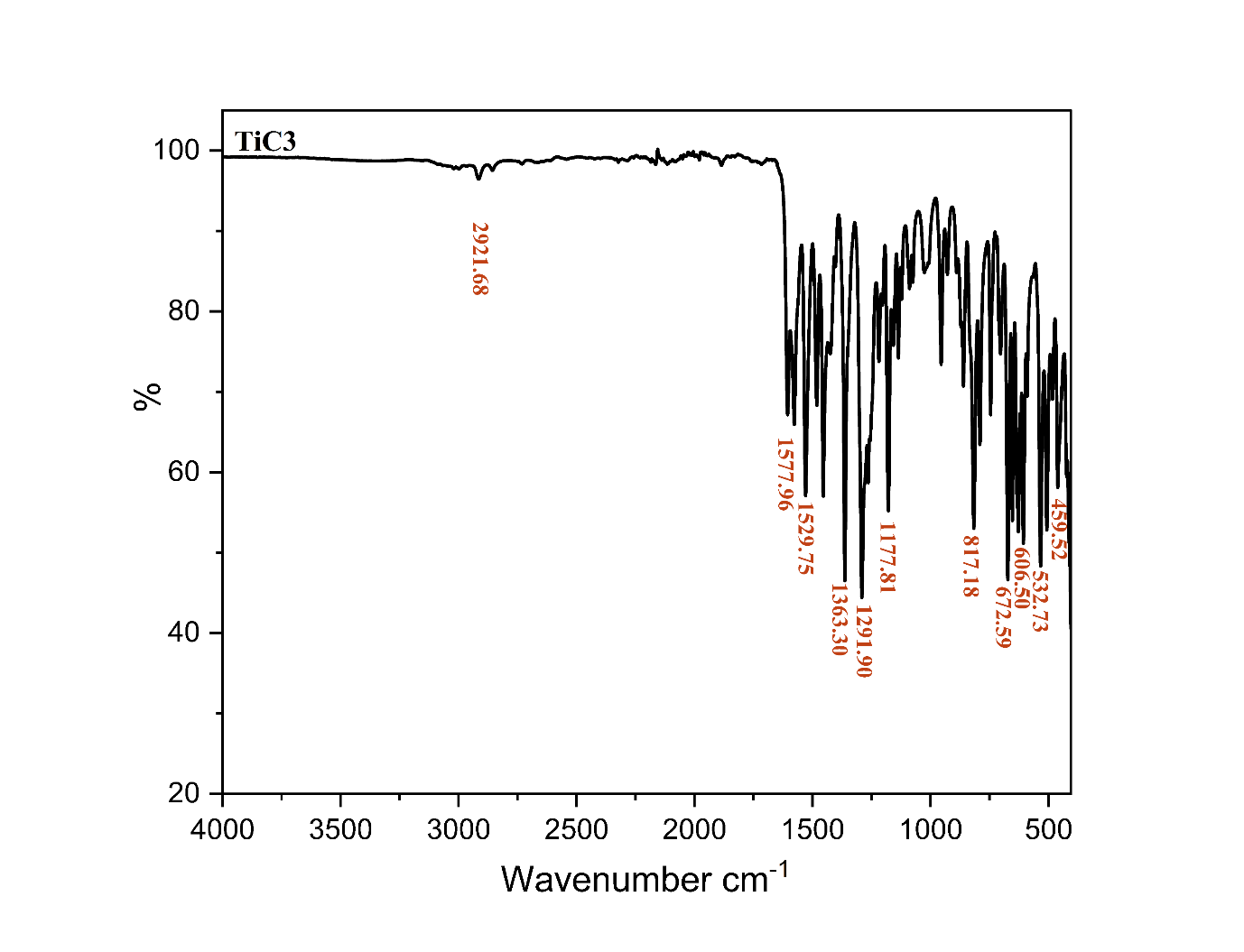
**Figure.S19. DEPT-135 NMR spectrum of TiC3(400 MHz, DMSO-d_6_)**

**Figure.S20. FT- IR spectrum of TiC3**


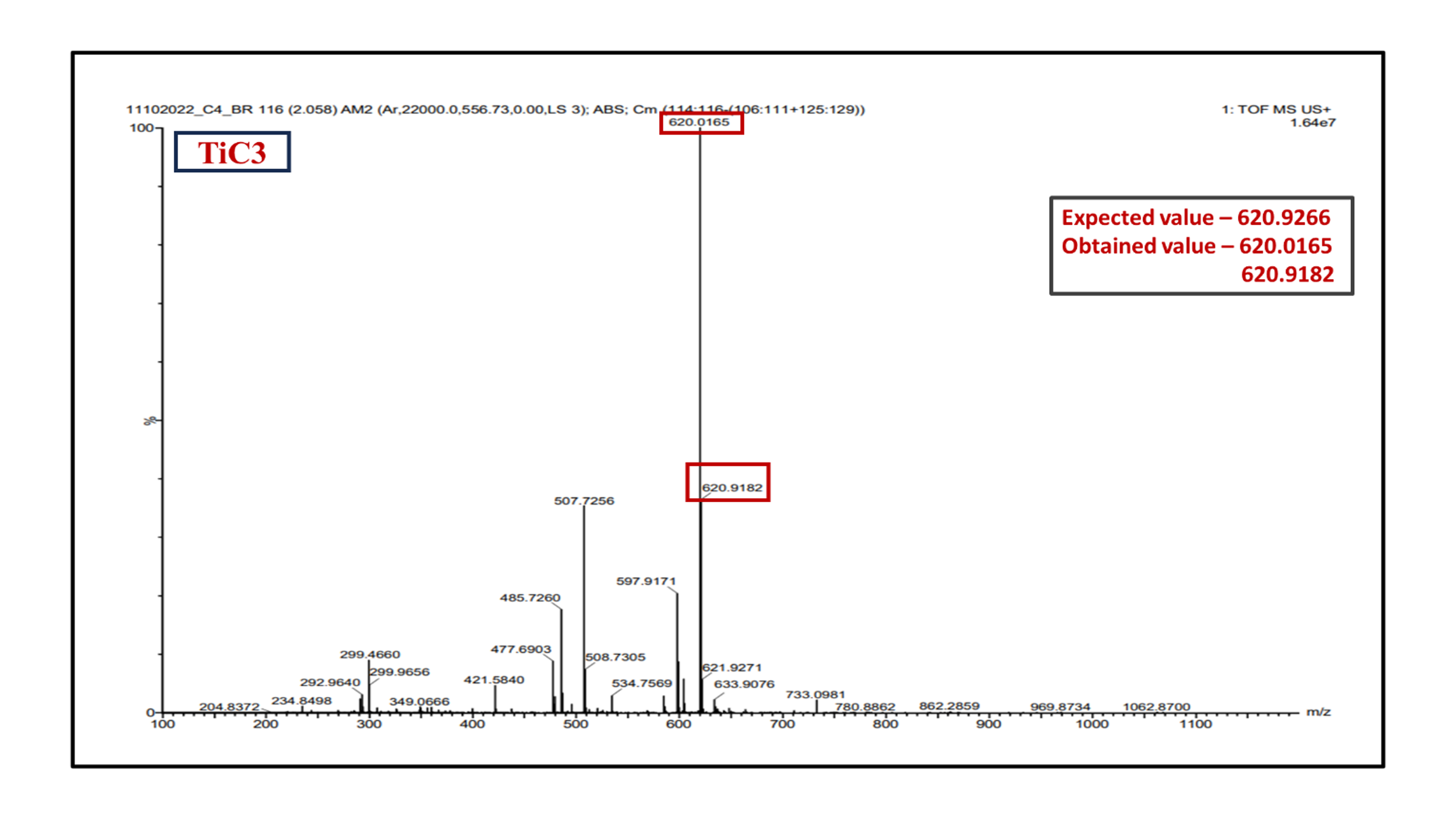


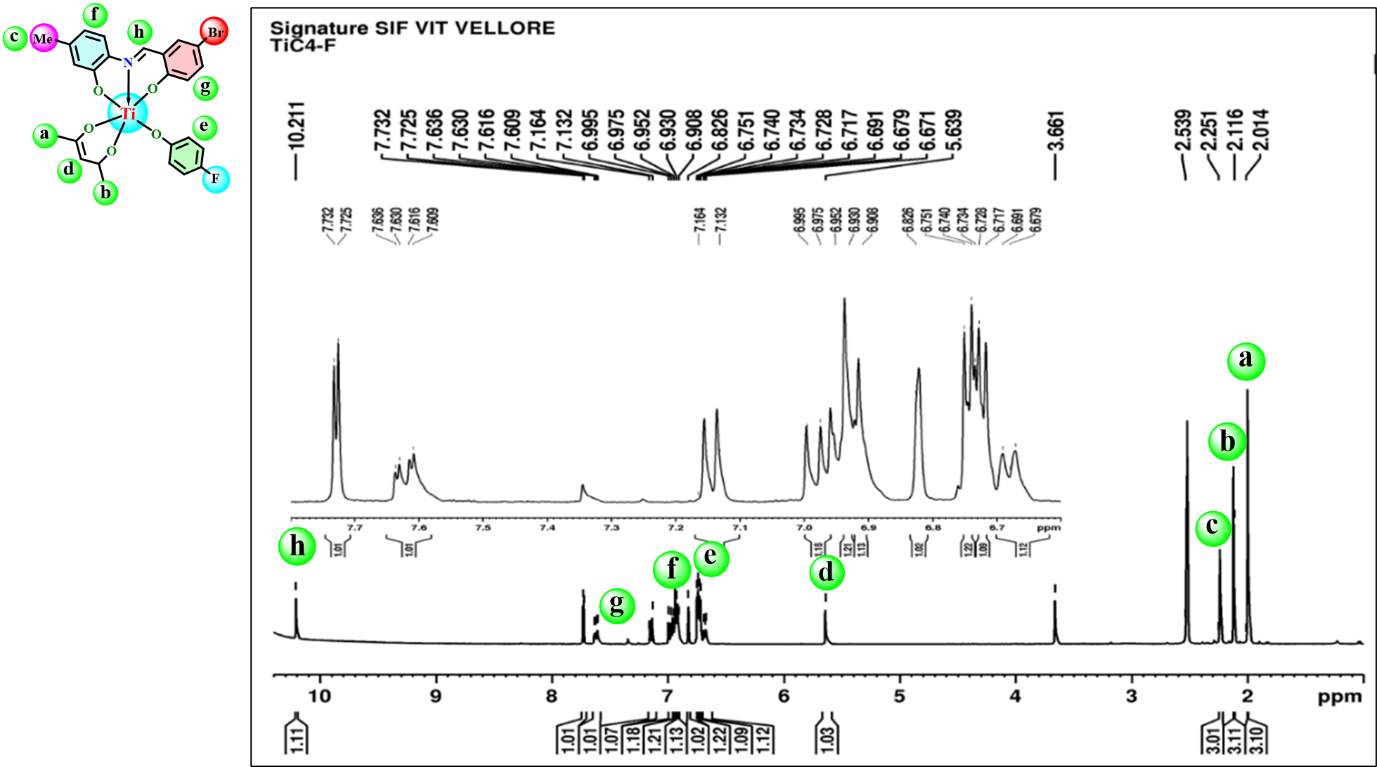
**Figure.S21. Electrospray ionization ESI‐MS (m/z) spectrum of TiC3**

**Figure.S22. ^1^H NMR spectrum of TiC4(400 MHz, DMSO-d_6_)**


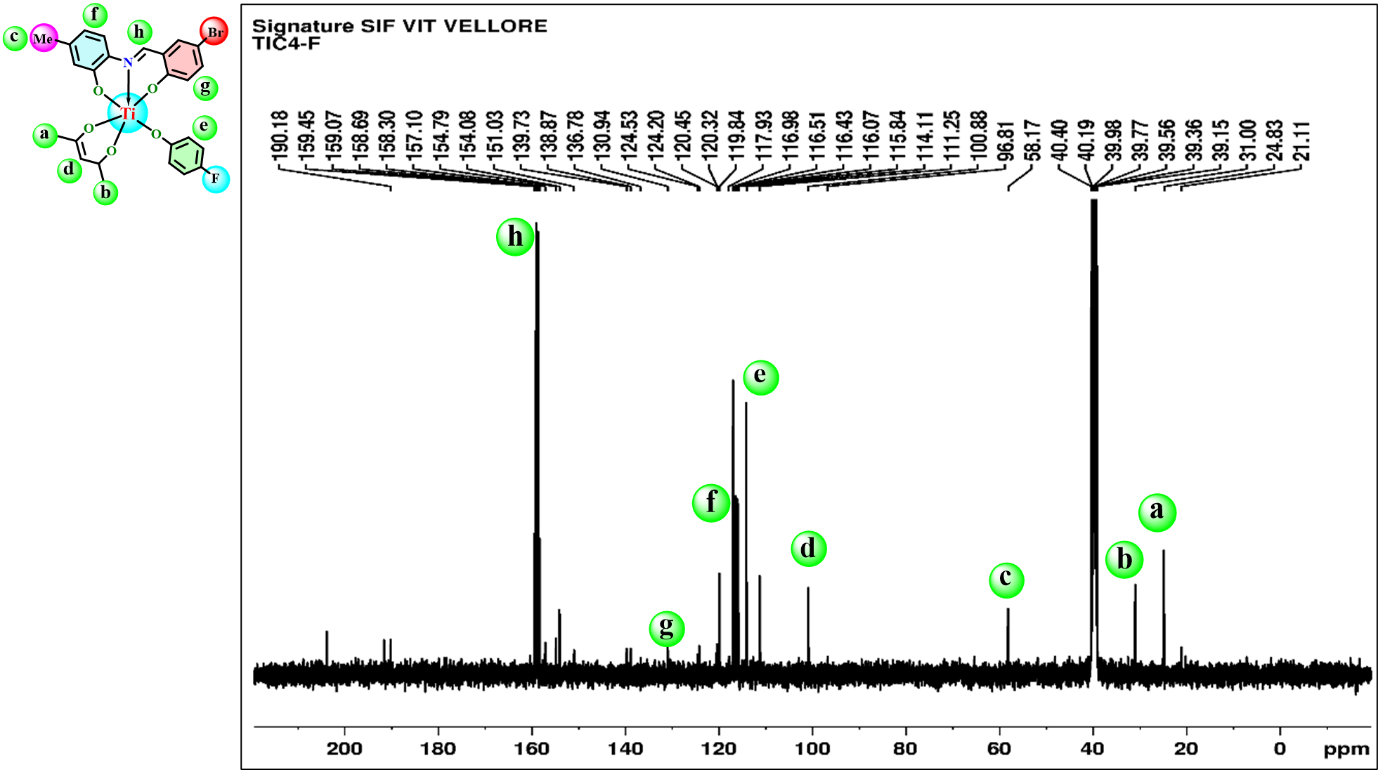


**Figure.S23. ^13^C NMR spectrum of TiC4(400 MHz, DMSO-d_6_)**


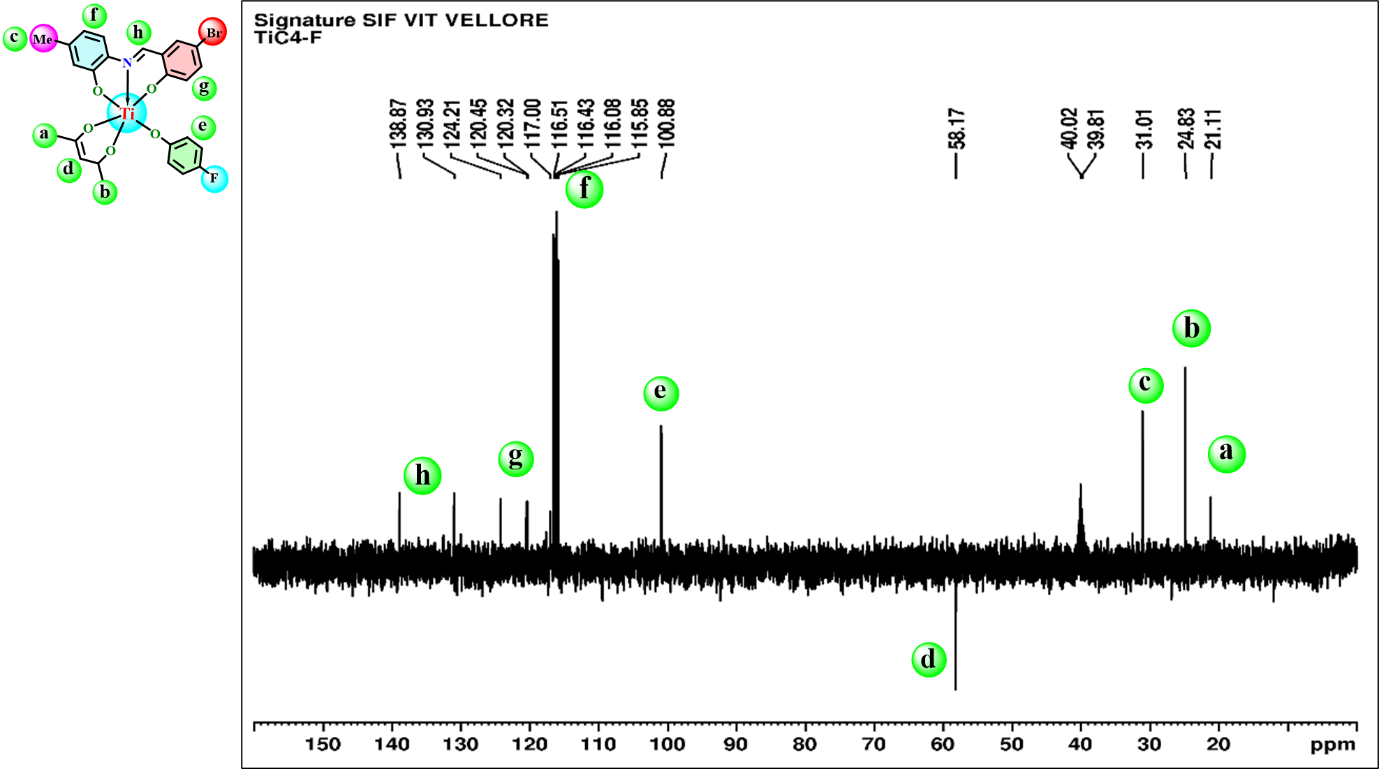


**Figure.S24. DEPT-135 NMR spectrum of TiC4(400 MHz, DMSO-d_6_)**


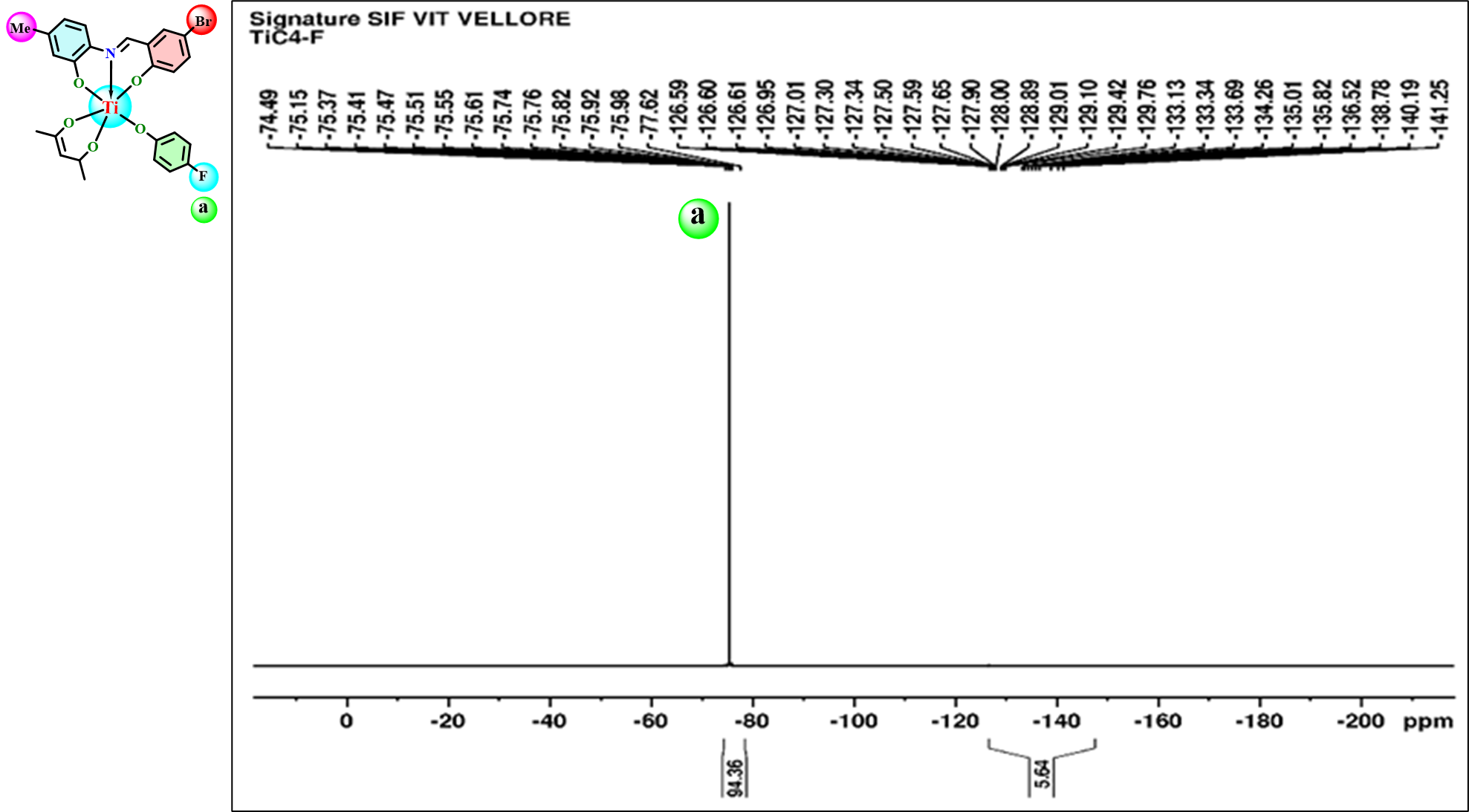


**Figure.S25. ^19^F NMR spectrum of TiC4(400 MHz, DMSO-d_6_)**


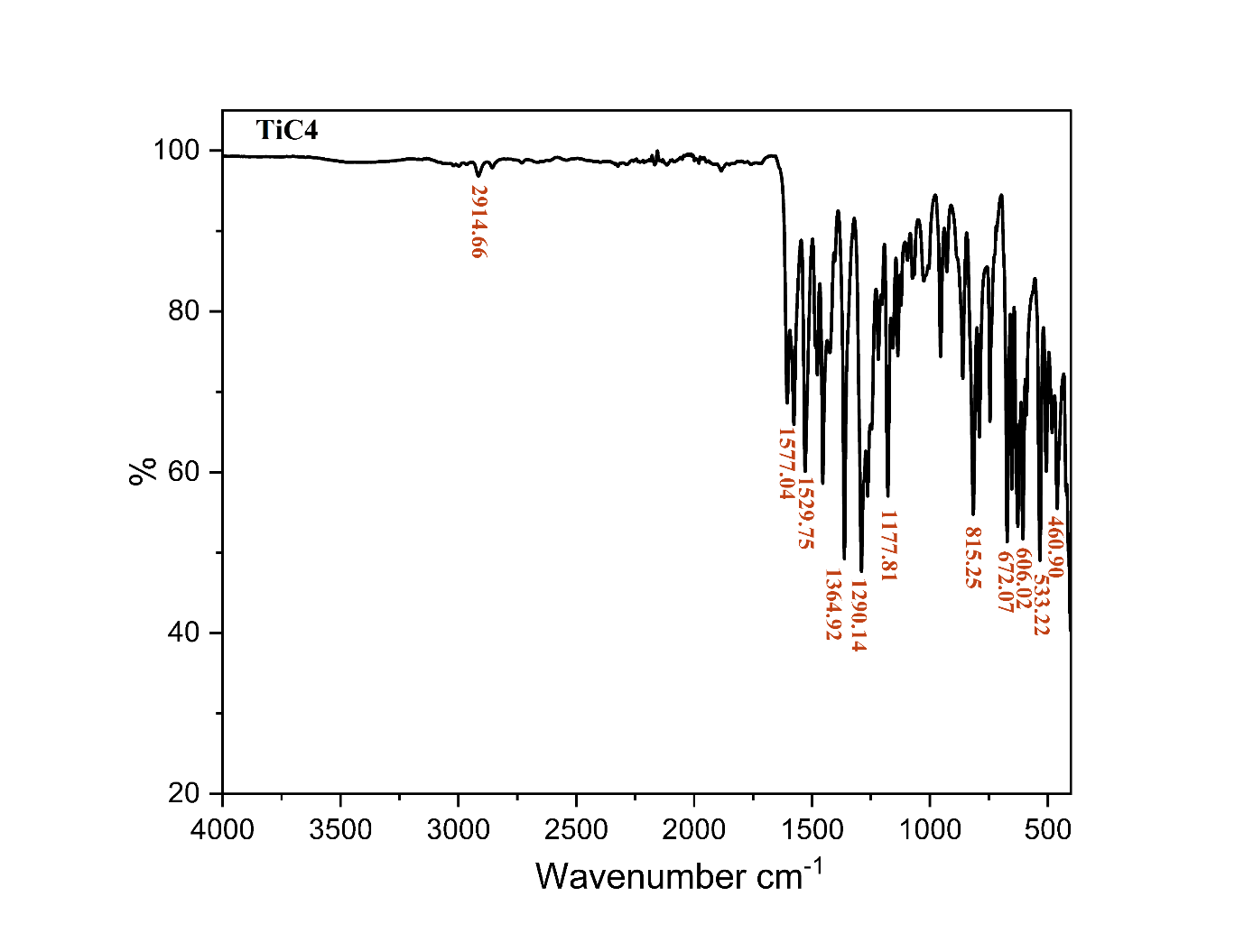
**Figure S26. FT- IR spectrum of TiC4**


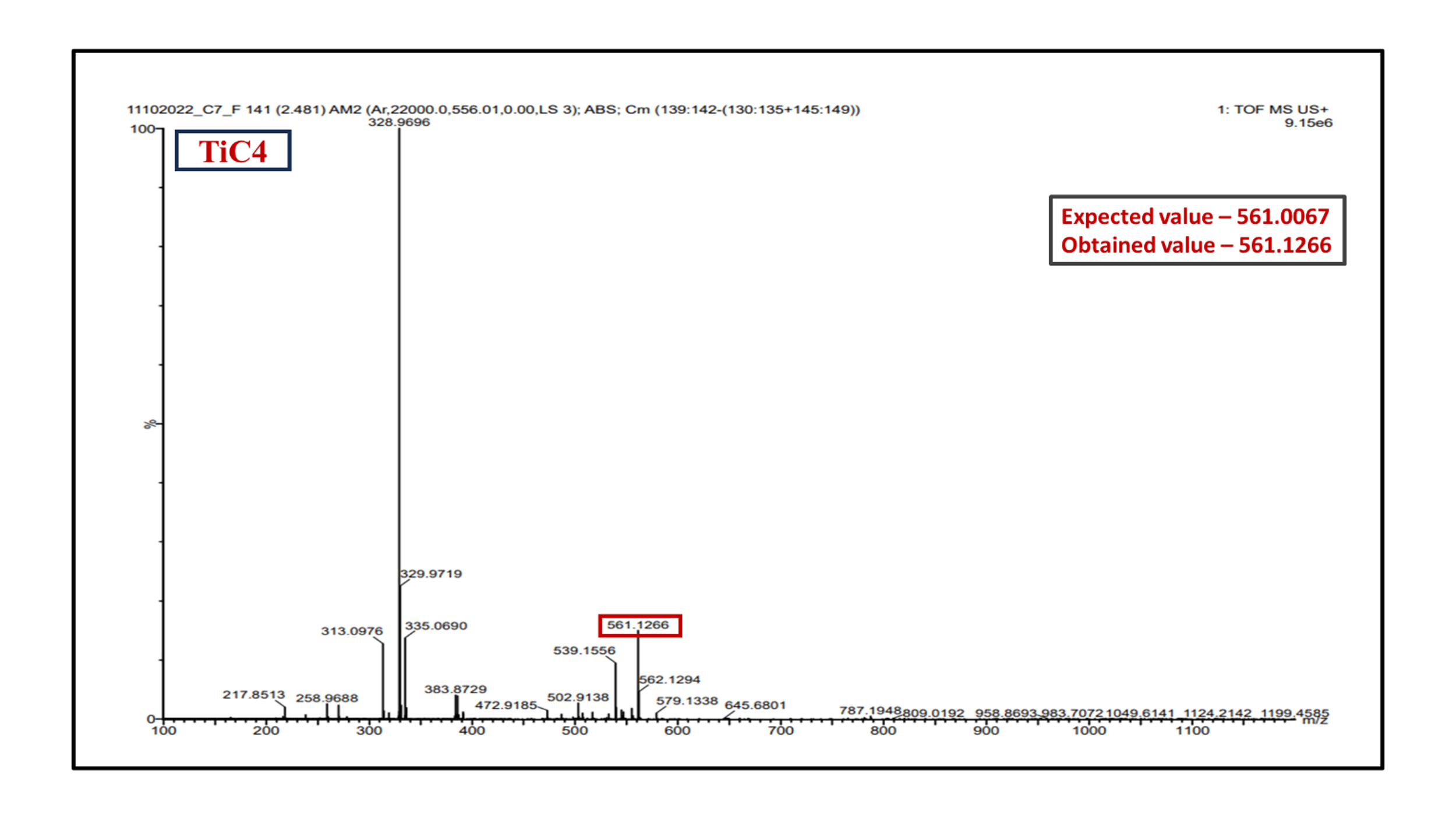


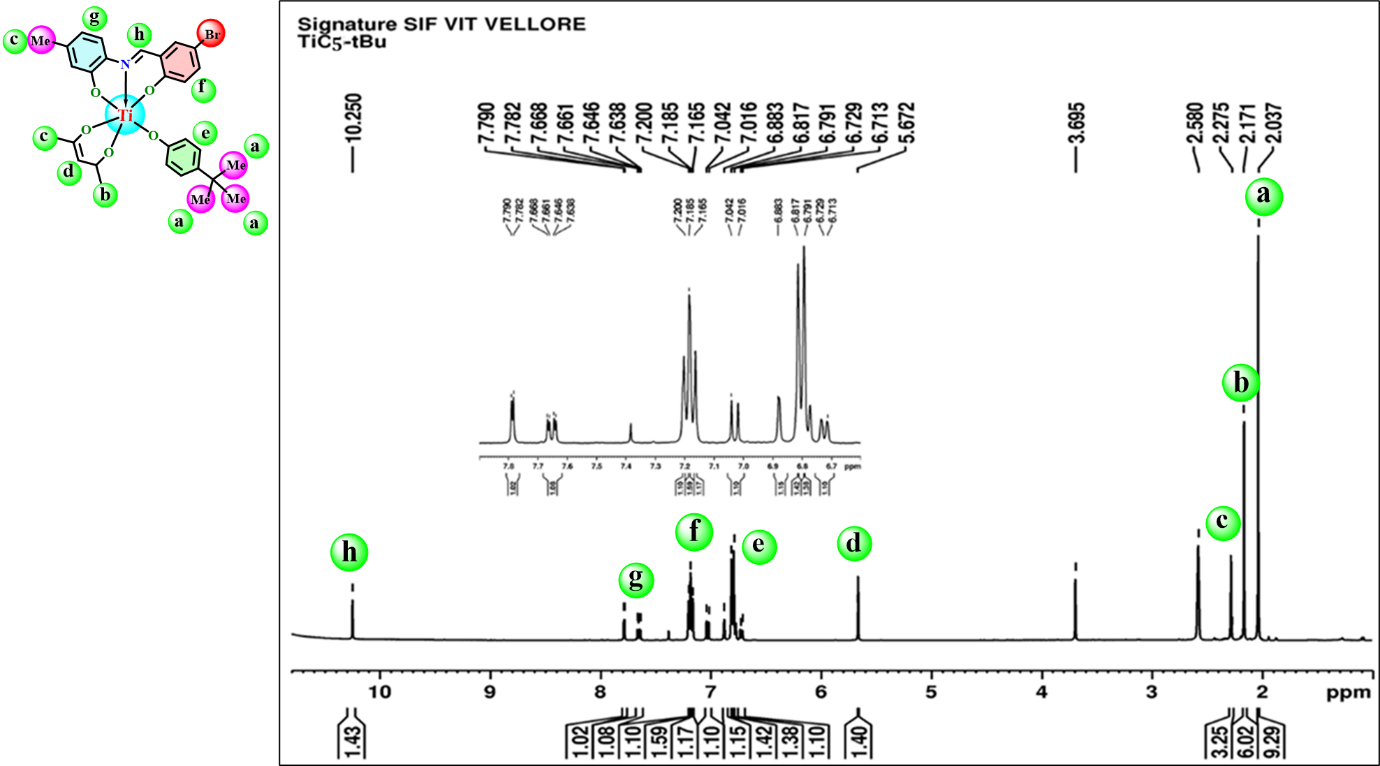
**Figure S27. Electrospray ionization ESI‐MS (m/z) spectrum of TiC4**

**Figure.S28. ^1^H NMR spectrum of TiC5(400 MHz, DMSO-d_6_)**


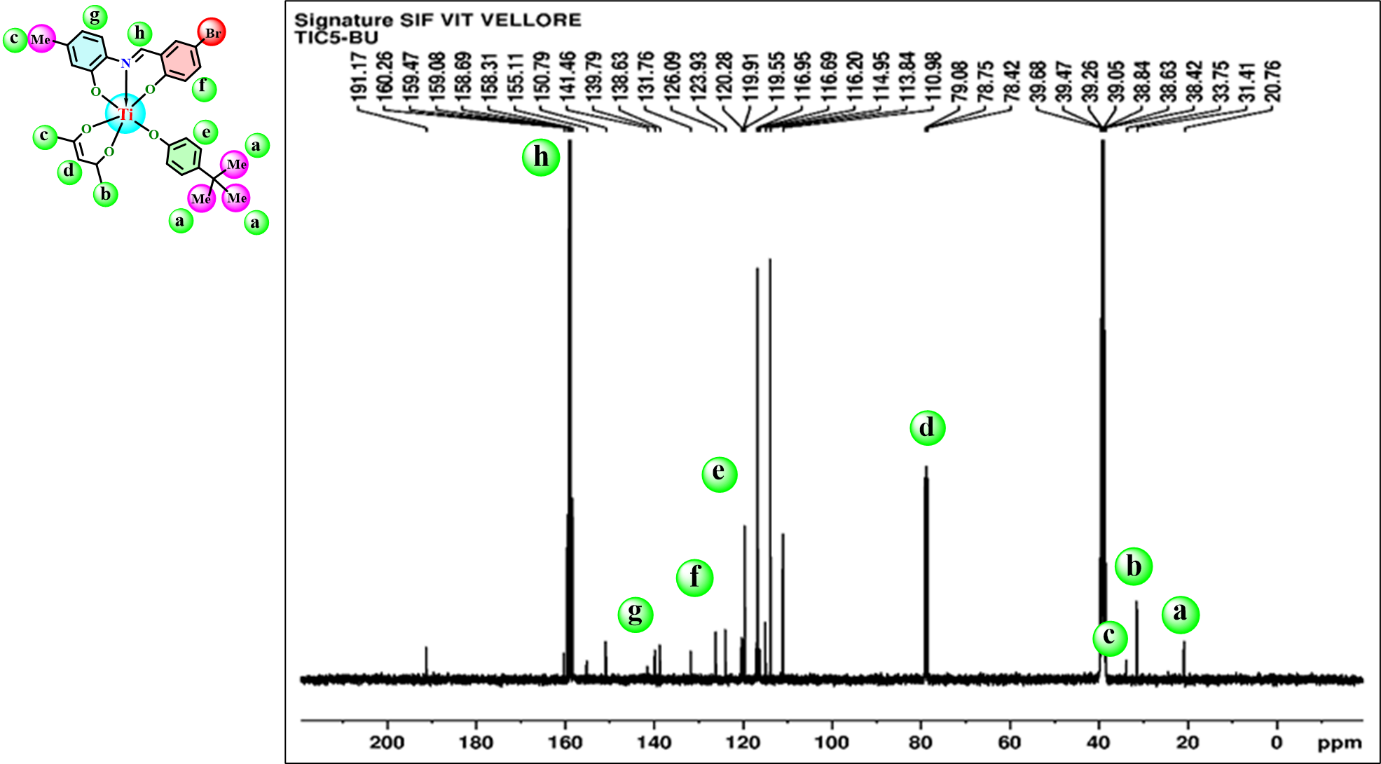


**Figure.S29. ^13^C NMR spectrum of TiC5(400 MHz, DMSO-d_6_)**


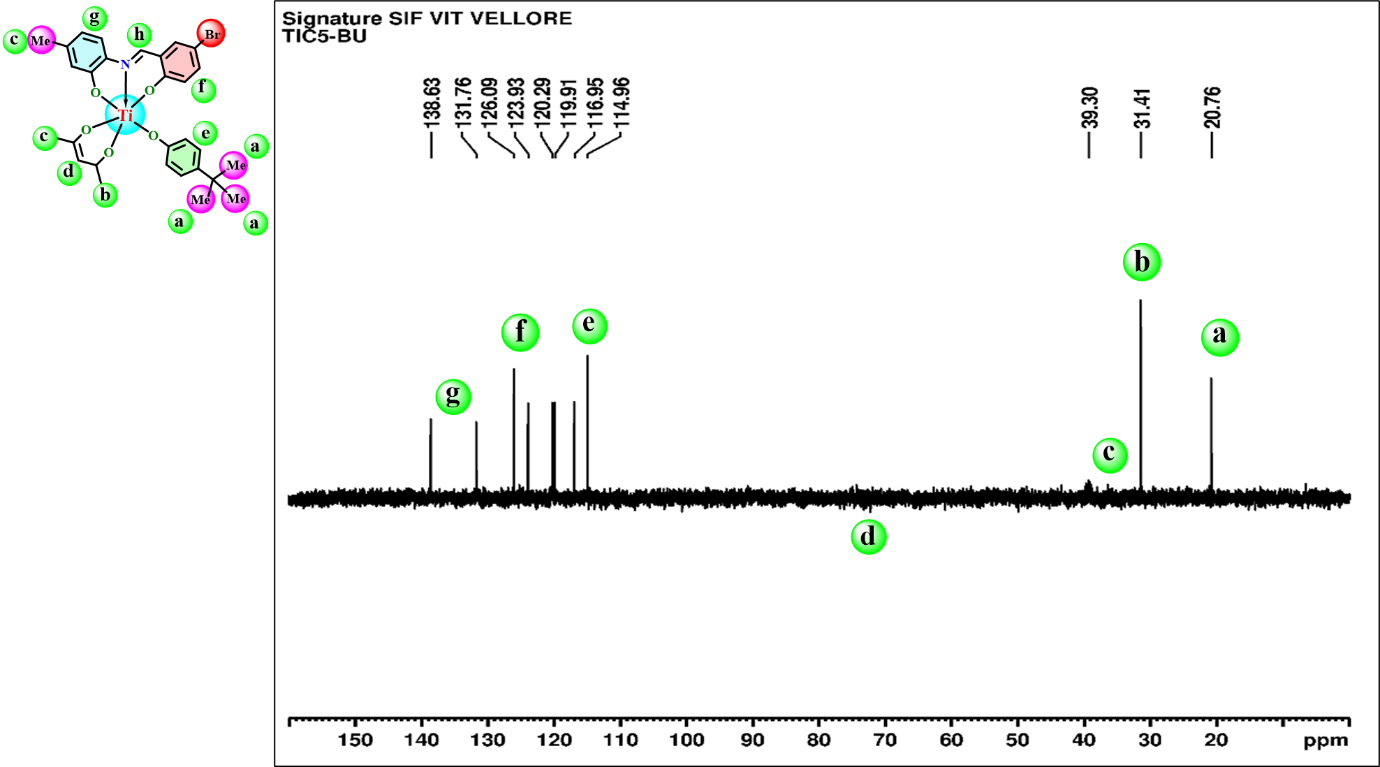


**Figure.S30. DEPT-135 NMR spectrum of TiC5(400 MHz, DMSO-d_6_)**


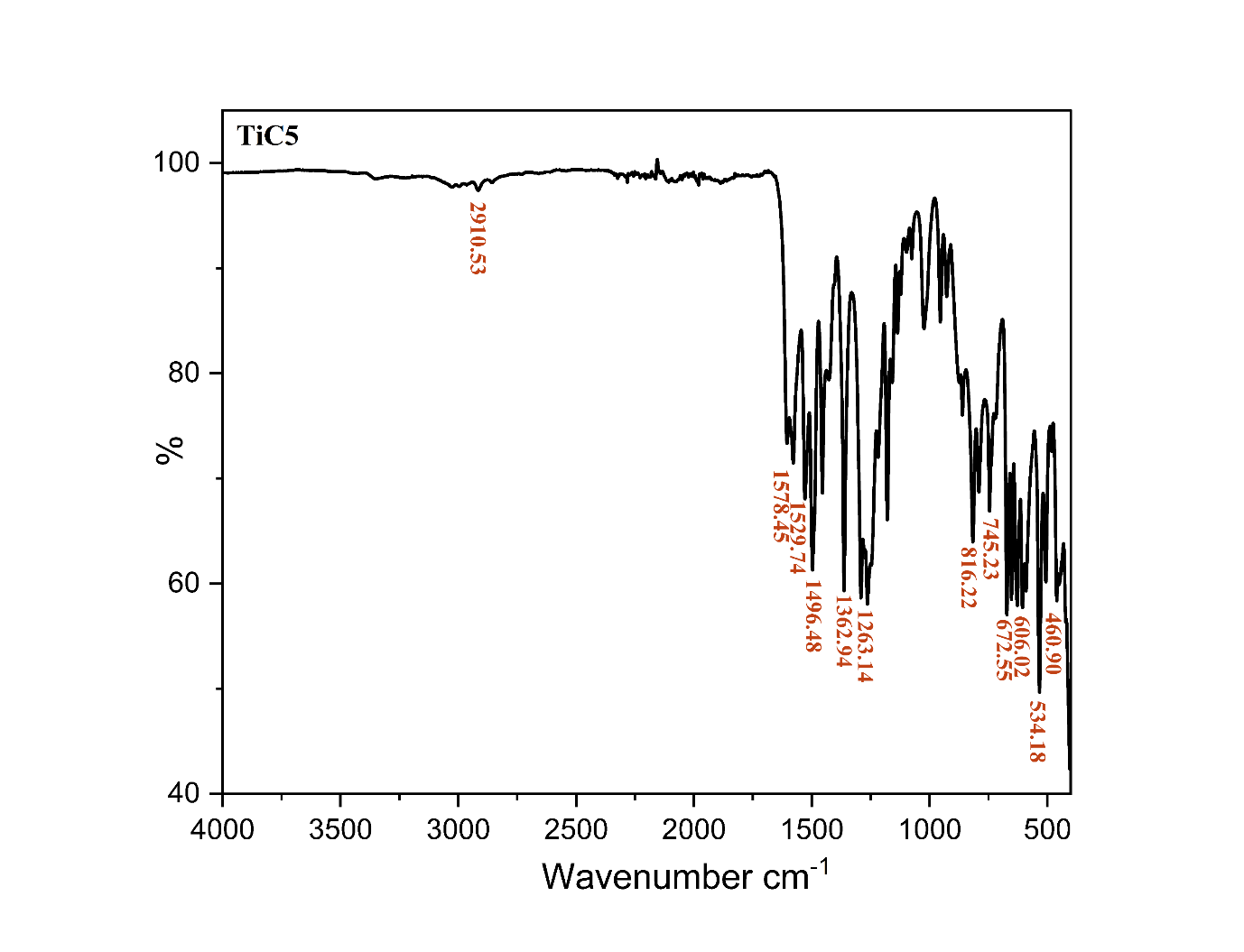


**Figure.S31. FT- IR spectrum of TiC5**


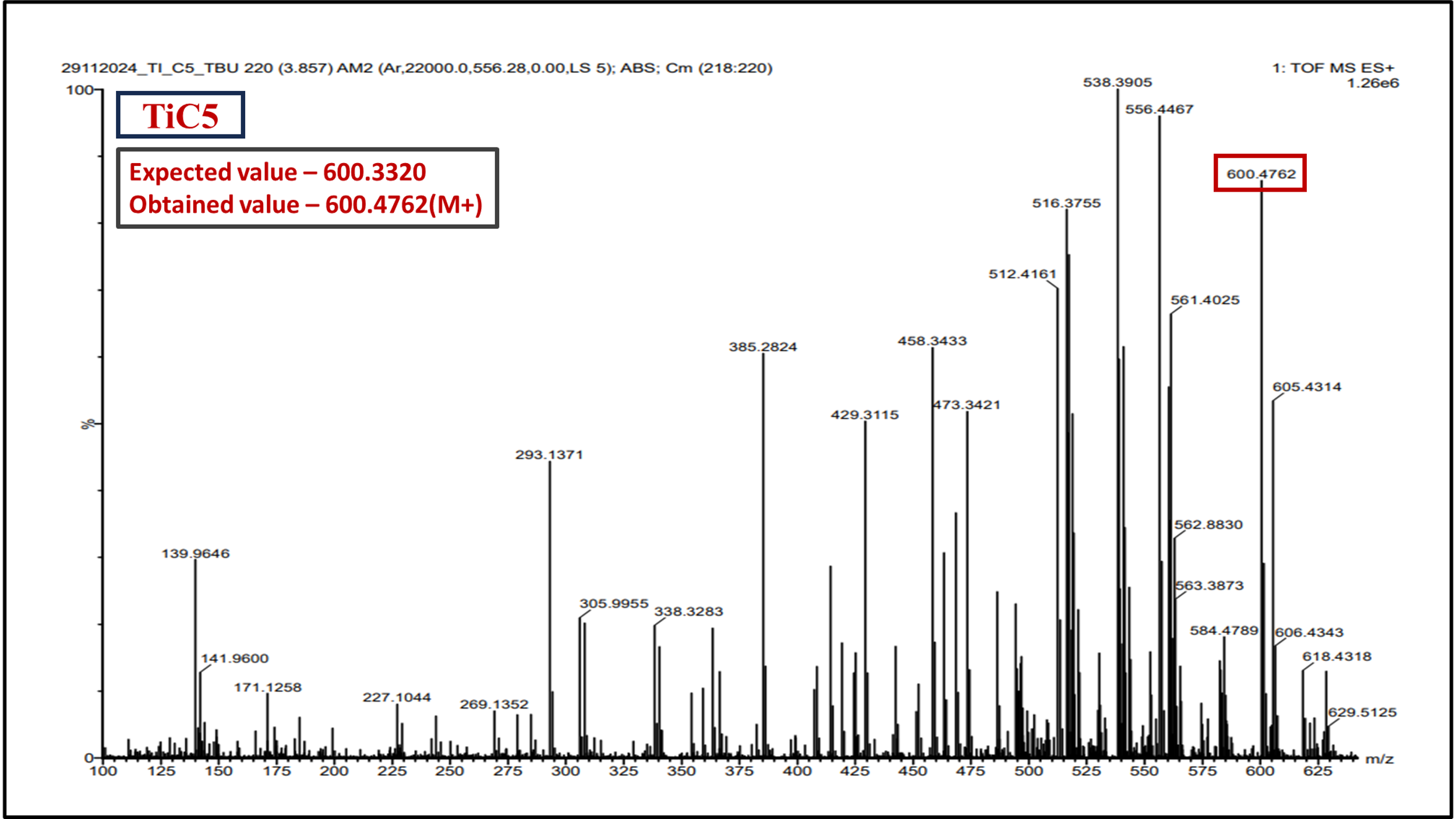


**Figure.S32. Electrospray ionization ESI‐MS (m/z) spectrum of TiC5**


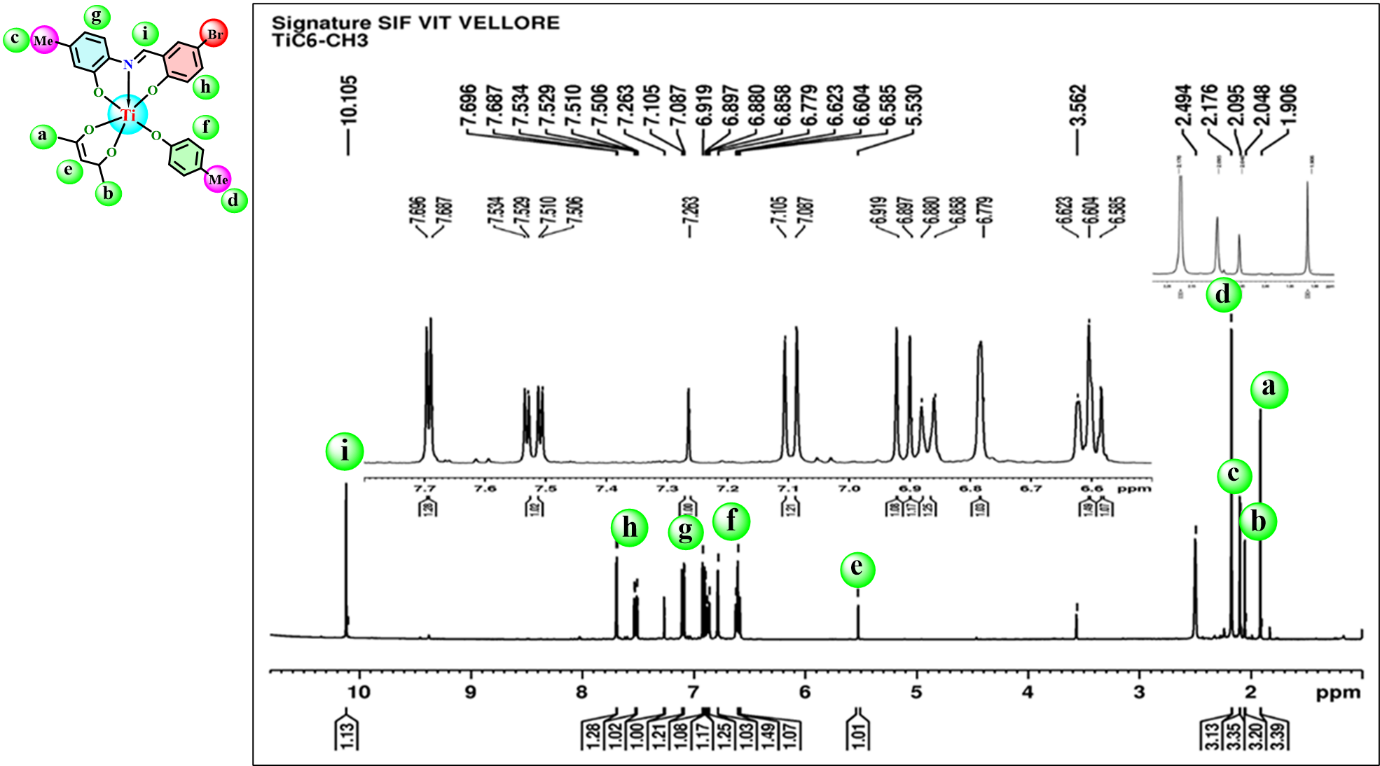


**Figure.S33. ^1^H NMR spectrum of TiC6 (400 MHz, DMSO-d_6_)**


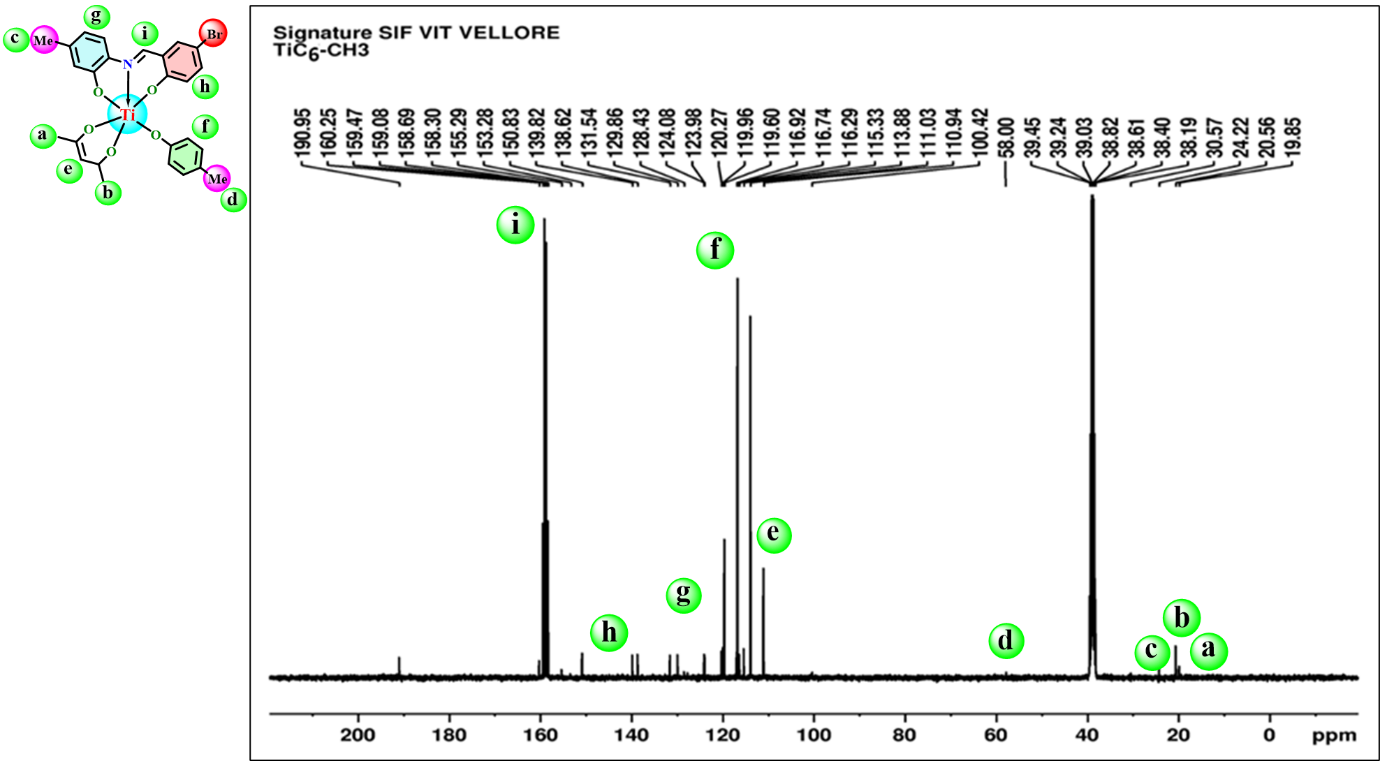


**Figure.S34. ^13^C NMR spectrum of TiC6(400 MHz, DMSO-d_6_)**


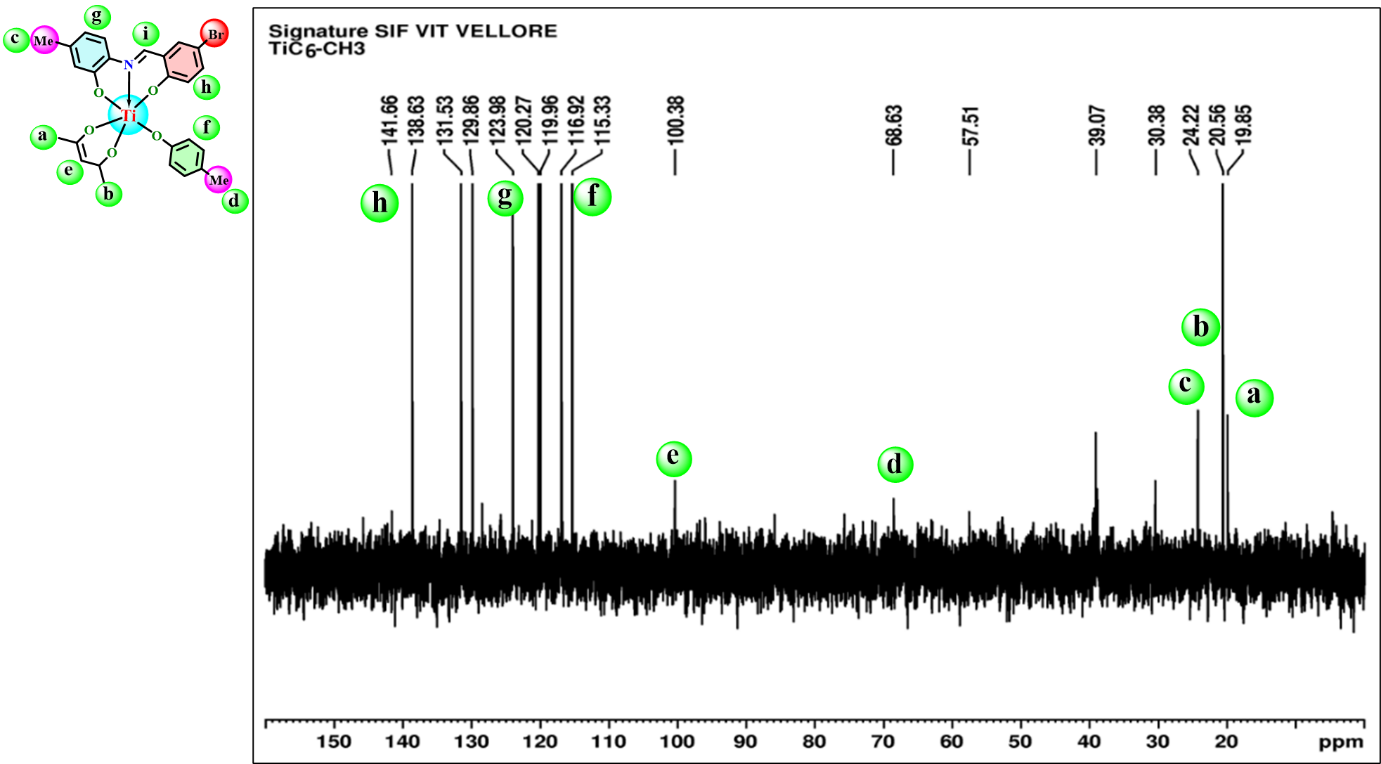


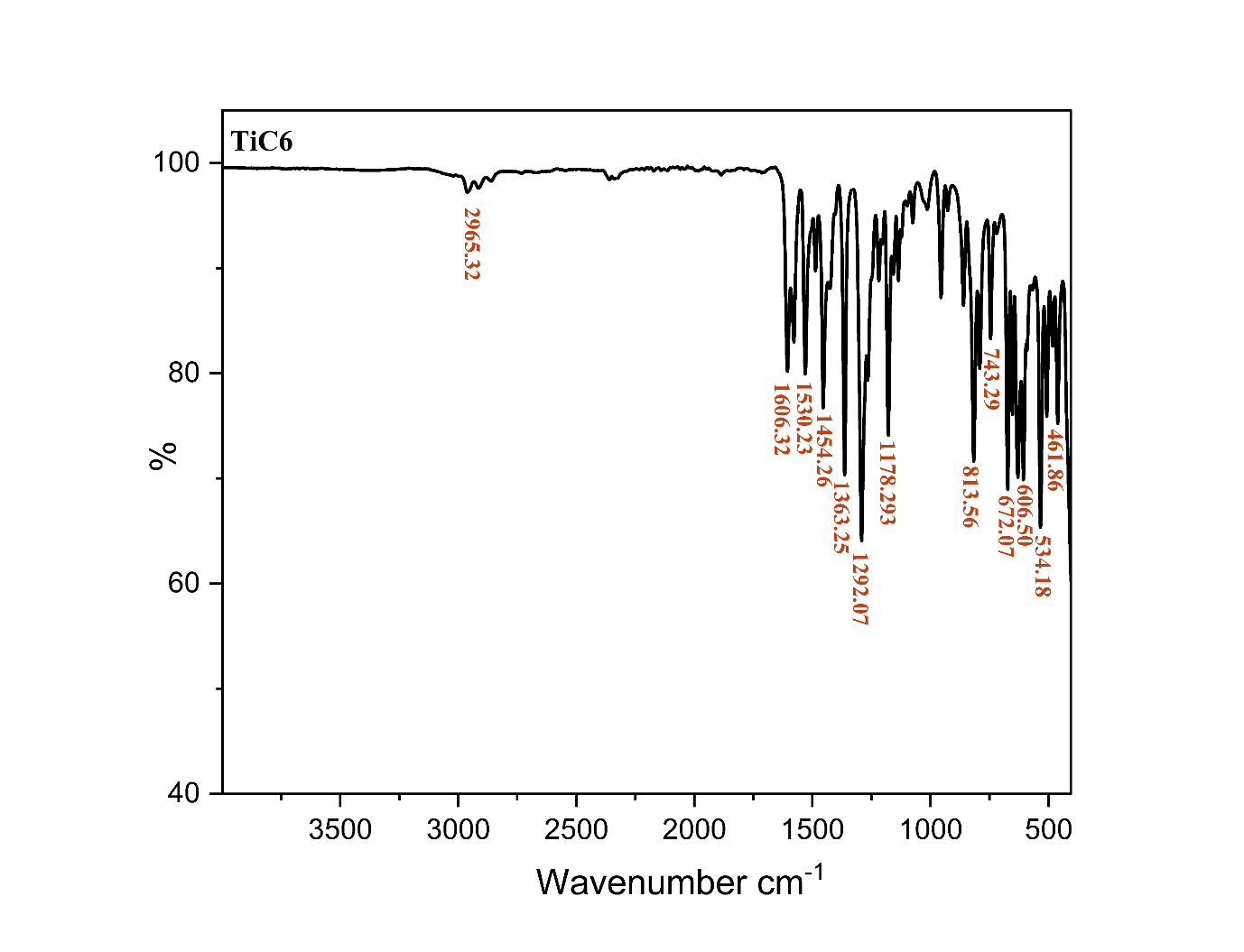
**Figure.S35. DEPT-135 NMR spectrum of TiC6(400 MHz, DMSO-d_6_)**

**Figure.S36. FT- IR spectrum of TiC6**


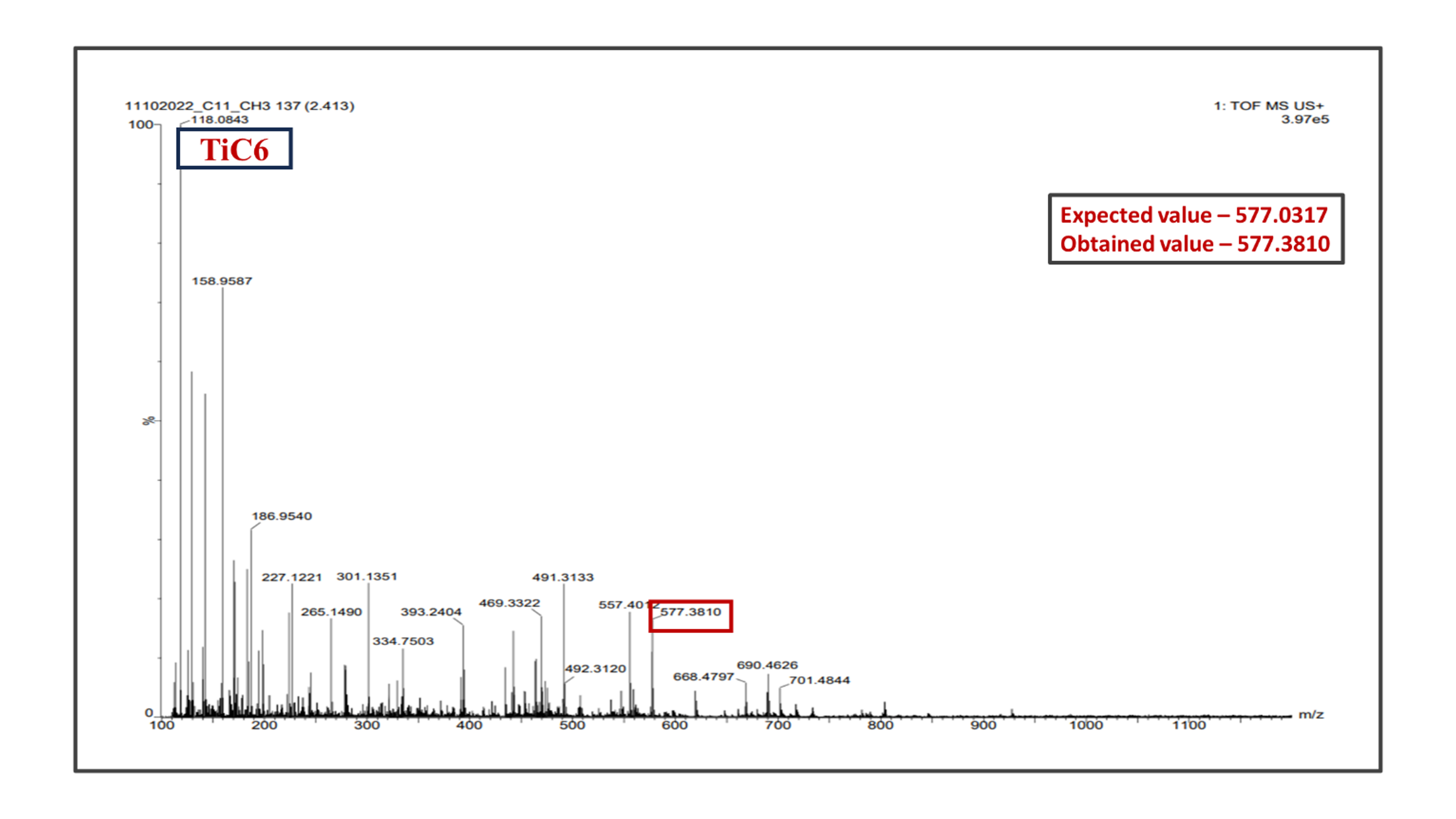


**Figure.S37. Electrospray ionization ESI‐MS (m/z) spectrum of TiC6**


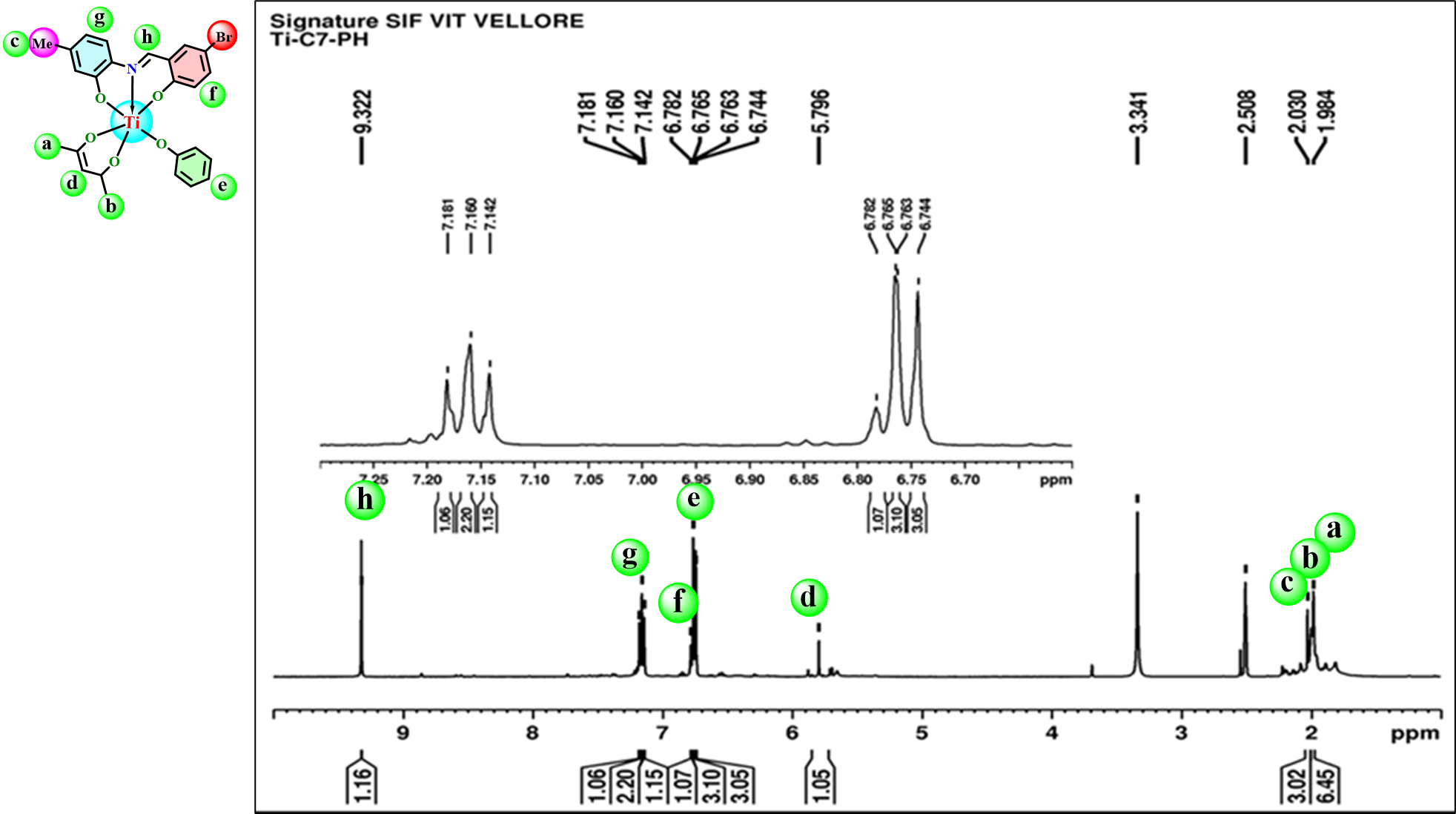


**Figure.S38. ^1^H NMR spectrum of TiC7(400 MHz, DMSO-d_6_)**


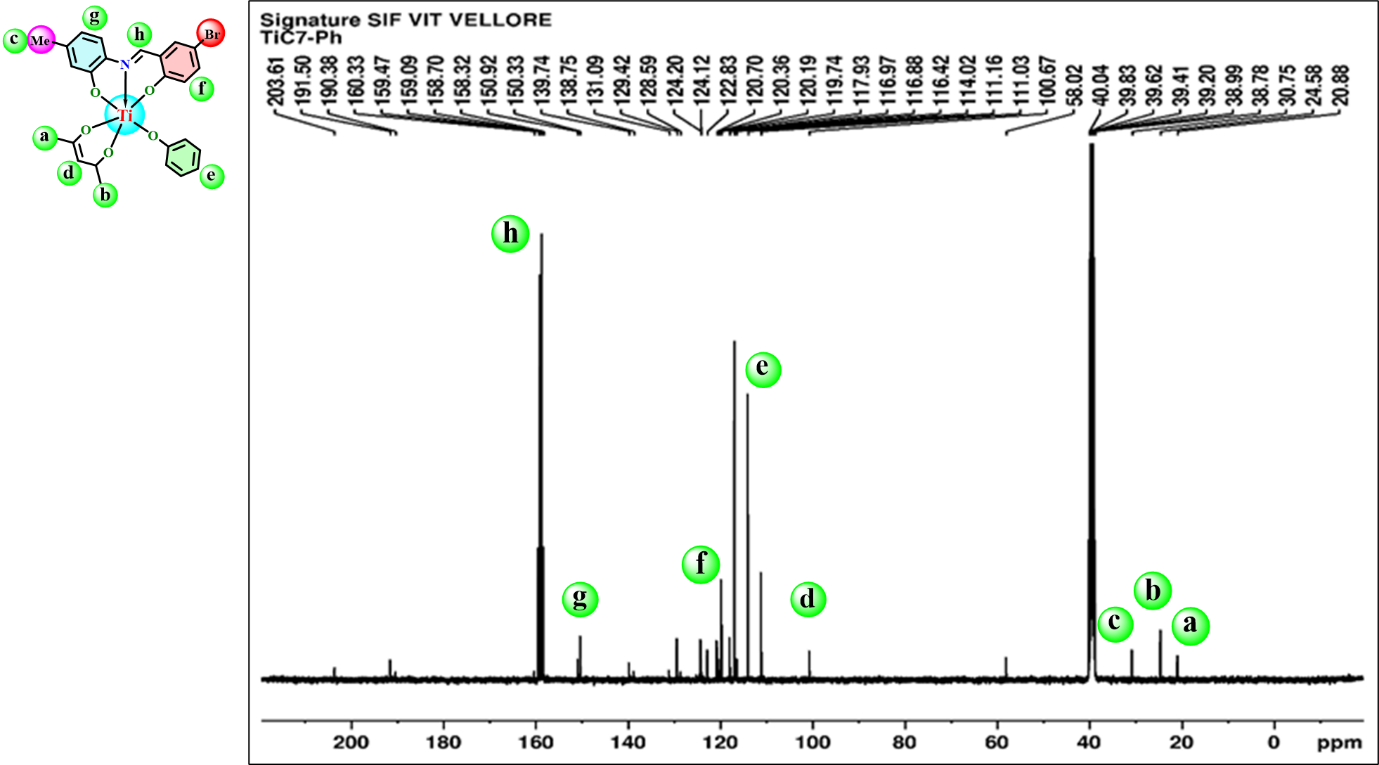


**Figure.S39. ^13^C NMR spectrum of TiC7(400 MHz, DMSO-d_6_)**


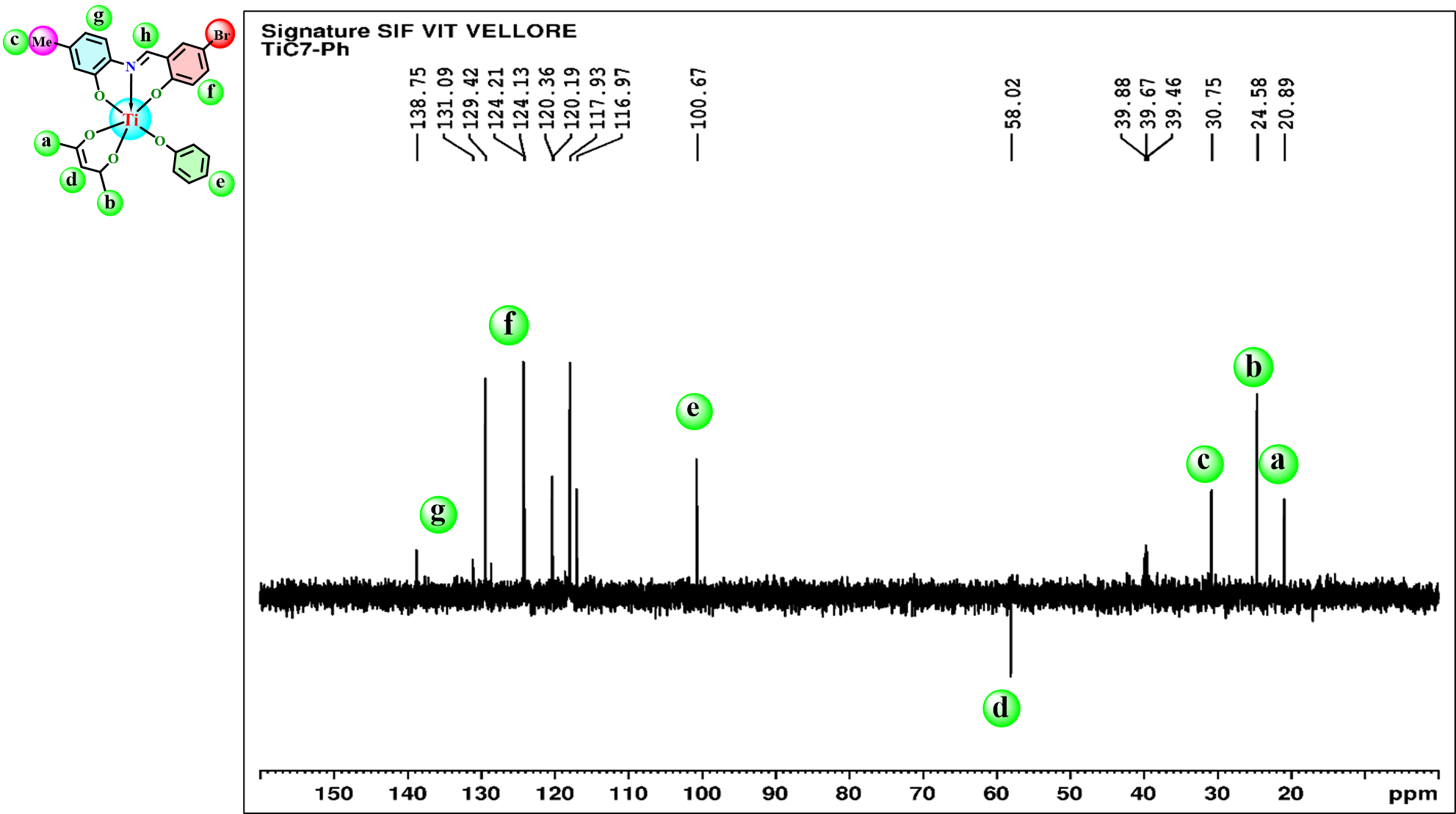


**Figure.S40. DEPT-135 NMR spectrum of TiC7(400 MHz, DMSO-d_6_)**


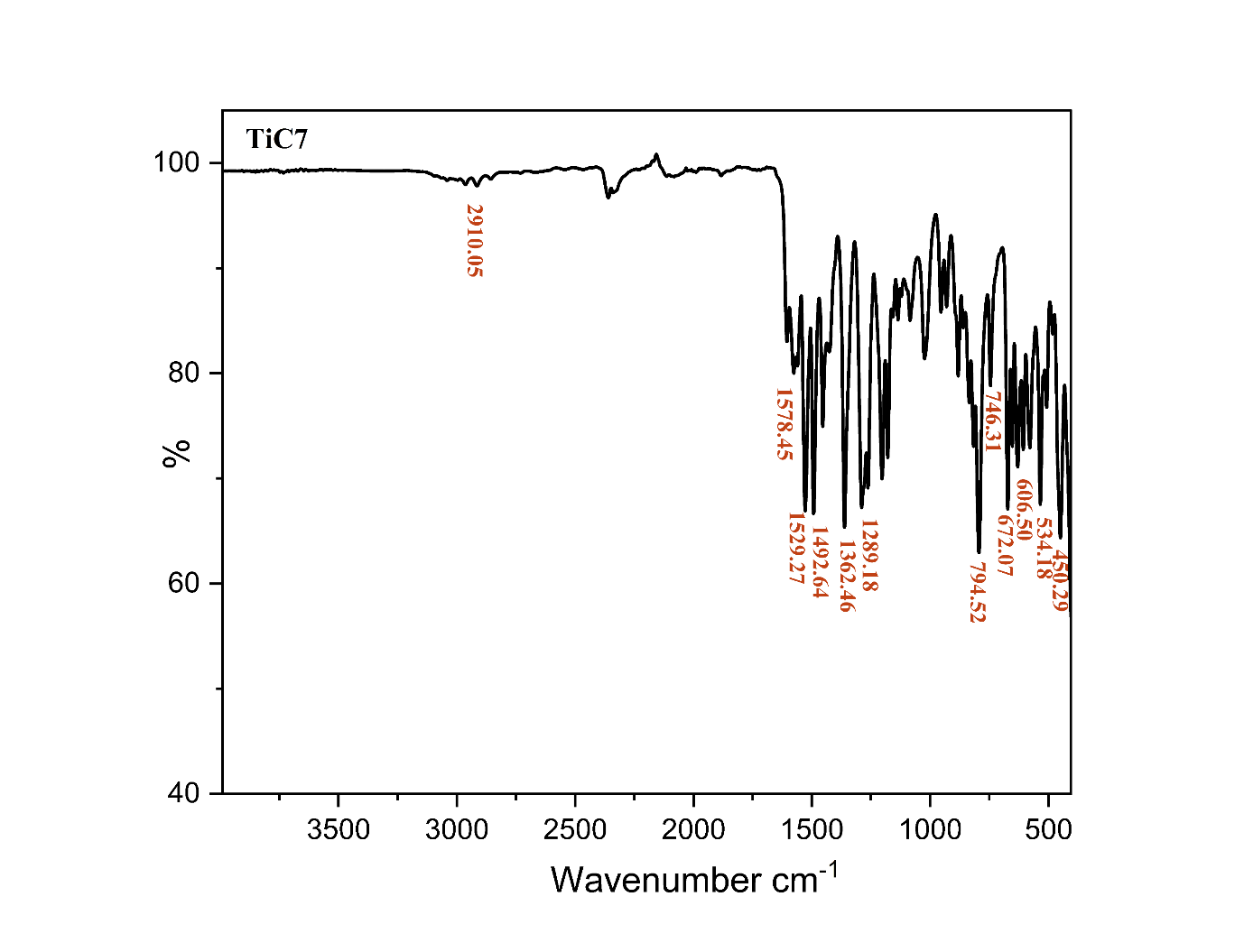


**Figure.S41. FT- IR spectrum of TiC7**


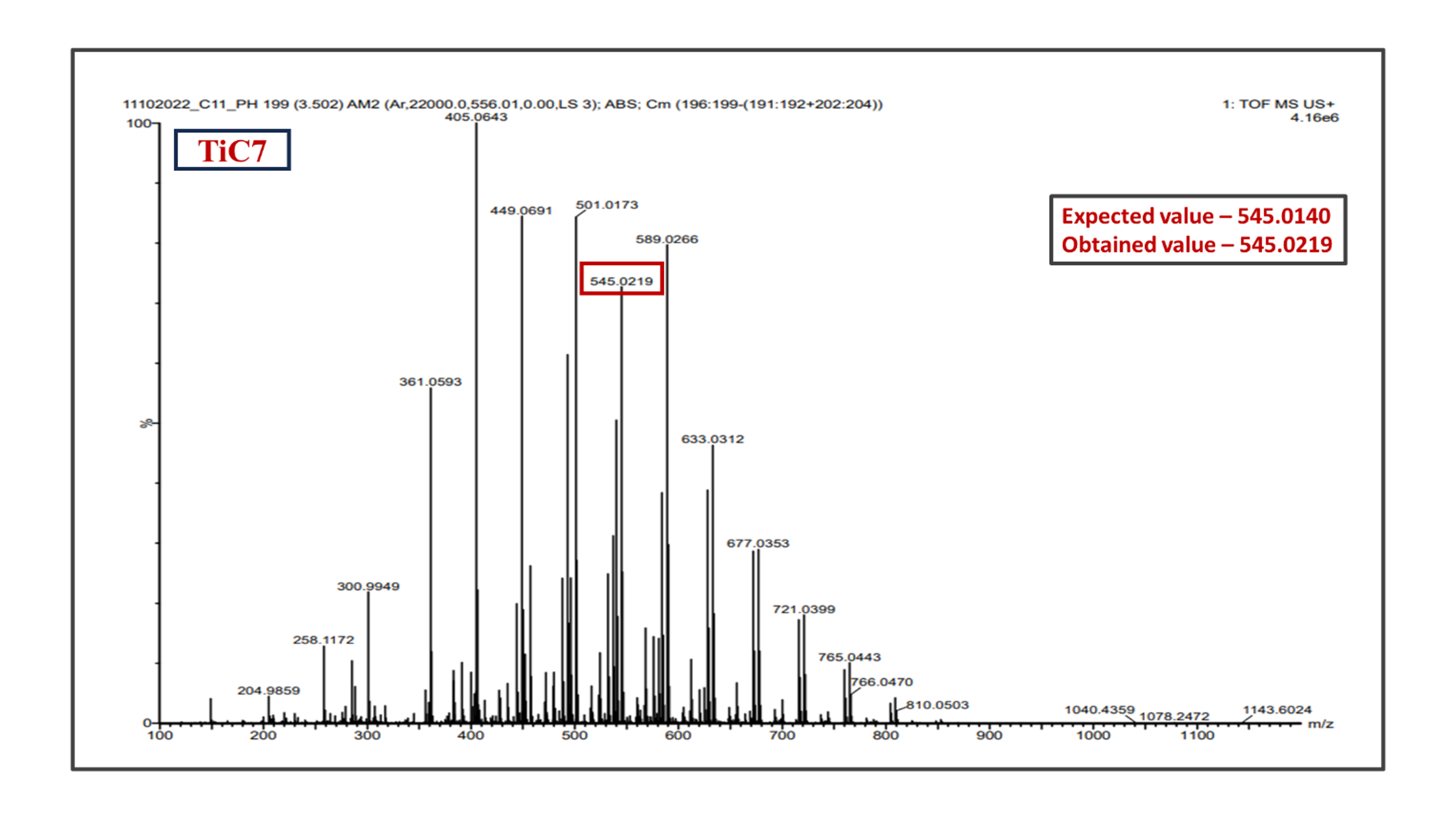


**Figure.S42. Electrospray ionization ESI‐MS (m/z) spectrum of TiC7**


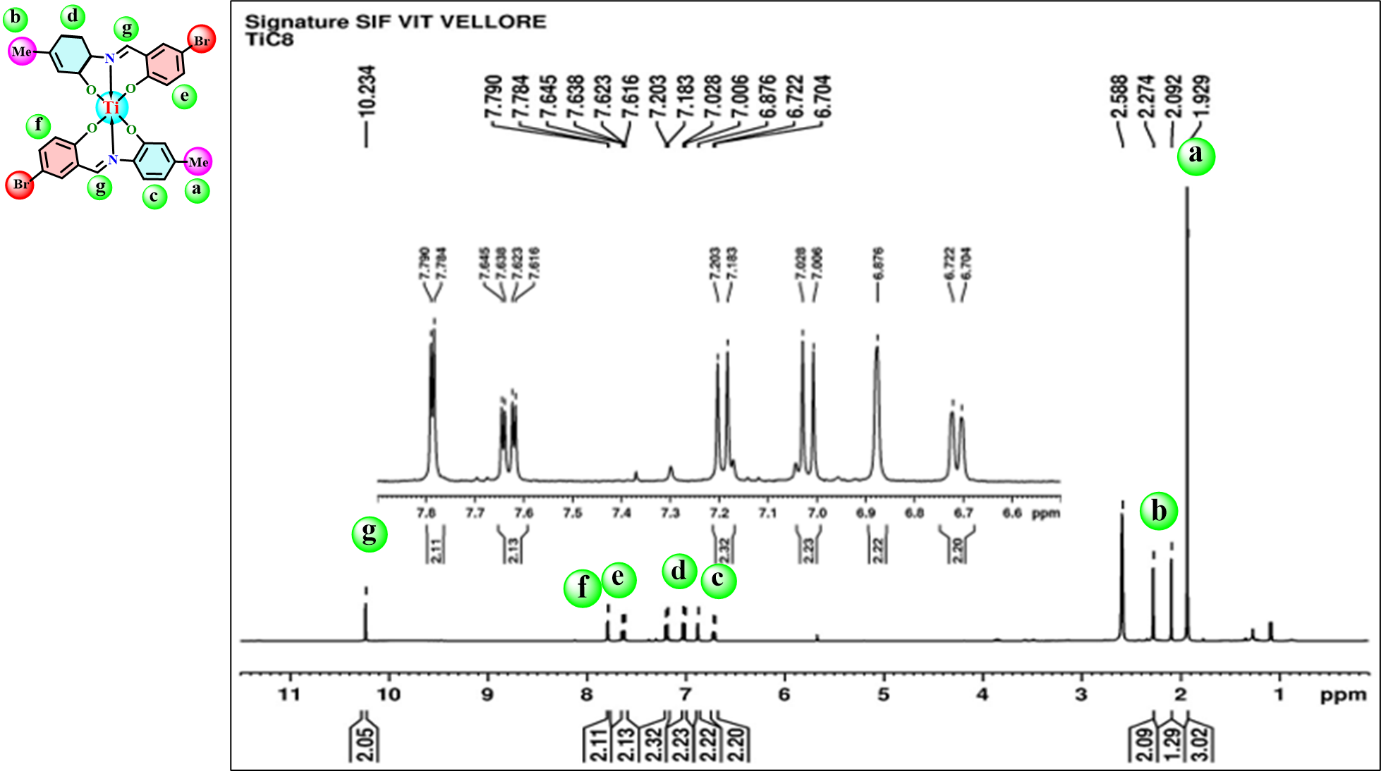


**Figure.S43. ^1^H NMR spectrum of TiC8(400 MHz, DMSO-d_6_)**


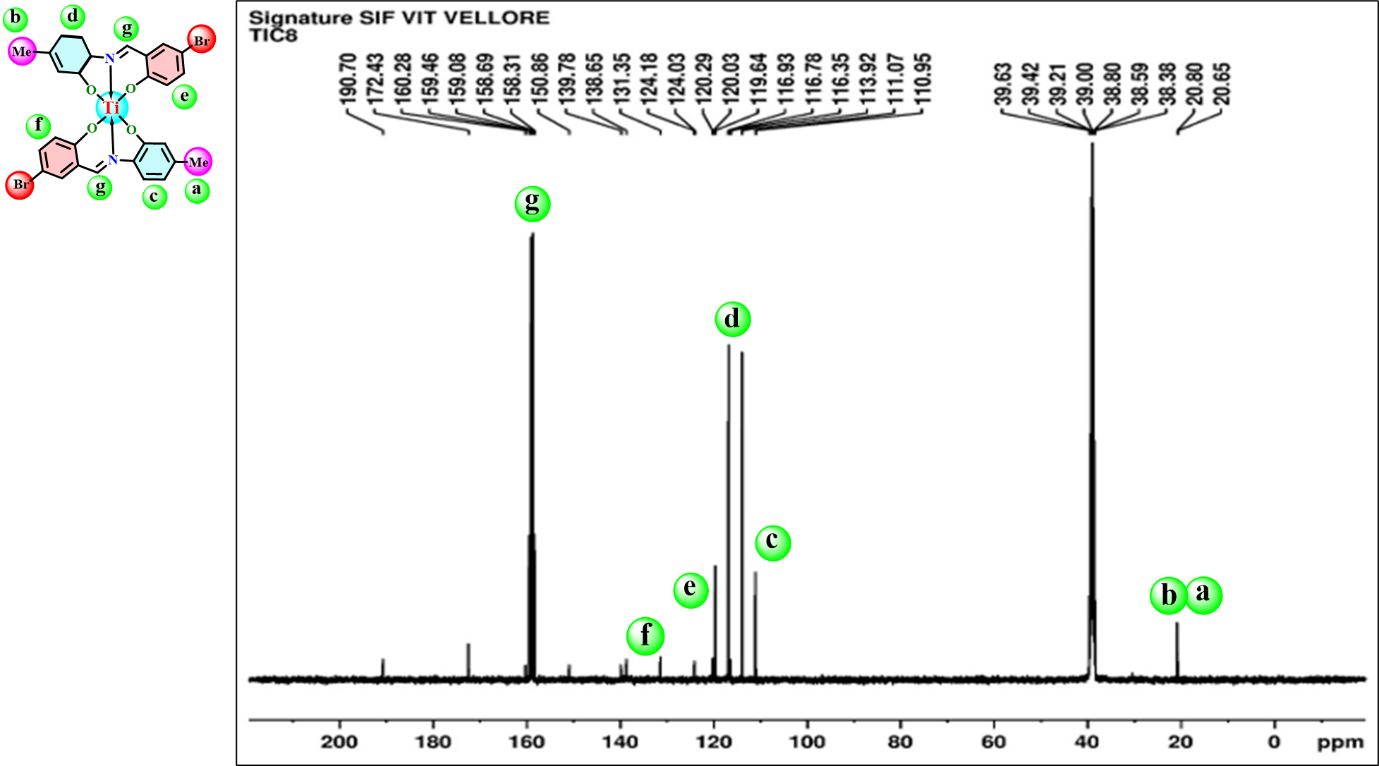


**Figure.S44. ^13^C NMR spectrum of TiC8(400 MHz, DMSO-d_6_)**


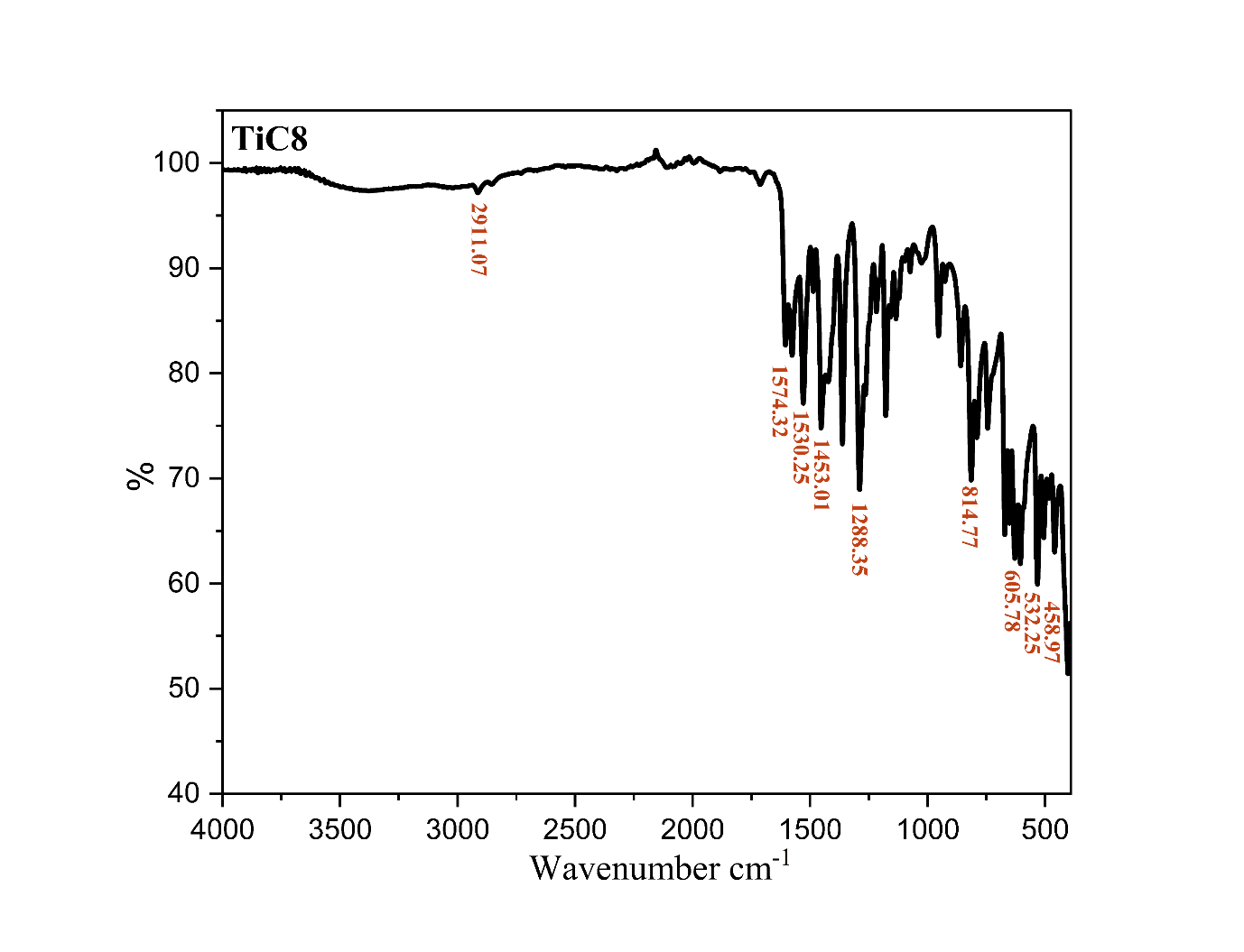
**Figure.S45. FT- IR spectrum of TiC8**


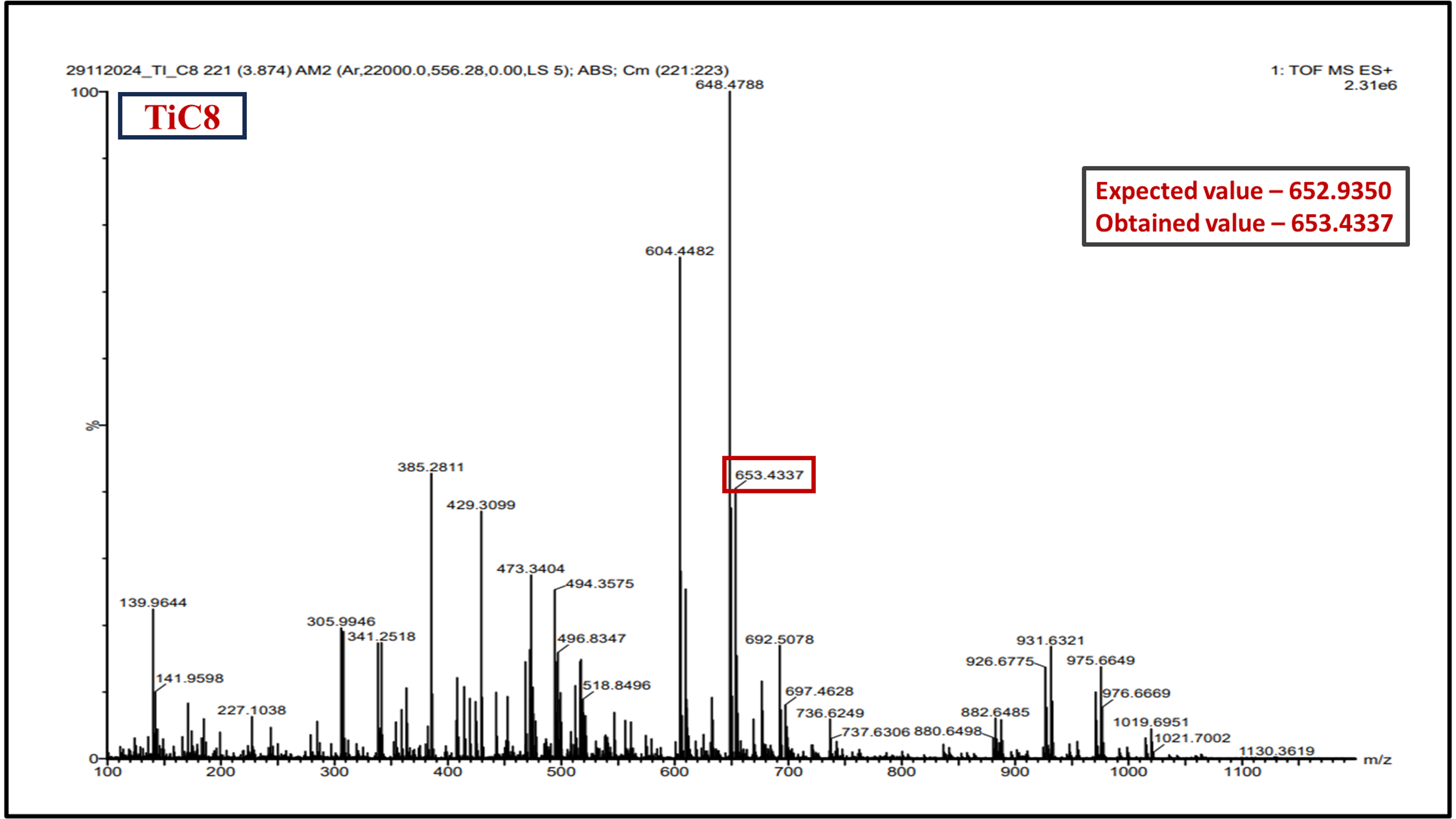


**Figure.S46. Electrospray ionization ESI‐MS (m/z) spectrum of TiC8**


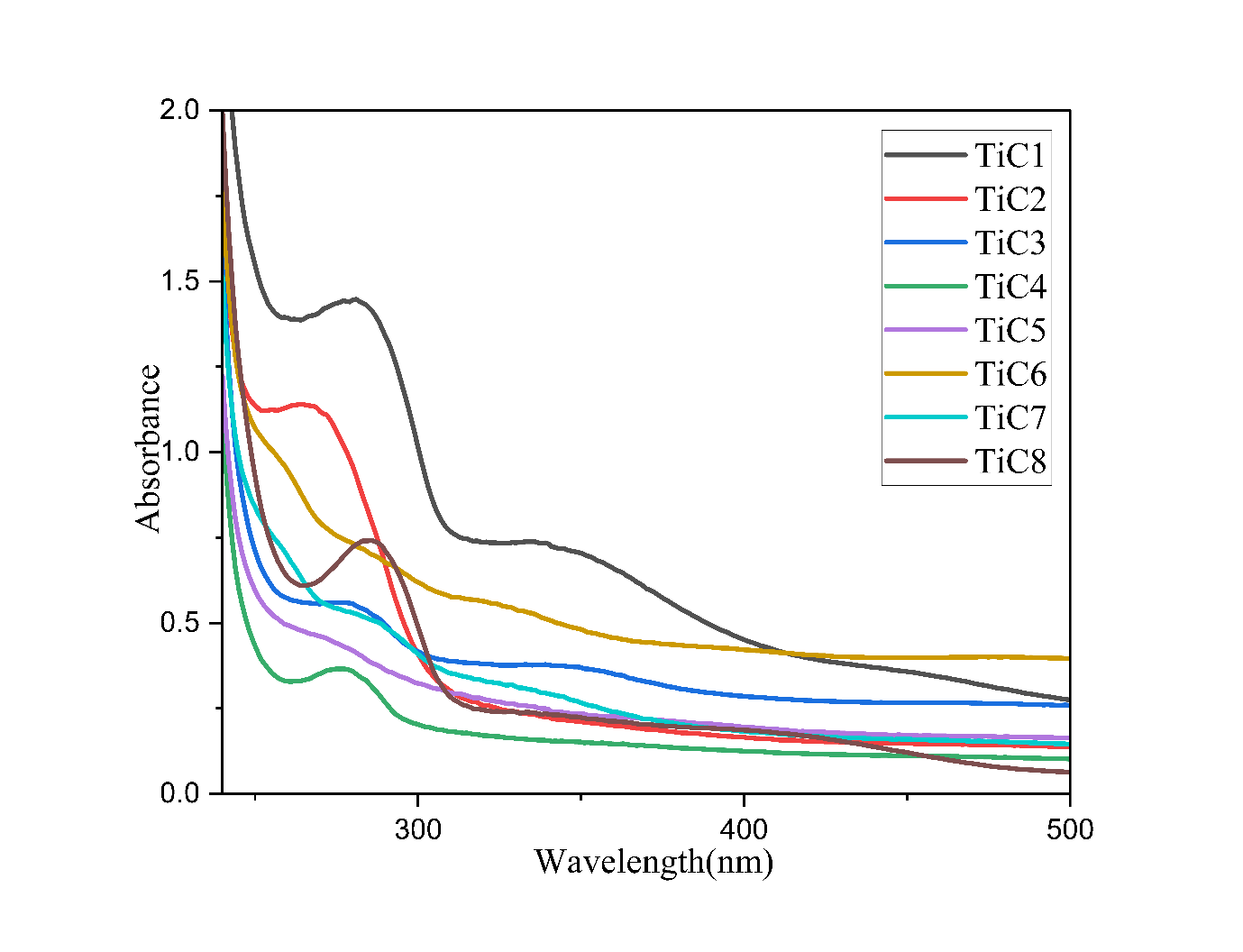



 **Fig.S47. UV-Vis Spectra of** **Ti(IV) complexes in DMSO: H_2_O (1:9)**

**Fig.S48. Fluorescence Spectra of Ti(IV) complexes in DMSO: H_2_O (1:9)**

















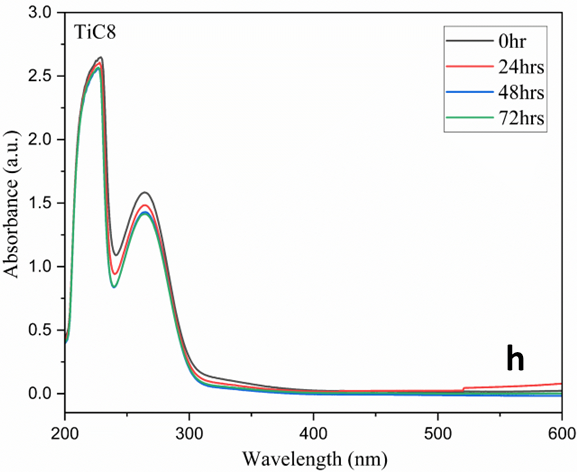


**Fig.S49. UV-Vis stability study of Ti(IV) complexes in 1:9 DMSO: H_2_O medium**











**
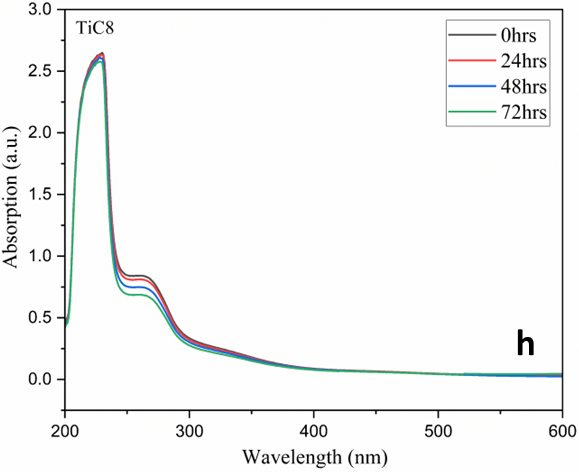
**
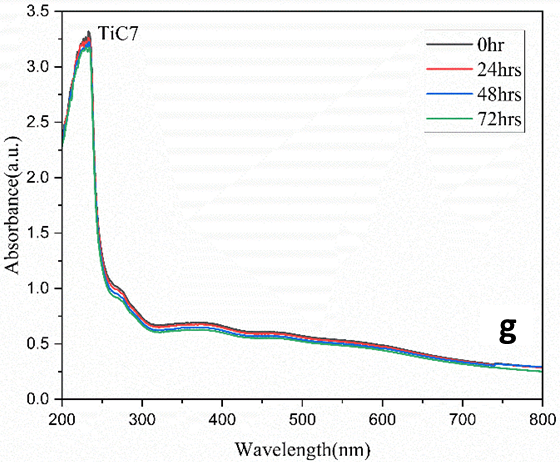






**Fig.S50. UV-Vis stability study of Ti(IV) complexes in GSH medium**

















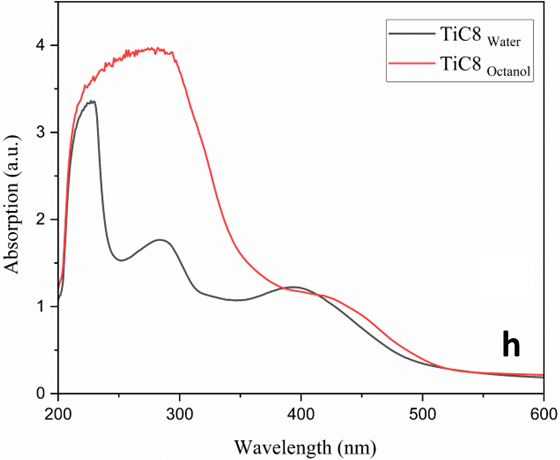




**Fig. S51. UV-visible spectra of Ti(IV) complexes for Lipophilicity study of complexes in octanol: water**


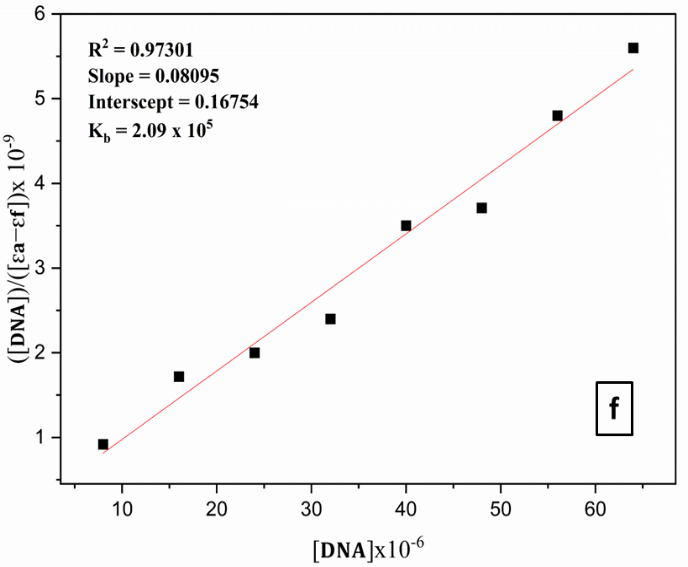

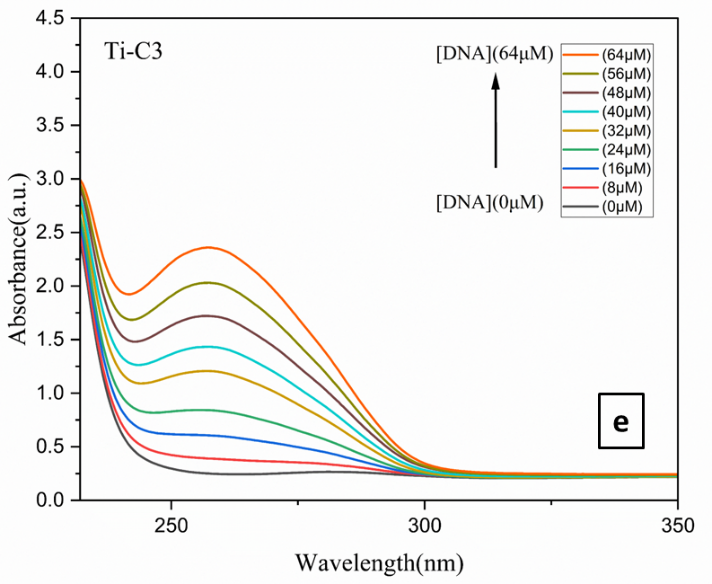

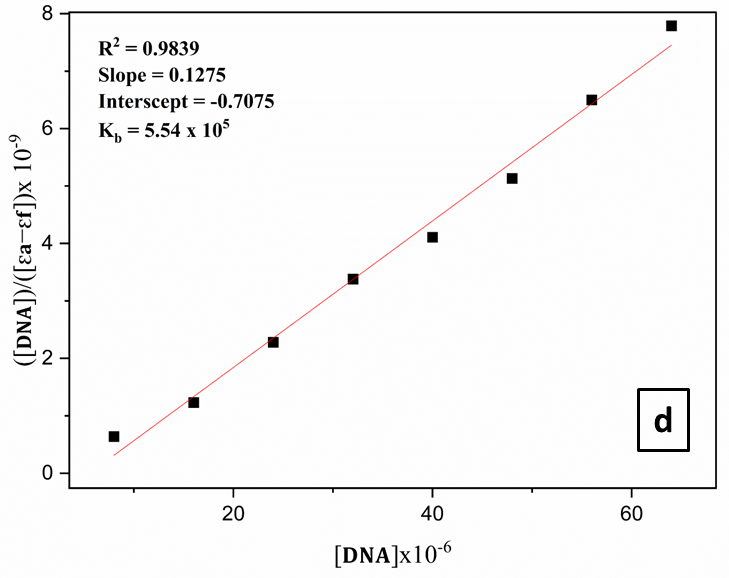

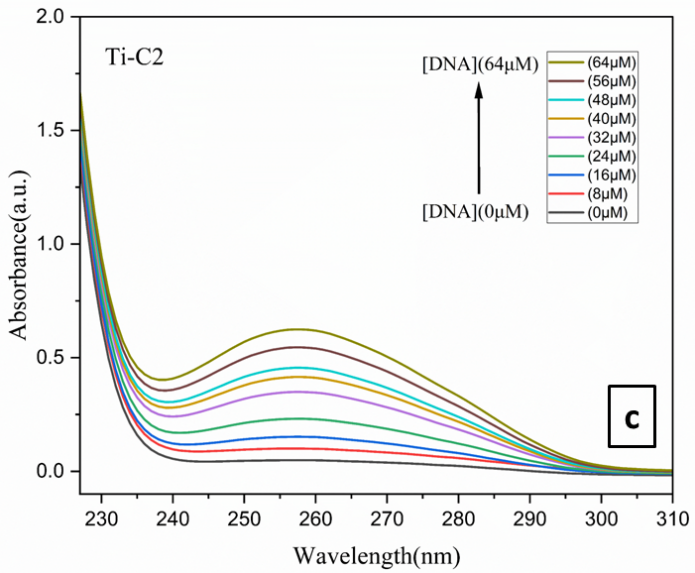

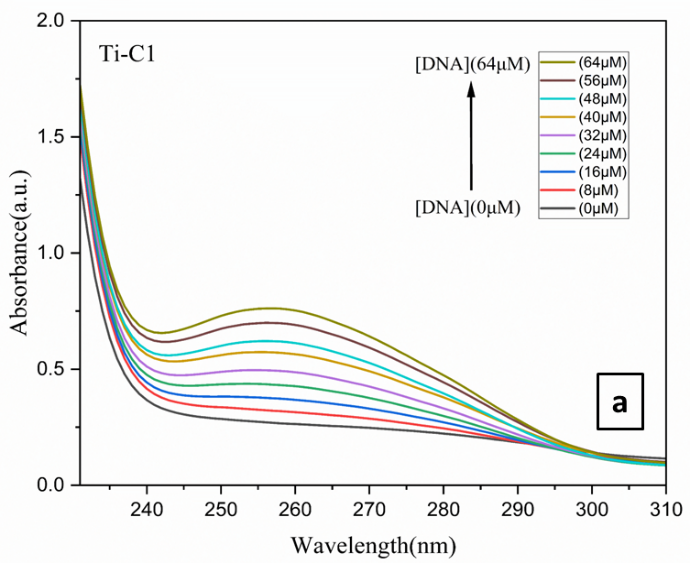

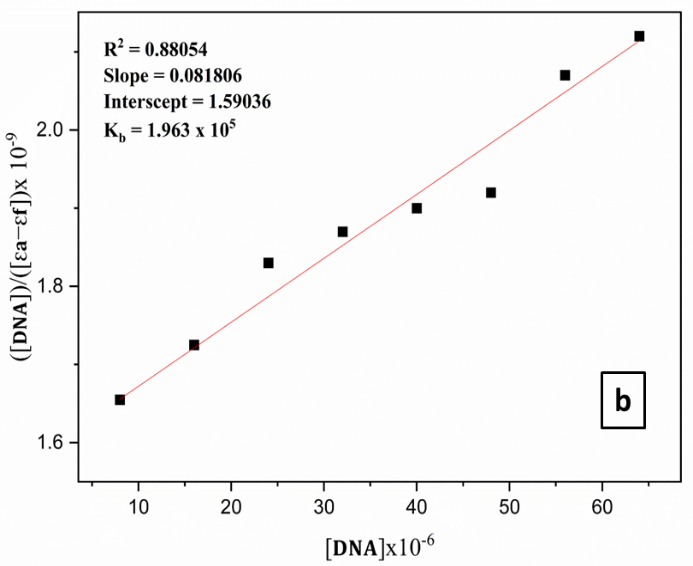


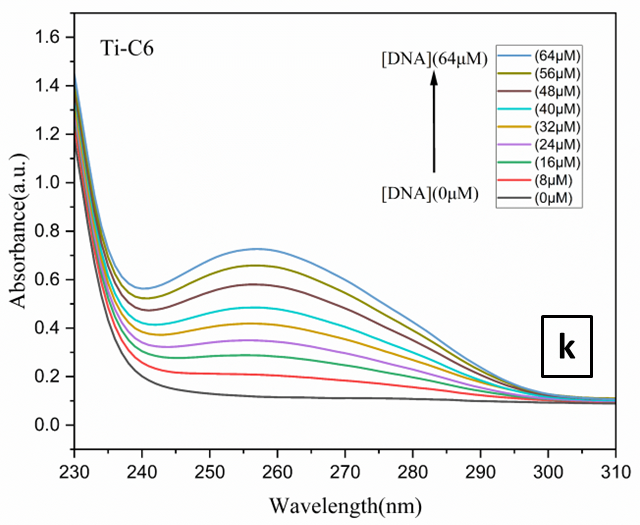

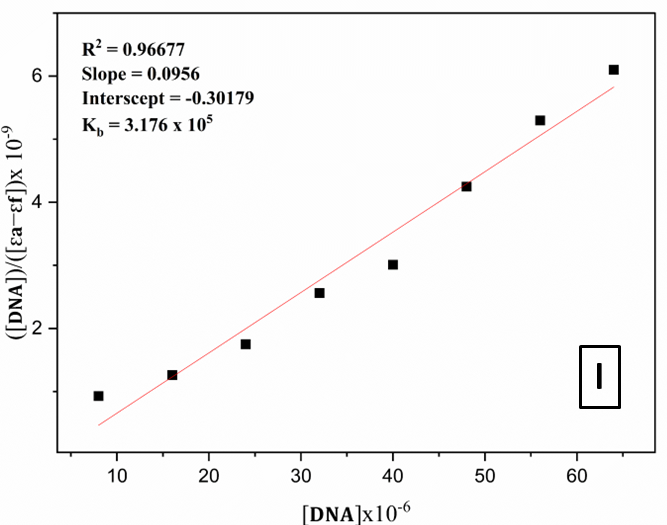

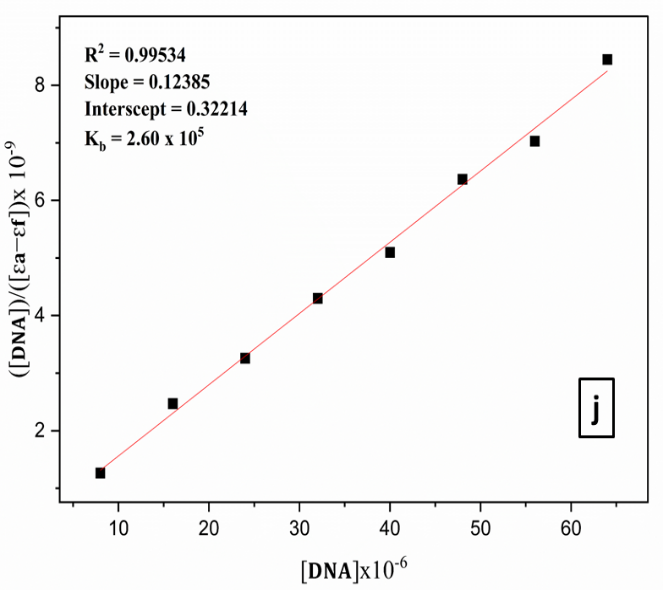

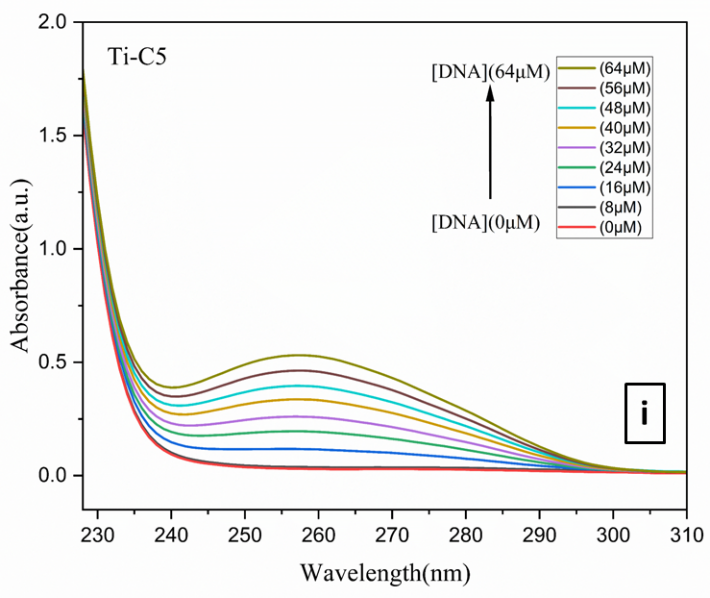

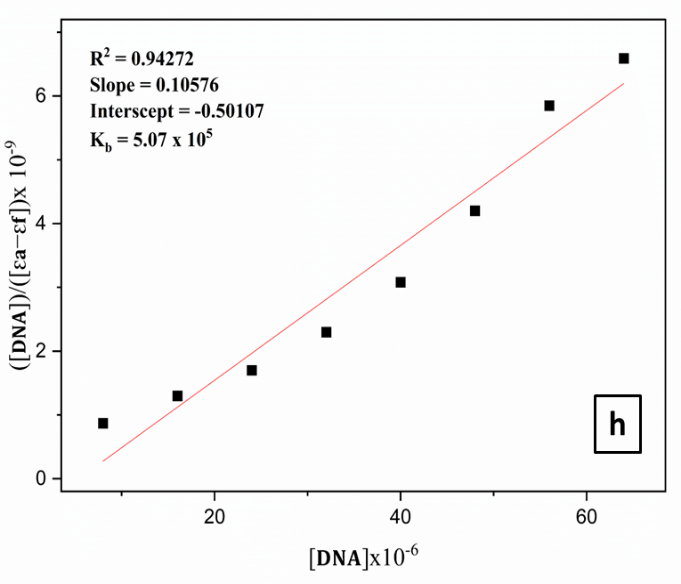

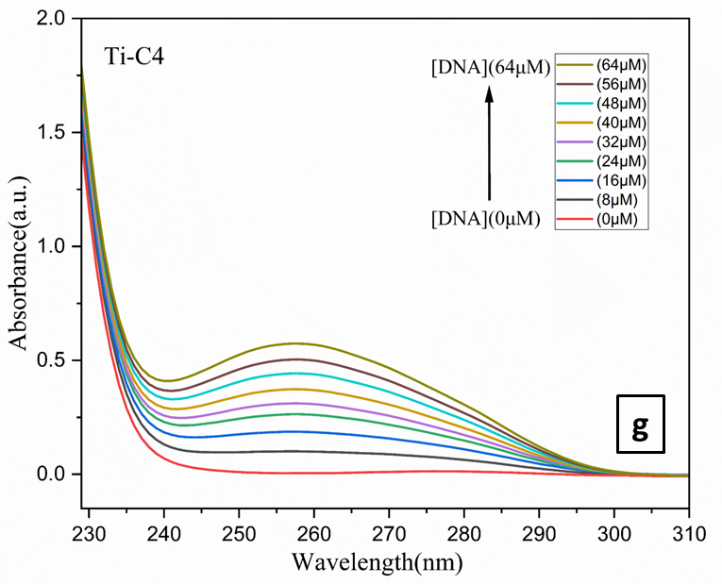


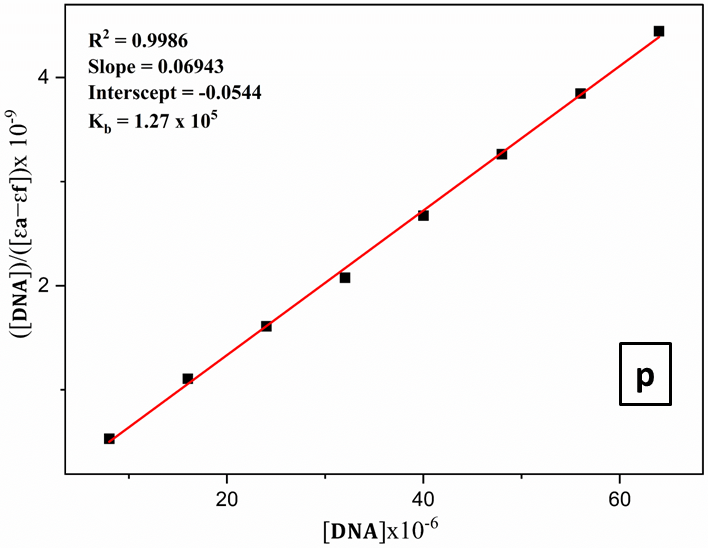

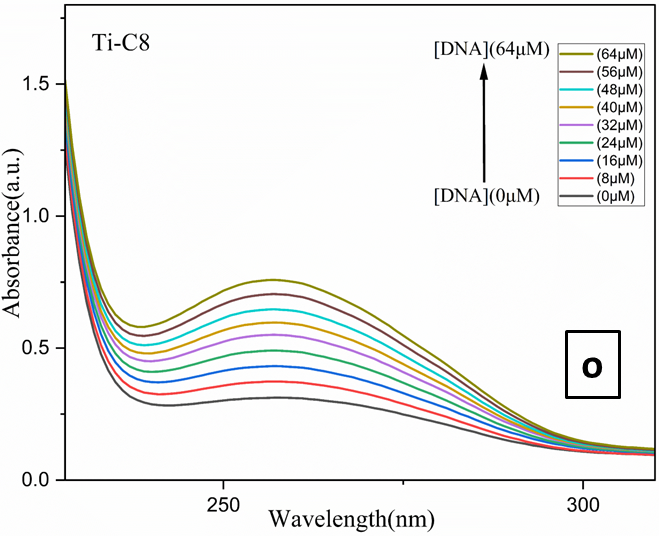

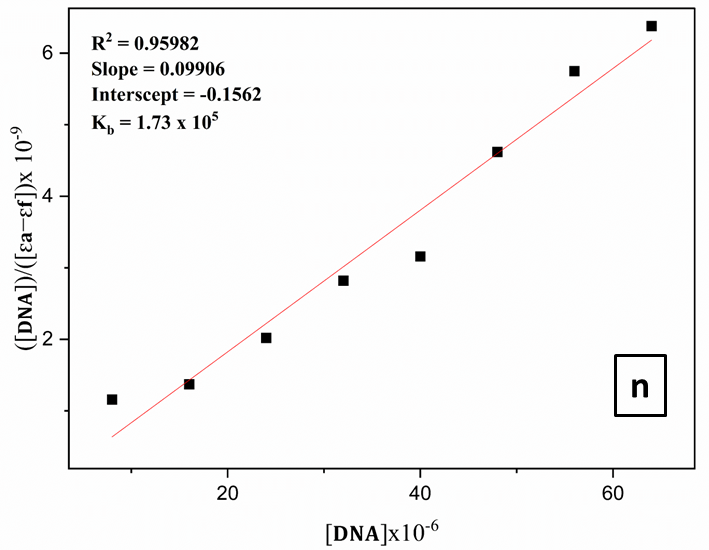

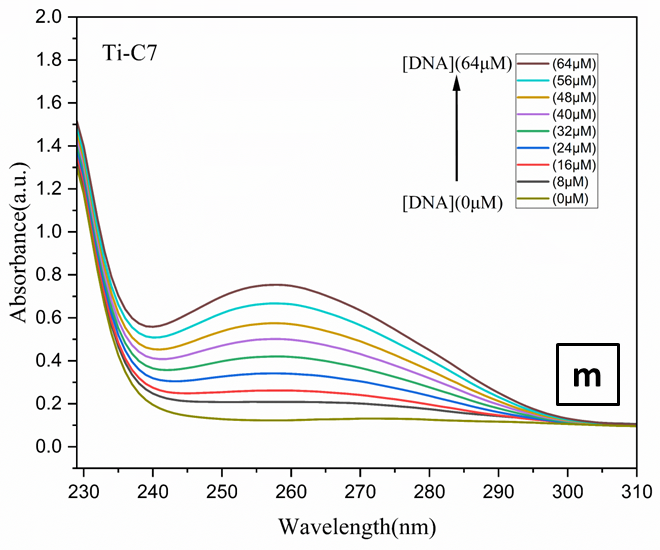


**Fig.S52. UV-Visible spectra of DNA Binding studies** **(0–64 µM) of Ti(IV) complexes( a, c, e, g, i, k, m and o ) their respective linear plots ( b, d, f, h, j, l, n and p )**


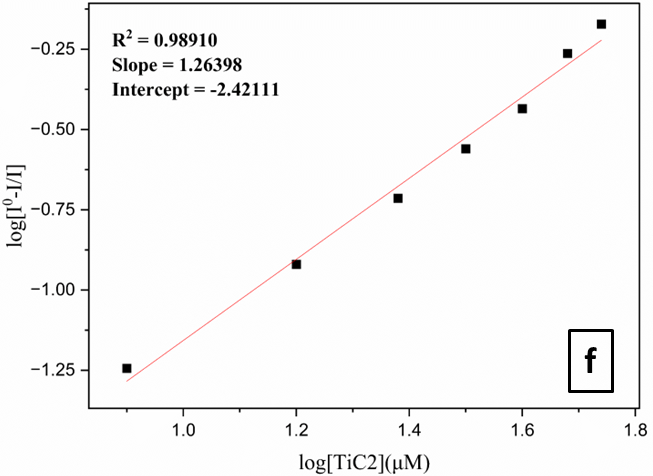

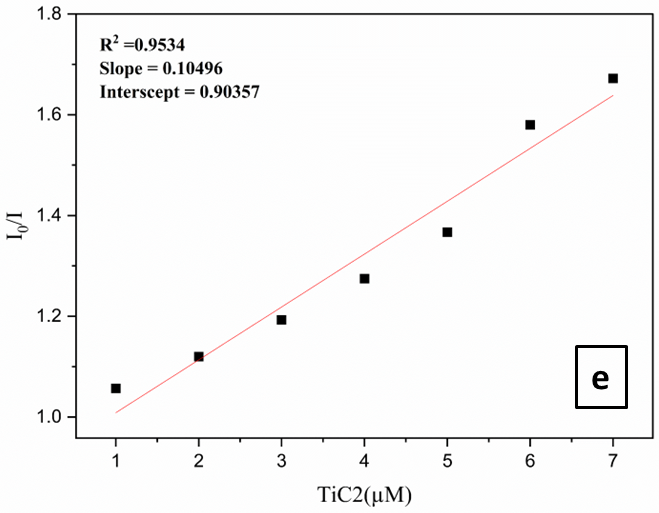

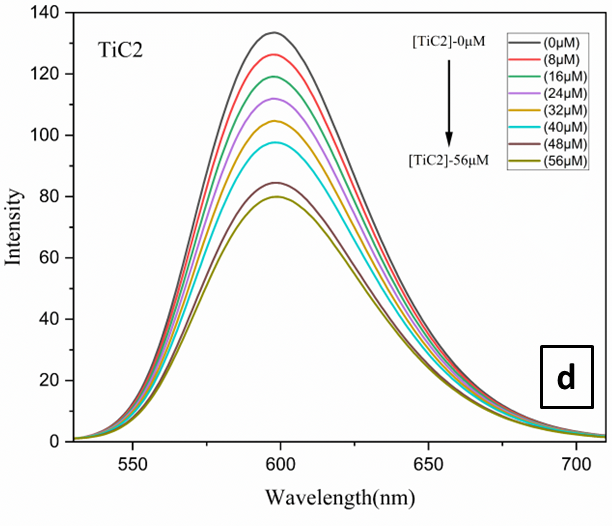

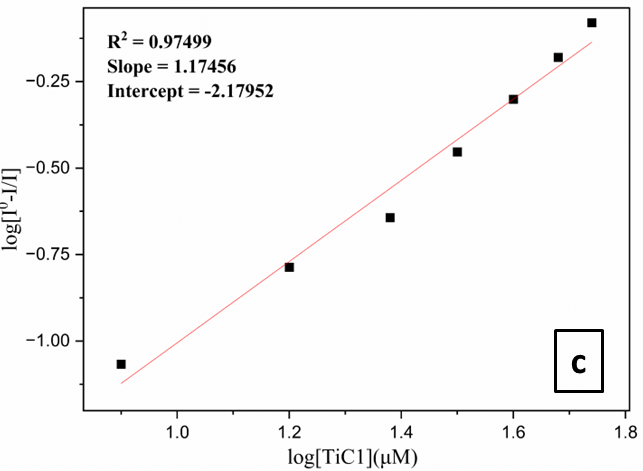

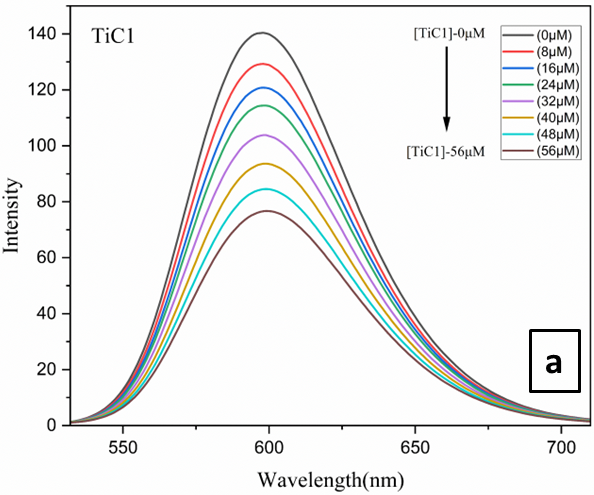

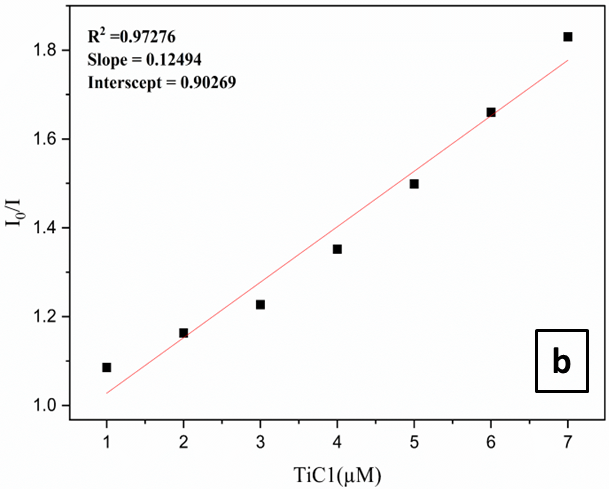


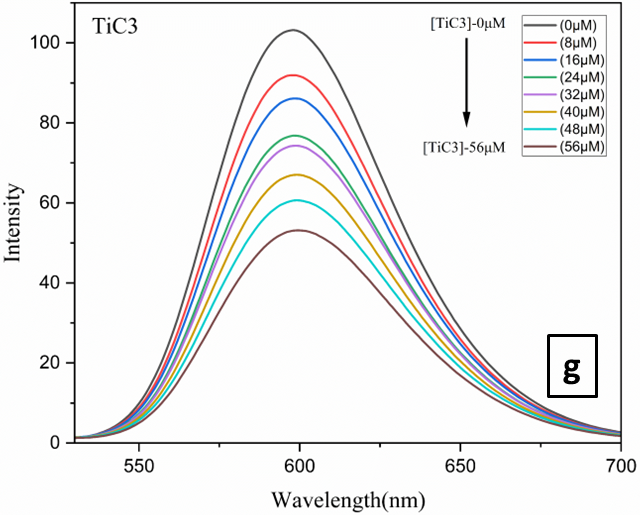

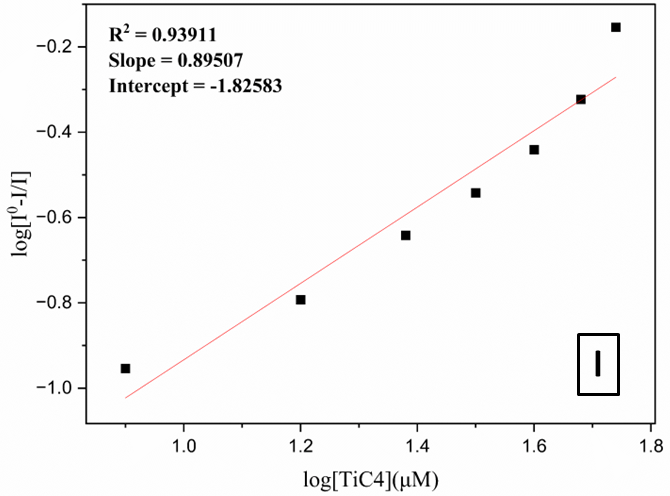

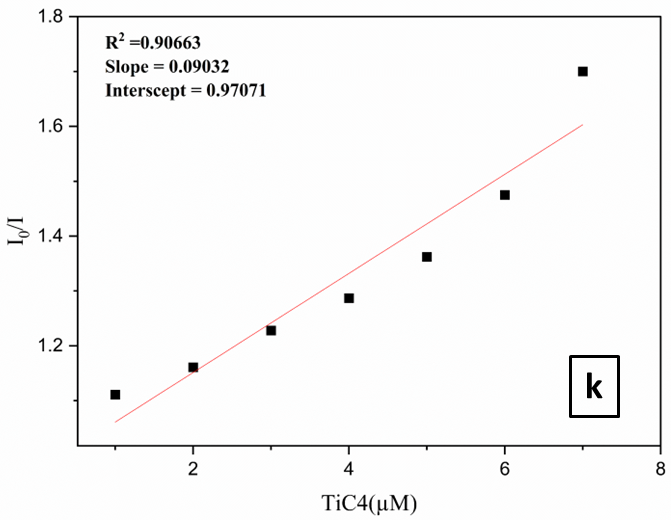

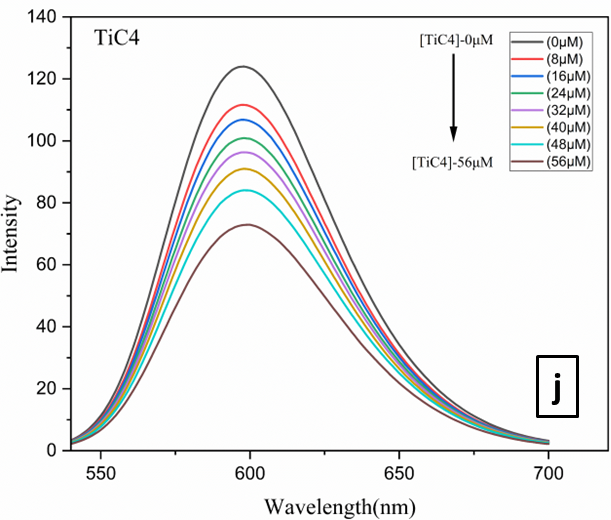


**Fig.S53. Fluorescence quenching spectra of DNA with increasing concentration of Ti(IV) complexes** **(0–56 µM) (a)TiC1, (d) TiC2, (g) TiC3, (j) TiC4, (m) TiC5, (p) TiC6,** **(s) TiC7 and (v) TiC8. Stern-Volmer plots of I_0_/I vs. complex (b)TiC1, (e) TiC2, (h) TiC3, (k) TiC4, (n) TiC5, (q) TiC6, (t) TiC7 and (w) TiC8. Modified stern Volmer/Scatchard plot of log([I_0_-I]/I) vs. log [complex] (c)TiC1, (f) TiC2, (i) TiC3, (l) TiC4, (o) TiC5, (r) TiC6, (u) TiC7 and (x) TiC8**

**Fig.S54. Viscosity Studies of Ti(IV) complexes**

**Fig.S55. Cyclic Voltammograms of Ti(IV) complexes with CT-DNA(0–50 µM).**

**Table.S1. Oxidation and reduction peaks obtained from Cyclic Voltammograms of Ti(IV) complexes with CT-DNA(0–50 µM).**

| Complexes | Oxidation peak | Reduction peak |
| --- | --- | --- |
| TiC1 | 0.058, 0.75 | -0.32, -1.02 |
| TiC2 | 1.18 | -0.14, -0.89 |
| TiC3 | 0.47, 1.14 | -0.23, -0.9 |
| TiC4 | 0.40, 1.12 | -0.12, -1.0 |
| TiC5 | 0.25, 1.23 | -0.12, -0.8, |
| TiC6 | 0.6, 1.15 | -0.22, -1.1 |
| TiC7 | 0.5, 1.12 | -0.15, -1.0 |
| TiC8 | 0.49 | -0.21, -1.0 |

**Fig.S56. Fluorescence quenching spectra of BSA with increasing concentration of Ti(IV) complexes (0–56 µM) (a)TiC1, (d) TiC2, (g) TiC3, (j) TiC4, (m) TiC5, (p) TiC6 (s) TiC7, and (v) TiC8. Stern-Volmer plots of I_0_/I vs. complex (b)TiC1, (e) TiC2, (h) TiC3, (k) TiC4, (n) TiC5, (q) TiC6, (t) TiC7 and (v) TiC8. Scatchard plot of log([I_0_-I]/I) vs. log [complex] (c)TiC1, (f) TiC2, (i) TiC3, (l) TiC4, (o) TiC5, (r) TiC6, (u) TiC7 and (x) TiC8**

**Fig.S57. Synchronous spectra of BSA with increasing concentration of Ti(IV) complexes (0–56 µM) at Δλ=15 nm, (a)TiC1, (c) TiC2, (e) TiC3, (g) TiC4, (i) TiC5, (k) TiC6, (m) TiC7 and (o) TiC8. Stern-Volmer plots of I_0_/I vs. complex (b)TiC1, (d) TiC2, (f) TiC3, (h) TiC4, (j) TiC5, (l) TiC6, (n) TiC7 and (p) TiC8.**

**Fig.S58. Synchronous spectra of BSA with increasing concentration of Ti(IV) complexes (0–56 µM) at Δλ=60 nm, (a)TiC1, (c) TiC2, (e) TiC3, (g) TiC4, (i) TiC5, (k) TiC6, (m) TiC7 and (o) TiC8. Stern-Volmer plots of I_0_/I vs. complex (b)TiC1, (d) TiC2, (f) TiC3, (h) TiC4, (j) TiC5, (l) TiC6, (n) TiC7 and (p) TiC8.**

**Fig.S59. Site marker fluorescence quenching studies of BSA+Ibuprofin with an increase in the concentration of Ti(IV) complexes (0–80 µM) , (a)TiC1, (c) TiC2, (e) TiC3, (g) TiC4, (i) TiC5, (k) TiC6, (m) TiC7 and (o) TiC8. Scatchard plot of log([I_0_-I]/I) vs log [complex] (b)TiC1, (d) TiC2, (f) TiC3, (h) TiC4, (j) TiC5, (l) TiC6, (n) TiC7 and (p) TiC8.**

**Fig.S60. Site marker fluorescence quenching studies of BSA+Warfarin with an increase in the concentration of Ti(IV) complexes (0–80 µM) , (a)TiC1, (c) TiC2, (e) TiC3, (g) TiC4, (i) TiC5, (k) TiC6, (m) TiC7 and (o) TiC8. Scatchard plot of log([I_0_-I]/I) vs log [complex] (b)TiC1, (d) TiC2, (f) TiC3, (h) TiC4, (j) TiC5, (l) TiC6, (n) TiC7 and (p) TiC8.**

**Fig.S61 Molecular docking of (TiC1) complexes with BSA**

**Fig.S62 Molecular docking of (TiC2) complexes with BSA**

**Fig.S63 Molecular docking of (TiC3) complexes with BSA**

**Fig.S64 Molecular docking of (TiC4) complexes with BSA**

**Fig.S65 Molecular docking of (TiC5) complexes with BSA**

**Fig.S66 Molecular docking of (TiC6) complexes with BSA**

**Fig.S67 Molecular docking of (TiC7) complexes with BSA**

**Fig.S68 Molecular docking of (TiC8) complexes with BSA**

**Fig.S69. Molecular docking of Ti(IV) complexes with DNA**

**Table S2; Bond length (Å) of Ti(IV) complexes**

| **S. NO** | **Code** | **O_Br_---Ti** | **O_M_---Ti** | **N---Ti** | **O_Ph_---Ti** | **C=O---Ti** | **C-O---Ti** |
| --- | --- | --- | --- | --- | --- | --- | --- |
| 1 | **TiC1** | 1.9670 | 1.9784 | 2.1221 | 1.8497 | 2.0242 | 1.9991 |
| 2 | **TiC2** | 1.9694 | 1.9856 | 2.1057 | 1.9873 | 2.0517 | 1.9851 |
| 3 | **TiC3** | 1.9425 | 1.9466 | 2.1364 | 1.8635 | 2.0268 | 1.9625 |
| 4 | **TiC4** | 1.9992 | 2.0059 | 2.0980 | 1.8667 | 2.0329 | 2.0088 |
| 5 | **TiC5** | 1.9647 | 1.9829 | 2.1237 | 1.8539 | 2.0102 | 2.0122 |
| 6 | **TiC6** | 1.9807 | 1.9941 | 2.1148 | 1.8502 | 2.0355 | 2.0014 |
| 7 | **TiC7** | 1.9966 | 2.0066 | 2.1264 | 1.8631 | 2.0466 | 1.9986 |
| 8 | **TiC8** | 1.9671 | 1.9484 | 2.1312 | - | - | - |

**Table S3; Comparison of experimental and theoretical excitation spectral details.**

| **Code** | **Experimental** | **Theoretical Prediction** | | | |
| --- | --- | --- | --- | --- | --- |
|  | **Abs**  **(nm)** | **Abs. max**  **(nm)** | **Oscillator strength**  **(ƭ)** | **Transition** | **Orbital Contribution** |
| **TiC1** | 390 | 365.01 | 0.3011 | S_0_→S_11_ | H→L+5 46%, H-3→L 24%, H-4→L 21% |
| **TiC2** | 350 | 366.62 | 0.2848 | S_0_→S_14_ | H→L+4 47%, H-3→L+1 27%, H-3→L+2 10% |
| **TiC3** | 340 | 356.1 | 0.2549 | S_0_→S_11_ | H→L+4 33%, H-3→L 24%, H-4→L 31% |
| **TiC4** | 347 | 369.95 | 0.3159 | S_0_→S_11_ | H→L+4 54%, H-3→L 18%, H-4→L 15% |
| **TiC5** | 337 | 351.44 | 0.2621 | S_0_→S_12_ | H-4→L 52%, H→L+4 18%, H-3→L 17% |
| **TiC6** | 390 | 360.32 | 0.2434 | S_0_→S_11_ | H→L+4 29%, H-3→L 23%, H-1→L+2 19%, H-51→L 15% |
| **TiC7** | 343 | 369.93 | 0.2264 | S_0_→S_11_ | H→L+4 36%, H-3→L 23%, H-4→L 27% |
| **TiC8** | 350 | 348.95 | 0.2742 | S_0_→S_19_ | H→L+4 83%, H-4→L+2 4% |

*H – HOMO, L - LUMO

**Fig.S70. DPPH Assay of Ti(IV) complexes**

**Fig.S71. MTT Assay of Ti(IV) complexes on HeLa cell line. All values are expressed as mean ± SEM. ****p< 0.0001 denotes statistical significance as determined by MTT Assay. Error bars indicates standard deviation from three independent experiments**

**Fig.S72. MTT Assay of Ti(IV) complexes on MCF7 cell line.** **All values are** **expressed as mean ± SEM. ****p< 0.0001 denotes statistical significance as determined by MTT Assay. Error bars indicates standard deviation from three independent experiments**

**Fig.S73. MTT Assay of Ligand L1 and Cisplatin on ^a,c^ HeLa and ^b,d^ MCF7 cell line. All values are expressed as mean ± SEM. ****p< 0.0001 denotes statistical significance as determined by MTT Assay. Error bars indicates standard deviation from three independent experiments**

| **Table S4. Sample and crystal data for ASVS01[L1].** |
| --- |

| **Identification code** | ASVS01 | |
| --- | --- | --- |
| **Chemical formula** | C_14_H_12_BrNO_2_ | |
| **Formula weight** | 306.16 g/mol | |
| **Temperature** | 300(2) K | |
| **Wavelength** | 0.71073 Å | |
| **Crystal size** | 0.076 x 0.190 x 0.210 mm | |
| **Crystal habit** | orange block | |
| **Crystal system** | monoclinic | |
| **Space group** | P 1 21/c 1 | |
| **Unit cell dimensions** | a = 11.6080(4) Å | α = 90° |
|  | b = 11.8289(5) Å | β = 114.7710(10)° |
|  | c = 9.8518(4) Å | γ = 90° |
| **Volume** | 1228.28(8) Å^3^ |  |
| **Z** | 4 | |
| **Density (calculated)** | 1.656 g/cm^3^ | |
| **Absorption coefficient** | 3.339 mm^-1^ | |
| **F(000)** | 616 | |

| **Table S5. Data collection and structure refinement for ASVS01[L1].** |
| --- |

| **Theta range for data collection** | 1.93 to 28.30° | |
| --- | --- | --- |
| **Index ranges** | -15<=h<=15, -15<=k<=15, -13<=l<=13 | |
| **Reflections collected** | 27676 | |
| **Independent reflections** | 3025 [R(int) = 0.0402] | |
| **Coverage of independent reflections** | 98.9% | |
| **Absorption correction** | Multi-Scan | |
| **Max. and min. transmission** | 0.7850 and 0.5410 | |
| **Structure solution technique** | direct methods | |
| **Structure solution program** | SHELXT 2018/2 (Sheldrick, 2018) | |
| **Refinement method** | Full-matrix least-squares on F^2^ | |
| **Refinement program** | SHELXL-2018/3 (Sheldrick, 2018) | |
| **Function minimized** | Σ w(F_o_^2^ - F_c_^2^)^2^ | |
| **Data / restraints / parameters** | 3025 / 0 / 170 | |
| **Goodness-of-fit on F^2^** | 1.081 | |
| **Δ/σ_max_** | 0.001 | |
| **Final R indices** | 2410 data; I>2σ(I) | R1 = 0.0293, wR2 = 0.0692 |
|  | all data | R1 = 0.0431, wR2 = 0.0733 |
| **Weighting scheme** | w=1/[σ^2^(F_o_^2^)+(0.0322P)^2^+0.3923P] where P=(F_o_^2^+2F_c_^2^)/3 | |
| **Largest diff. peak and hole** | 0.256 and -0.261 eÅ^-3^ | |
| **R.M.S. deviation from mean** | 0.051 eÅ^-3^ | |

| **Table S6. Atomic coordinates and equivalent isotropic atomic displacement parameters (Å^2^) for ASVS01[L1].** |
| --- |
| U(eq) is defined as one third of the trace of the orthogonalized U_ij_ tensor. |
|  |

|  | **x/a** | **y/b** | **z/c** | **U(eq)** |
| --- | --- | --- | --- | --- |
| Br1 | 0.91939(2) | 0.57717(2) | 0.17041(3) | 0.04892(10) |
| O1 | 0.62036(15) | 0.91925(10) | 0.34148(18) | 0.0452(4) |
| O2 | 0.39500(16) | 0.92119(11) | 0.4755(2) | 0.0491(4) |
| N1 | 0.49404(14) | 0.75519(14) | 0.38350(18) | 0.0333(3) |
| C1 | 0.65423(16) | 0.72726(15) | 0.2966(2) | 0.0324(4) |
| C2 | 0.72299(18) | 0.64836(16) | 0.2524(2) | 0.0357(4) |
| C3 | 0.82169(17) | 0.68343(16) | 0.2222(2) | 0.0354(4) |
| C4 | 0.85457(19) | 0.79802(18) | 0.2327(2) | 0.0427(5) |
| C5 | 0.7882(2) | 0.87603(17) | 0.2731(2) | 0.0430(5) |
| C6 | 0.68446(18) | 0.84505(16) | 0.3058(2) | 0.0351(4) |
| C7 | 0.55714(17) | 0.68743(16) | 0.3363(2) | 0.0351(4) |
| C8 | 0.39921(16) | 0.72765(15) | 0.4326(2) | 0.0307(4) |
| C9 | 0.34908(17) | 0.81749(15) | 0.4821(2) | 0.0325(4) |
| C10 | 0.25615(17) | 0.79678(17) | 0.5325(2) | 0.0364(4) |
| C11 | 0.21260(17) | 0.68895(17) | 0.5355(2) | 0.0359(4) |
| C12 | 0.2634(2) | 0.60053(17) | 0.4861(2) | 0.0426(5) |
| C13 | 0.35608(19) | 0.61919(17) | 0.4349(2) | 0.0417(5) |
| C14 | 0.1107(2) | 0.6683(2) | 0.5900(3) | 0.0494(5) |

| **Table S7. Bond lengths (Å) for ASVS01[L1].** |
| --- |

| Br1-C3 | 1.9003(18) | O1-C6 | 1.291(2) |
| --- | --- | --- | --- |
| O2-C9 | 1.350(2) | N1-C7 | 1.296(2) |
| N1-C8 | 1.413(2) | C1-C2 | 1.410(2) |
| C1-C7 | 1.420(2) | C1-C6 | 1.430(3) |
| C2-C3 | 1.364(3) | C3-C4 | 1.400(3) |
| C4-C5 | 1.364(3) | C5-C6 | 1.419(3) |
| C8-C13 | 1.381(3) | C8-C9 | 1.394(2) |
| C9-C10 | 1.385(2) | C10-C11 | 1.377(3) |
| C11-C12 | 1.385(3) | C11-C14 | 1.510(3) |
| C12-C13 | 1.384(3) |  |  |

| **Table S8. Bond angles (°) for ASVS01[L1].** |
| --- |

| C7-N1-C8 | 128.19(17) | C2-C1-C7 | 118.87(17) |
| --- | --- | --- | --- |
| C2-C1-C6 | 120.58(16) | C7-C1-C6 | 120.52(16) |
| C3-C2-C1 | 120.13(17) | C2-C3-C4 | 120.41(17) |
| C2-C3-Br1 | 120.53(14) | C4-C3-Br1 | 119.04(13) |
| C5-C4-C3 | 120.46(17) | C4-C5-C6 | 121.88(19) |
| O1-C6-C5 | 121.80(17) | O1-C6-C1 | 121.69(16) |
| C5-C6-C1 | 116.50(17) | N1-C7-C1 | 121.80(17) |
| C13-C8-C9 | 119.66(16) | C13-C8-N1 | 124.03(16) |
| C9-C8-N1 | 116.31(16) | O2-C9-C10 | 124.09(17) |
| O2-C9-C8 | 116.40(16) | C10-C9-C8 | 119.50(17) |
| C11-C10-C9 | 121.32(17) | C10-C11-C12 | 118.53(17) |
| C10-C11-C14 | 120.48(18) | C12-C11-C14 | 120.99(18) |
| C13-C12-C11 | 121.19(18) | C8-C13-C12 | 119.79(18) |

| **Table S9. Anisotropic atomic displacement parameters (Å^2^) for ASVS01[L1].** |
| --- |
| The anisotropic atomic displacement factor exponent takes the form: -2π^2^[ h^2^ a^*2^ U_11_ + ... + 2 h k a^*^ b^*^ U_12_ ] |

|  | **U_11_** | **U_22_** | **U_33_** | **U_23_** | **U_13_** | **U_12_** |
| --- | --- | --- | --- | --- | --- | --- |
| Br1 | 0.04994(15) | 0.04793(15) | 0.06448(17) | 0.00034(10) | 0.03932(12) | 0.00892(9) |
| O1 | 0.0504(8) | 0.0330(8) | 0.0663(10) | -0.0077(6) | 0.0384(7) | -0.0018(6) |
| O2 | 0.0628(10) | 0.0288(8) | 0.0776(12) | -0.0093(7) | 0.0509(9) | -0.0072(7) |
| N1 | 0.0330(8) | 0.0308(8) | 0.0424(9) | -0.0044(7) | 0.0219(7) | -0.0050(6) |
| C1 | 0.0322(9) | 0.0338(9) | 0.0357(10) | -0.0033(8) | 0.0186(8) | -0.0022(7) |
| C2 | 0.0394(10) | 0.0315(10) | 0.0425(11) | -0.0023(8) | 0.0231(9) | -0.0015(8) |
| C3 | 0.0369(10) | 0.0379(10) | 0.0385(10) | 0.0013(8) | 0.0228(8) | 0.0055(8) |
| C4 | 0.0410(10) | 0.0459(12) | 0.0531(12) | 0.0043(10) | 0.0315(10) | -0.0021(9) |
| C5 | 0.0496(12) | 0.0334(10) | 0.0583(13) | 0.0006(9) | 0.0346(10) | -0.0045(9) |
| C6 | 0.0377(10) | 0.0349(10) | 0.0391(10) | -0.0023(8) | 0.0224(8) | 0.0000(8) |
| C7 | 0.0342(9) | 0.0325(10) | 0.0447(11) | -0.0042(8) | 0.0227(8) | -0.0031(8) |
| C8 | 0.0284(8) | 0.0325(9) | 0.0361(10) | -0.0017(8) | 0.0185(8) | -0.0020(7) |
| C9 | 0.0348(9) | 0.0300(9) | 0.0359(10) | -0.0020(8) | 0.0179(8) | -0.0025(7) |
| C10 | 0.0372(10) | 0.0390(10) | 0.0392(10) | -0.0019(8) | 0.0222(8) | 0.0038(8) |
| C11 | 0.0333(9) | 0.0440(11) | 0.0359(10) | 0.0018(8) | 0.0198(8) | -0.0010(8) |
| C12 | 0.0456(11) | 0.0340(10) | 0.0585(13) | 0.0002(9) | 0.0320(10) | -0.0075(8) |
| C13 | 0.0449(11) | 0.0313(10) | 0.0592(13) | -0.0068(9) | 0.0321(10) | -0.0023(8) |
| C14 | 0.0471(12) | 0.0592(14) | 0.0561(13) | 0.0027(11) | 0.0354(11) | -0.0043(10) |

| **Table S10. Hydrogen atomic coordinates and isotropic atomic displacement parameters (Å^2^) for ASVS01[L1].** |
| --- |

|  | **x/a** | **y/b** | **z/c** | **U(eq)** |
| --- | --- | --- | --- | --- |
| H1 | 0.380(3) | 0.961(3) | 0.522(3) | 0.074000 |
| H3 | 0.517(2) | 0.823(2) | 0.382(2) | 0.050000 |
| H2 | 0.7010 | 0.5722 | 0.2439 | 0.043000 |
| H4 | 0.9221 | 0.8211 | 0.2120 | 0.051000 |
| H5 | 0.8117 | 0.9517 | 0.2795 | 0.052000 |
| H7 | 0.5383 | 0.6106 | 0.3283 | 0.042000 |
| H10 | 0.2225 | 0.8569 | 0.5649 | 0.044000 |
| H12 | 0.2347 | 0.5273 | 0.4874 | 0.051000 |
| H13 | 0.3892 | 0.5589 | 0.4022 | 0.050000 |
| H14A | 0.0947 | 0.5886 | 0.5892 | 0.074000 |
| H14B | 0.0342 | 0.7064 | 0.5255 | 0.074000 |
| H14C | 0.1383 | 0.6968 | 0.6900 | 0.074000 |

**References**

1. Thanigachalam, Sathish, and Madhvesh Pathak. "Bioactive O^N^O^ Schiff base appended homoleptic titanium (iv) complexes: DFT, BSA/CT-DNA interactions, molecular docking and antitumor activity against HeLa and A549 cell lines." RSC advances 14.19 (2024): 13062-13082.
2. Salimath, Shivabasayya V., and Madhvesh Pathak. "Copper (II) complexes incorporated with 2, 2-bipyridyl and 2, 2′-(((1E, 1′ E)-1, 4-phenylene-bis (methanylylidene)) bis (azanylylidene)) diphenol derivatives: In-vitro interaction with DNA/BSA, DFT, molecular docking and cytotoxicity." *Inorganic Chemistry Communications* 162 (2024): 112226.
3. Subramaniyan, Mahabarathi, and Madhvesh Pathak. "New bioactive titanium (IV) derivatives with their DFT, molecular docking, DNA/BSA interaction, antioxidant and in-vitro investigations." *Inorganica Chimica Acta* 571 (2024): 122191.
4. Roy, Nilmadhab, et al. "Mitochondria-targeting click-derived pyridinyltriazolylmethylquinoxaline-based Y-shaped binuclear luminescent ruthenium (II) and iridium (III) complexes as cancer theranostic agents." *Inorganic Chemistry* 59.23 (2020): 17689-17711.
5. Balaji, Sundarraman, et al. "Synthesis and structure of arene Ru (II) N∧ O-chelating complexes: in vitro cytotoxicity and cancer cell death mechanism." *Organometallics* 39.8 (2020): 1366-1375.
6. Roy, Nilmadhab, et al. "G2/M-Phase-inhibitory mitochondrial-depolarizing Re (I)/Ru (II)/Ir (III)-2, 2′-bipyrimidine-based heterobimetallic luminescent complexes: An assessment of in vitro antiproliferative activity and bioimaging for targeted therapy toward human TNBC cells." *ACS omega* 8.13 (2023): 12283-12297.
